# Transcriptomic Profiling Provides Insight into the Molecular Basis of Heterosis in Philippine-Reared *Bombyx mori* Hybrids

**DOI:** 10.3390/insects16030243

**Published:** 2025-02-26

**Authors:** Ma. Ysabella Elaine D. Conde, Jose Planta, Ma. Anita M. Bautista

**Affiliations:** National Institute of Molecular Biology and Biotechnology, University of the Philippines Diliman, Quezon City 1101, Philippines; jgplanta@up.edu.ph

**Keywords:** *Bombyx mori*, silkworm, transcriptome analysis, RNA sequencing, differential gene expression, heterosis, hybridization

## Abstract

In the Philippines, *Bombyx mori* parental strains Lat21 and B221 are crossed to yield NC144 and CN144, which demonstrate hybrid vigor. The molecular basis of the observed vigor in the hybrids is warranted, as it may assist in improving local sericulture programs. This study, therefore, aims to investigate the basis of hybrid vigor and generate molecular resources through whole-silkworm larvae transcriptome sequencing, assembly, and analysis. Differential gene expression was also conducted among the parental strains and hybrids. Assembly of the pre-processed reads was also performed using de novo and reference-based protocols. As expected, the reference-based assembly was better than de novo, based on E90N50, N50, and BUSCO assembly completeness metrics. The Analysis of the differentially expressed genes (DEGs) revealed 202 upregulated and 182 downregulated genes in the hybrids (with the parents as the reference) and 66 upregulated and 753 downregulated genes in NC144 (with CN144 as the reference). Among these were genes encoding heat shock proteins and antimicrobial peptides, which may serve as markers for marker-assisted breeding. The genes were further validated using quantitative real-time PCR. Moreover, the inducible nature of these genes under stressors like extreme temperature and bacterial exposure suggests their potential as diagnostic tools for stress assessment.

## Simple Summary

Hybridization among Philippine-reared *Bombyx mori* strains is a common practice locally and is aimed at producing hybrids that exhibit improved silkworm traits and hence enhanced performance due to the phenomenon of heterosis. Despite the observed improvements in these hybrids, the underlying molecular mechanisms responsible for the enhanced traits remain largely unexplored. This study seeks to address this knowledge gap through sequencing, assembly, and annotation of the transcriptomes of parental and hybrid *B. mori* strains reared in the Philippines. Differentially expressed genes (DEGs) were also identified to find genes associated with desirable traits, including disease resistance, tolerance to extreme temperatures, and high silk production. The identification of these genes holds significant potential, as they may serve as molecular markers for silkworm core germplasm selection and marker-assisted breeding, thereby increasing the efficiency of breeding programs aimed at improving strain performance.

## 1. Introduction

The mulberry silkworm, *Bombyx mori* of the Bombycidae family [1], is one of the few fully domesticated insects worldwide [2]. Their cocoons are a source of the silk fabric, known for its luxurious appearance, superior mechanical properties, and biocompatibility [3]. Sericulture, or the practice of rearing silkworms for silk production, has a huge potential to generate livelihood as it requires minimum investment, involves silkworms with a low gestation period, has high employment potential, and is highly profitable [4]. Unfortunately, silk production in the Philippines is only conducted on a small scale, with the country producing only one ton of silk in a year [5]. To meet the local demand for 10 tons of silk [5], there must be an increase in the supply of good-quality cocoons. Different factors, including genetic constitution, can influence economically important properties like growth rate, cocoon size, silk filament quality, and disease resistance [2,6]. Sericulturists and agriculturists typically employ hybridization to produce an F1 generation with improved traits due to a natural phenomenon known as heterosis or hybrid vigor [7,8]. Previous studies have shown that silkworm heterosis produces more productive, robust, and easily reared hybrids than their parents [9]. This can result in a significant improvement in silk yield and quality.

Unlike other silk-producing countries, the Philippines has no native silkworms. The country’s silkworm germplasm collections, which are housed in rearing facilities in La Union (Ilocos Region, Region 1), Benguet (Cordillera Administrative Region), and Misamis Oriental (Northern Mindanao, Region X), consist of introduced strains from other silk-producing countries like China, India, and Japan [10]. Because these strains are not native to the Philippines, their ability to produce cocoons may also be impacted as this highly depends on environmental conditions like temperature and relative humidity [6]. This highlights the need for genetic studies on Philippine-reared *B. mori* strains in the country’s germplasm collection.

In rearing facilities across the Philippines, introduced strains maintained through years of backcrossing and inbreeding are used for hybridization. Lat21 and B221, which were both from the Kumamoto prefecture in Japan, are two strains regularly used for producing commercial hybrids in the rearing facility of the Department of Science and Technology-Cordillera Administrative Region (DOST-CAR), La Trinidad, Benguet. Crossing these two strains resulted in hybrids performing better than their parents under local conditions, with improved traits like tolerance to extreme temperatures, disease resistance, hatching percentage, fecundity, cocoon filament length, and cocoon yield [11]. For these reasons, local sericulturists in CAR prefer to regularly culture and distribute these hybrids from Philippine-reared Lat21 and B221.

Since hybrids inherit their genetic material from their parents, no new genes are produced. This suggests that the observed hybrid vigor is attributed to changes in gene expression or gene modifications [12]. To understand the basis for these changes, transcriptomics, a technique commonly used for studying complex traits such as silk production, can be utilized [12]. This method provides insight into the functional elements of the genome [13], collectively referred to as the transcriptome [14].

This study aims to investigate the effects of hybridization on the transcriptome of the Philippine-reared parental strains Lat21 and B221, sourced from the DOST-CAR Sericulture Facility in La Trinidad, Benguet, along with their corresponding hybrid lines, NC144 (Lat21♀ × B221♂) and CN144 (B221♀ × Lat21♂). Given that silk yield and other economically important traits are inherently complex, transcriptome analysis will provide insights into the genes, biological pathways, and molecular mechanisms contributing to the observed hybrid vigor. By examining the transcriptomes, this study will elucidate how hybridization influences gene expression and the overall metabolic pathways in these silkworm strains.

The outcomes of this research are expected to yield critical insights into the genetic basis of heterosis within Philippine *B. mori* strains. Furthermore, this knowledge will contribute to developing strategies that utilize molecular markers, thereby promoting sustainable sericulture practices. Such advancements will not only enhance the productivity of silkworm breeding programs but will also ensure the economic viability of the sericulture industry in the Philippines.

## 2. Materials and Methods

### 2.1. Sample Collection and Preservation

The Philippine-reared parental strains Lat21 and B221 from the DOST-CAR Sericulture Facility silkworm germplasm in La Trinidad, Benguet, were reciprocally crossed to produce two hybrid lines: NC144 (Lat21♀ × B221♂) and CN144 (B221♀ × Lat21♂). Rearing of the parental strains was conducted first from November to December 2021 and larvae that were not collected for RNA-seq were subsequently hybridized. Eggs resulting from the cross between these two parental strains were then reared in the succeeding cycle, from June to July 2022, leading to the production of the F1 hybrids NC144 and CN144.

Silkworm samples for both parental and hybrid strains were reared in DOST-CAR, La Trinidad, Benguet (Coordinates: 16.45846597579819, 120.58826862840532). A relative humidity of 80 to 85% and a temperature of 26 to 28 °C were maintained from 1st to 3rd instar, and a relative humidity of 75 to 80% and a temperature of 22 to 24 °C from 4th to 5th instar. During the rearing period, the silkworms were fed mulberry leaves three times per day, specifically in the morning, afternoon, and evening.

Three (3) unsexed individuals from each strain were collected on the third day of the fifth instar, after the first round of feeding for the day. These were then transported to the National Institute of Molecular Biology and Biotechnology, Ma. Regidor Street, University of the Philippines Diliman, Quezon City, where they were preserved via flash-freezing in liquid nitrogen and stored at −80 °C.

### 2.2. RNA Extraction, Library Preparation, and Sequencing

Total RNA was extracted from whole-silkworm samples using TRIzol reagent (Invitrogen, Life Technologies, Carlsbad, CA, USA). Three individual silkworm larvae per strain were carefully dissected to remove plant and waste materials from their digestive system and then washed with cold 1X PBS. This was performed on ice to maintain the integrity of the samples. Homogenization of the silkworm tissue was performed immediately afterward using a mortar, pestle, and liquid nitrogen.

The resulting RNA extracts were treated with Turbo DNAse (Ambion, Life Technologies, Carlsbad, CA, USA) and purified using the RNA Clean and Concentrator kit (Zymo Research, Irvine, CA, USA). The quality and purity of the total RNA extracts were assessed via agarose gel electrophoresis (AGE) and a Qubit RNA BR Assay (Thermo Fisher Scientific, Waltham, MA, USA). Extracts were also analyzed using the Agilent 2200 TapeStation (Agilent Technologies, Santa Clara, CA, USA) to determine the RNA Integrity Number (RIN). RNA samples with RIN ≥ 6.9 were used for library preparation and sequencing.

The TruSeq Stranded RNA kit (Illumina, San Diego, CA, USA) was used for library preparation, with 80 to 189 ng of RNA as the input. A total of 12 libraries were prepared (4 strains × 3 bioreplicates), which were then diluted, pooled, spiked with 1% PhiX, and denatured. The resulting denatured library was loaded for sequencing in the Novaseq 6000 Sequencing System (Illumina, San Diego, CA, USA) at the Philippine Genome Center, University of the Philippines.

### 2.3. Bioinformatics Analysis

Sequencing reads were subjected to quality assessment and trimming using FastQC (v0.12.1) [15] and fastp (v0.23.4) [16], and only good-quality reads (Phred score ≥ 30) were retained. This was followed by alignment to the *B. mori* reference genome (NCBI GCF_030269925.1) using the spliced aligner STAR (v2.7.11) [17]. Once alignment was completed, reference-based assembly using StringTie (v2.2.3) [18] was performed, alongside de novo assembly using Trinity (v2.15.1) [19]. Evaluation of the resulting assemblies was then performed using BUSCO (v5.6.1) [20] and Trinitystats.pl (Trinity v2.15.1), after which the quantification of gene expression was performed using featureCounts (Subread Package v2.0.6) [21].

Differential gene expression analysis was conducted using the DESeq2 package (v1.44.0) [22] in R Studio (v2024.04.2 + 764) [23]. Analysis was focused on the identification of upregulated and downregulated genes in two contexts: first, between the hybrids and their parental strains, and second, among the two hybrids themselves. This was performed to determine the genes that possibly contribute to differences in economically important traits such as disease resistance, tolerance to extreme temperatures, and high silk production. Furthermore, comparing the DEGs between the hybrids allows for the identification of genes associated with these economically important traits. DEGs were visualized using heat maps and volcano plots in R Studio and then annotated using BLAST and the NCBI nr database. Additionally, the DEGs were submitted to g:Profiler (version e111_eg58_p18) [24] for gene ontology (GO) enrichment analysis.

### 2.4. Quantitative Real-Time PCR Validation of Gene Expression

Representative genes were selected for qPCR validation from the list of differentially expressed genes. These were selected from the top DEGs based on two criteria: their adjusted *p*-values (*p*adj), with the threshold set at 0.1, and on potential relevance or importance to the silk industry (e.g., disease resistance, tolerance to extreme temperatures, and high silk production) from the existing literature. Primers targeting these genes were then designed using Primer-BLAST (NCBI) and PrimerQuest (Integrated DNA Technologies, Inc., Coralville, IA, USA), based on their corresponding sequences derived from the transcriptome assembly.

Aliquots of RNA used for library preparation and sequencing were normalized to 1000 ng and synthesized into cDNA using the ProtoScript^®^ II First Strand cDNA Synthesis Kit (New England Biolabs, Ipswich, MA, USA). Synthesized cDNA samples were then used as templates for qPCR validation.

To test the efficiency of these primers, standard curves were first generated using pooled cDNAs normalized to 20 ng/μL, which were then used to prepare a ten-fold dilution series; these dilutions ranged from 40 pg to 40 ng. For succeeding qPCR assays for gene expression analysis, 40 ng of cDNA was used.

Each of the 10 μL qPCR reactions contained 5 μL of 2X SsoAdvanced™ Universal SYBR^®^ Green Supermix (Bio-Rad, San Francisco, CA, USA), 0.25 μM each of forward and reverse primer, 2 μL of cDNA, and 2.5 μL of nuclease-free water (NFW). Using the Bio-Rad CFX Opus Real-Time PCR System, these reactions were then subjected to the following conditions: 50 °C for 10 min, 95 °C for 5 min, and 40 cycles of 95 °C for 10 s and 60 °C for 30 s, followed by a melt curve assay from 95 °C to 65 °C in 0.5 °C stepwise increments.

The relative expression level of each gene was calculated using the Pfaffl method, which uses the expression level of the reference gene, in this case, rp49, as a normalization factor for expression data and considers the efficiencies of the primers used [25]. These results were then graphed using GraphPad Prism 10, which was also used for statistical analysis. One-way ANOVA was conducted to compare the DEGs between the parents (Lat21 and B221) and hybrids (NC144 and CN144), followed by Tukey’s multiple comparison test to determine significance between every sample group (*p* = 0.05). Meanwhile, the DEGs between the two hybrids were compared using the unpaired *t*-test.

## 3. Results and Discussion

### 3.1. RNA Sequencing and Pre-Processing of Raw Reads

Total RNA samples with RIN ≥ 6.9 were used for the preparation of libraries using the TruSeq Stranded RNA kit (Illumina, San Diego, CA, USA). These libraries had sizes ranging from 283 to 314 bp based on the Agilent TapeStation results (Figure S1 and Table S1). The sequencing run on the Novaseq 6000 Sequencing System used the standard workflow, with 300 cycles for the 450 pM library containing a 1% PhiX spike-in. This yielded 301.1 Gb reads, 92.2% of which passed the Q30 filter, and had an 80.52% cluster passing filter based on the Novaseq 6000’s machine output.

A closer inspection of the statistics for the raw demultiplexed reads (Table S2) using fastp showed that there were enough reads coming from each replicate to be used for assembly, with the number of reads ranging from 127 to 210 million prior to filtering. Based on FastQC results, a portion of these reads fell below the Q30 threshold and were excluded from the analysis. Clean reads were obtained by trimming and filtering using fastp, which also included the trimming of adapters, poly-G, and poly-X tails. After trimming, 85–95% of raw reads (115–200 M reads) passed the filters, allowing for downstream analysis.

### 3.2. Bioinformatics Analysis

#### 3.2.1. Read Mapping to the Reference Genome

The reference genome for *Bombyx mori* (NCBI GCF_030269925.1), along with its corresponding GTF file, were downloaded from NCBI, https://www.ncbi.nlm.nih.gov/datasets/genome/GCF_030269925.1/ (accessed on: 11 October 2023), and used to prepare a genome index for mapping using the splice-aware aligner STAR. Two-pass mapping was conducted to allow more spliced reads to map novel junctions [17]. It collected the junctions detected during the first-pass mapping and included them as “annotated” junctions during the second-pass mapping. The different STAR parameters were also optimized to yield the best mapping statistics. Parameters that were adjusted included “--outFilterMismatchNmax”, to allow for more mismatches during mapping, and “--outFilterMultimapNmax”, so that reads with multiple alignments were considered unmapped [26]. The resulting statistics for the mapping conducted using the optimized parameters (“--outFilterMismatchNmax 15” and “--outFilterMultimapNmax 40”) are presented in Table S3.

Table S3 shows that 8.09–14.5% of the reads do not map to the reference genome. However, of the total number of reads successfully mapped to the reference, 38.55–46.88% were uniquely mapped, meaning that multimappers accounted for almost half of the total mapped reads. These multimappers were included in the downstream analysis to avoid bias against genomic features with repetitive sequences or high sequence similarity, which can lead to underrepresentation [27].

#### 3.2.2. Transcriptome Assembly and Evaluation

The resulting mapped reads were then merged and sorted using SAMtools. After sorting, the mapped reads were used for reference-based assembly using StringTie. In addition, de novo assembly using Trinity was also conducted.

Both reference-based and de novo assemblies were then assessed using the Benchmarking Universal Single-Copy Orthologue (BUSCO) tool and Trinity statistics measures (trinitystats.pl, align_and_estimate_abundance.pl, abundance_estimates_to_matrix.pl, and contig_ExN50_statistic.pl). BUSCO measures the completeness and redundancy of an assembly based on how many single-copy orthologs or “universal genes” from their corresponding taxonomic group are present [20]. The general rule of thumb for transcriptome assemblies is that they must have a BUSCO completeness of at least 80% to be considered good quality [28].

Before conducting a BUSCO assessment, it is recommended that isoforms be filtered from the assembly; otherwise, these isoforms are counted as duplicated BUSCOs [20]. One of the ways to achieve this is by including only the longest isoform for each transcript in the analysis. Since it is recommended to include all isoforms in other downstream analyses, a separate assembly with only the longest isoforms was created specifically to check the completeness based on BUSCO. This was performed using a Python (ver. 3.7.12) script [29] for the reference-based assembly, while the get_longest_isoform_seq_per_trinity_gene.pl script of the Trinity Package was used for the de novo assembly.

As shown in Table 1, both assemblies had BUSCO completeness scores greater than 80%. However, more complete BUSCOs were identified in the reference-based assembly from StringTie. Of these complete BUSCOs, the majority were also single copies instead of duplicates, suggesting that the filtering of isoforms was successful.

Aside from BUSCO, other contig statistics of the two assemblies were also compared (Table 1). These results show that de novo assembly yielded more transcripts; however, the N50 of the reference-based assembly was higher at 4669 compared to 1220 for the de novo assembly. The N50 value refers to the sequence length wherein half of the contigs in the assembly are at least this length [28]. In most cases, the length of a transcript does not correlate to its expression level. Because of this, another metric for assessing the assembly, known as ExN50, was used.

The ExN50 statistic is more appropriate for transcriptome assemblies since it only considers the most highly expressed genes representing x% of the total normalized expression data [28]. This prevents biases that may arise due to short, lowly expressed transcripts and long isoforms from highly expressed transcripts. One of the most crucial ExN50 values for assessing transcriptome assemblies is the E90N50 or the N50 value based on the transcripts, representing 90% of the expression data [30]. For this metric, the reference-based assembly is better than the de novo assembly, with E90N50 values of 3184 and 1701, respectively. The reference-based assembly should be used for downstream analyses since it yielded better results in three of the four metrics assessed (Table 1).

#### 3.2.3. Expression Quantification, Normalization, and Differential Expression Analysis

To determine the gene-level counts per strain based on their corresponding BAM files and the reference annotation file, featureCounts was used. For this, multimappers were counted as fractions for each instance that they were assigned to a feature. This reduces the chances of overestimating the gene-level counts by splitting the total count between each alignment while better representing each RNA biotype [31]. Presented in Table S4 are the results of the featureCounts when multimappers are included in the analysis. As shown, a total of 1.09 B assigned reads can be utilized for downstream analysis using DESeq2.

Differential expression analysis was conducted twice using DESeq2, once to compare the hybrids with their parents and next to compare the two hybrids. Prior to differential expression analysis, DESeq2 was used to normalize the gene-level counts so that changes in expression level, sequencing depth, and RNA composition were accounted for [32]. There are various methods that consider different factors for normalization, such as counts per million (CPM), transcripts per kilobase million (TPM), and reads/fragments per kilobase of exon per million reads/fragments mapped (RPKM/TPKM). All these methods are not recommended if comparisons are to be conducted between samples due to their biological differences [33]; therefore, it was best to use DESeq2’s median of ratios method for this study [32]. This method works by dividing the counts by sample-specific size factors determined by the median ratio of gene counts relative to the geometric mean per gene. Once normalized, read counts are also log2 transformed to give a better range for the comparison of RNA expressions.

Differential gene expression analysis was then conducted in DESeq2 using the normalized and transformed read counts. Again, DGE analysis was performed (1) between the parents and hybrids and (2) between the two hybrids. For the first set, parental strains were set as the reference condition and hybrid strains as the contrast. On the other hand, the comparison between the two hybrids considered CN144 as the reference and NC144 as the contrast.

Changes in gene expression between different conditions were measured as fold change and were checked for significance using the Wald test [22]. Since the Wald test does not account for the multiple testing problem brought about by the numerous steps involved in RNA-seq analysis, the resulting *p*-values must be adjusted. This can be achieved by following the procedure of Benjamini and Hochberg [34], which is already included in the DESeq2 package [22]. Genes whose adjusted *p*-values (*p*adj) were less than 0.1 were considered differentially expressed. With this threshold, the number of differentially expressed genes (DEGs) identified was 1419 when comparing parents and hybrids and 959 when comparing CN144 and NC144.

**Figure 1:**
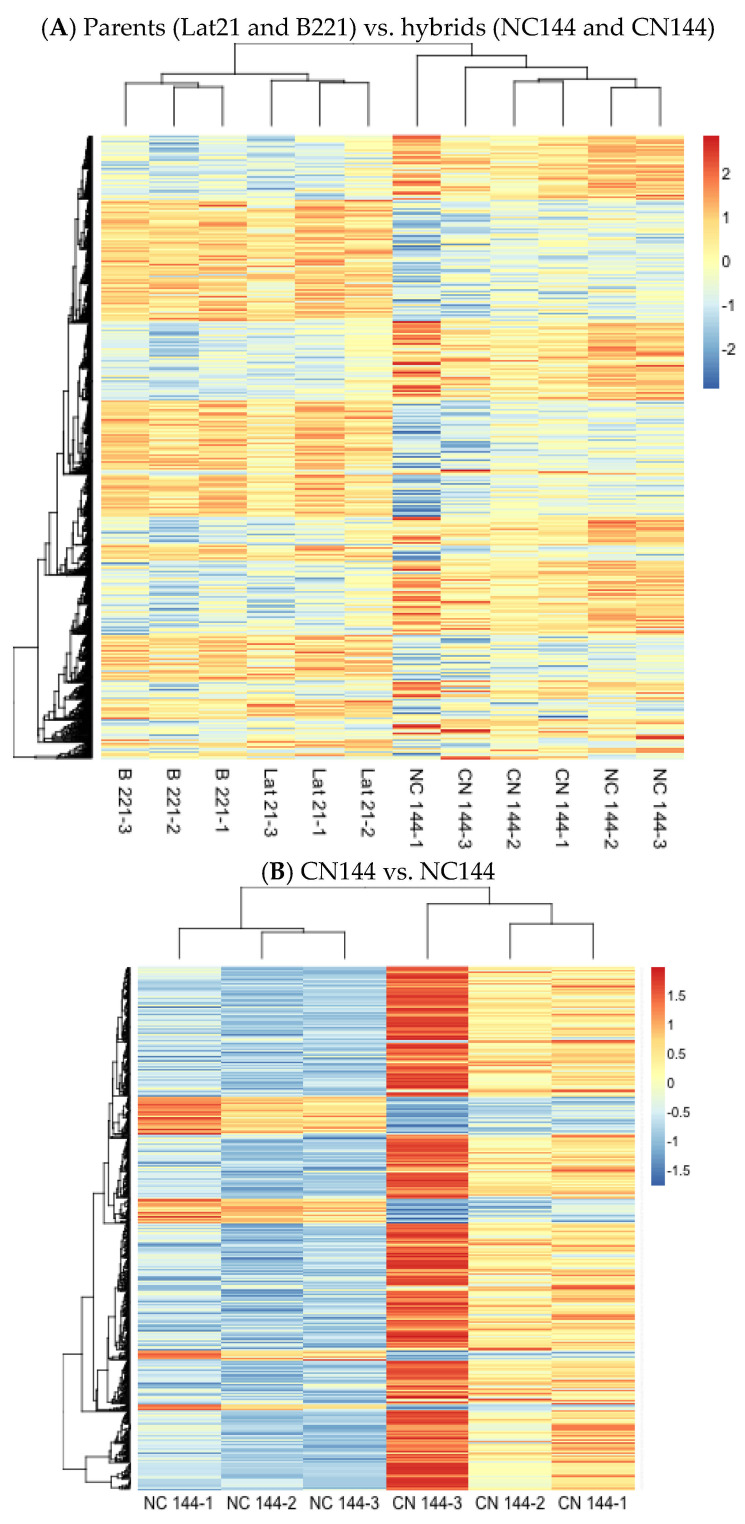
Heat map of the differentially expressed genes in the Philippine-reared *Bombyx mori* parental (Lat21 and B221) and hybrid (NC144 and CN144) strains, with *p*-adj < 0.1. (**A**) DEGs found in parents (Reference) vs. hybrids. (**B**) DEGs found in CN144 (Reference) vs. NC144. In the color scale for normalized gene expression, the more intense red corresponds to higher expression while the more intense blue corresponds to lower expression.

Differentially expressed genes were also visualized using heat maps to provide an overview of the changes in gene expression among the samples. Figure 1A shows two major clusters that can be observed based on gene expression: (1) for the two parental strains Lat21 and B221, and (2) for the two hybrid strains NC144 and CN144. Meanwhile, Figure 1B shows separate clusters for the NC144 and CN144 samples. There also seems to be a relatively equal distribution of upregulated and downregulated genes between the two clusters in Figure 1A, while Figure 1B shows that NC144 samples express more downregulated genes compared to CN144.

Although all samples follow the trend of genes that are upregulated or downregulated depending on the strain, variations are still observed when it comes to the degree of fold change in expression. This is especially evident for NC144-1 in Figure 1A and CN144-3 in Figure 1B, which appear more intense based on the color scale for normalized gene expression, wherein the more intense red corresponds to higher expression and the more intense blue to lower expression. Changes like these can be attributed to biological factors not considered in this study, such as single nucleotide polymorphisms, allelic variations, and genetic mutations within individuals of the same strain [8].

**Figure 2:**
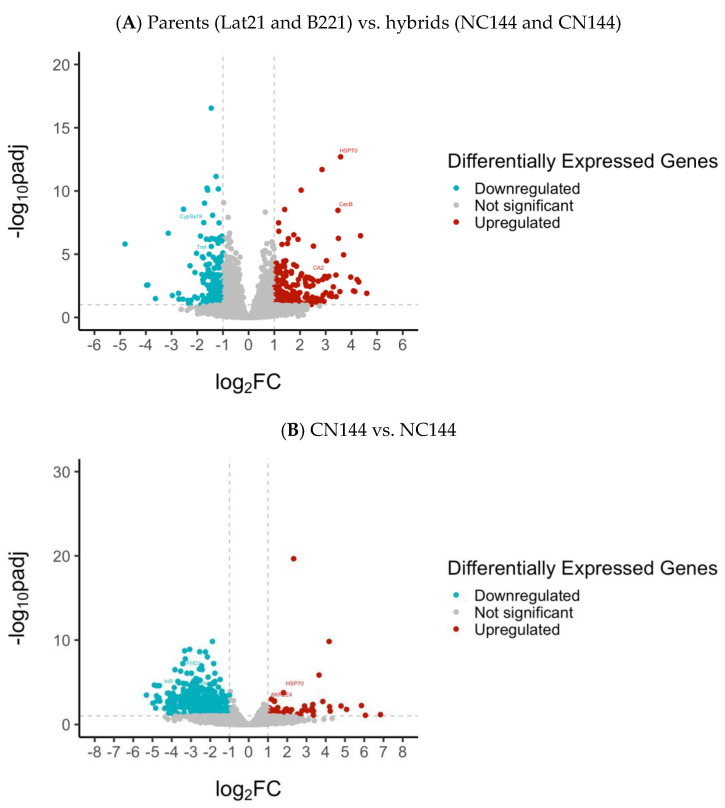
Volcano plot of the differentially expressed genes in the Philippine-reared *Bombyx mori* parental (Lat21 and B221) and hybrid (NC144 and CN144) strains. Red points: DEGs with *p*adj < 0.1 and log2FoldChange > 1; blue points: DEGs with *p*adj < 0.1 and log2FoldChange < 1; and gray points: *p*adj > 0.1 and |log2FoldChange| < 1. Dotted lines indicate the threshold for the log2FoldChange and the *p*adj values. Points corresponding to DEGs selected for qPCR validation are labeled.

From the DEGs identified in the comparison between parents and hybrids, and between the two hybrids, respectively, only those that passed the filter |log2FoldChange| > 1 were considered for downstream analysis, including annotation and gene ontology enrichment analysis. The filter used means that only the genes with a fold change value greater than 2 were considered differentially expressed, which follows the typical cut-off of 2 or 1.5 for fold change analysis [35]. Volcano plots (Figure 2) were used to visualize the distribution of genes with respect to their *p*adj and fold change values. From the numerous genes that were analyzed, many had fold change values between −1 and 1 or *p*adj values greater than 0.1 (gray points). However, many genes were still able to pass both filters (blue and red points). Points corresponding to the DEGs selected for qPCR validation are labeled. A summary of the primers designed for qPCR use, along with one housekeeping gene from the literature [36], is presented in Table 2.

The total number of DEGs that passed the filter set at *p*adj < 0.1 and |log2FoldChange| > 1 was 384 for the parents vs. hybrids (202 upregulated and 182 downregulated in hybrids), and 819 genes for CN144 vs. NC144 (66 upregulated and 753 downregulated in NC144).

#### 3.2.4. Annotation and Gene Ontology (GO) Enrichment Analysis of Differentially Expressed Genes (DEGs)

The corresponding transcript ID and gene names of all the DEGs that passed the filter were determined based on the GTF annotation of the reference used during alignment and the NCBI nr database. A complete list of the annotated DEGs is presented in the Supplementary Files (Tables S5 and S6). Looking at the list of DEGs between the parents and hybrids and between the two hybrids, some genes with potential biological significance are evident. These include genes possibly involved in stress and immune response, silk production, metabolism, and growth and development.

These DEGs were then mapped to their corresponding GO terms and then subjected to GO enrichment analysis using the g:GOSt tool in g:Profiler, https://biit.cs.ut.ee/gprofiler/gost (accessed on 27 November 2024) [24], which is one of the GO-endorsed enrichment tools. This website primarily relies on data from Ensembl and Ensembl Genome, which include the necessary GO annotations for Molecular Function, Biological Process, and Cellular Component [24]. It evaluates the functional enrichment of DEGs by using the cumulative hypergeometric test and performs multiple testing corrections using its own method, called g:Set Counts and Sizes or g:SCS [24].

GO terms that passed the threshold *p*adj < 0.1 were considered significant. Those that were able to pass through the filter are summarized in Table 3 and Table 4, while the DEGs associated with these GO terms can be found in Tables S7 and S8. It is interesting to note that a lot of the upregulated genes in the hybrids compared to their parents were involved in immune or defense responses, especially to bacteria, stress responses, protein maturation, protein folding, and nucleotide metabolism (Table 3). This is in line with the observed increase in the expression of antimicrobial peptides (AMPs) like Cecropin-B, Enbocin, Lebocin, Gloverin, and Attacin 1 in the hybrids [47]. The upregulation of genes involved in protein folding, including the heat shock proteins (HSP70, HSP90, HSP20.8, HSP23.7, and HSP20.4), could contribute to the observed tolerance of the silkworms to cooler temperatures [48].

Interestingly, based on the top GO terms, most of the genes upregulated in the hybrids were inducible in the presence of stressors like bacteria or extreme temperatures [47,48]. This suggests that the observed upregulation of the genes may not just be due to heterosis but also due to the silkworm’s exposure to stressors. It would, therefore, be possible to use the identified genes as diagnostic tools for stress, once validated. To assess their potential use as diagnostic tools, it would be a good idea to again evaluate the expression of these genes in samples reared in ideal conditions, completely free from stressors, and compare them with the currently available data. If the degrees of expression of these genes change, they are likely to be good indicators for stress exposure in the *B. mori* samples and could be used to diagnose stress, even in other strains.

For the DEGs in CN144 vs. NC144 (Table 4), the downregulated genes are of interest since, based on the enriched GO terms, they are primarily involved in regulating cellular and biological processes, signaling, cell communication, and calcium or protein binding. This means that downregulating these genes, including transcription factors like Zinc Finger Proteins and Forkhead box proteins, could have a cascading effect on processes like organ differentiation, growth and development, sexual dimorphism, and responses to infection [49]. One of the important transcription factors that appeared to be downregulated in NC144 is the silk gland factor SGF-3, which is involved in the regulation of the H-fibroin and Sericin 1 expression, two components of the *B. mori* cocoon [50]. This observed downregulation in SGF-3 could, therefore, affect the cocoon produced and the silk fibers from the NC144 silkworms.

A closer look at the individual DEGs that were annotated shows other interesting genes (Tables S5 and S6). This includes genes encoding for cuticular proteins such as CPR39, which is upregulated in the hybrids, or CPR60, CPR103, CPR9, downregulated in NC144 compared to CN144. Higher production of cuticular proteins can be associated with the thickening of the cuticle, which can improve tolerance to low temperature-induced stress [48]. Two sericin genes, namely sericin 1 and sericin 3, were also shown to be differentially expressed. Sericin 1 was upregulated in the hybrids; however, sericin 3 appeared upregulated in CN144 and downregulated in NC144. Sericin 1 and sericin 3 are the main sericin components of the *B. mori* cocoon and are attributed to its rigid structure [51]. In addition, sericin 1 is believed to be involved in cell immune defense and the regulation of protease inhibitors for microbial resistance during the cocoon stage [52]. *Bombyx mori* produces sericin 1 from the first to fifth instar and sericin 3 only on the fifth day of the fifth instar [51]. This is interesting to note since the samples included in the study were only on their third day of the fifth instar but were observed to express sericin 3. Although sericin is responsible for holding the fibroin fibers together, it is typically considered a waste material in silk production [51].

From the complete list of filtered DEGs, several were selected for validation through qPCR based on their *p*adj values and corresponding potential biological significance based on the literature. These are summarized in Table 5 and Table 6 for the parents (Lat21 and B221) vs. hybrids (NC144 and CN144) and the CN144 vs. NC144 datasets, respectively.

Although DEGs have been identified, the potential contribution to complex, quantitative traits like cocoon shell weight, filament length, and fecundity can be further supported by other data besides those generated by transcriptomics. This is especially true with the limited data available from the rearing facilities on the quantitative traits and the likely effect of varying conditions during rearing. One way to circumvent these issues is to determine the silkworm’s genotype through quantitative trait loci (QTL) mapping, which may vary depending on the conditions under which RNA is collected. QTL mapping could help identify molecular markers linked to QTLs influencing select traits of interest, given that a large number of samples are used and that the trait being assessed varies between the two parents [53].

Another critical consideration for transcriptomics studies is that changes in gene expression may not show a corresponding change in phenotype. Numerous genes contribute to the manifestation of phenotypes, as can be identified by QTL mapping. Because of this, it is possible for a change in the expression of one gene to be masked by another gene, such that no phenotypic change is observed. In addition, genes may have different isoforms, some of which are not biologically or functionally relevant [54]; therefore, changes in their expression will not have any effect on phenotype. Lastly, transcriptomics only looks at the mRNA level, so there can still be RNA modifications or regulatory mechanisms that prevent changes in phenotype [55].

### 3.3. Quantitative Real-Time PCR Validation of Gene Expression

Quantitative real-time PCR was performed using cDNA samples from the two parental strains, Lat21 and B22, and the two hybrid strains, NC144 and CN144. Initial qPCR runs were conducted to test the efficiency of all primer sets for DEG validation. This assay used varying concentrations of pooled cDNA samples, from 0.04 to 40 ng, to establish a standard curve. The resulting amplification efficiencies are presented in Table 2.

Ideally, PCR efficiency should fall within 90 to 110% for quantitative methods, but a more flexible range of 75 to 110% is also acceptable [56]. Most of the primers, except those targeting *CA2*, *Tret*, *EFHD1*, and *InR*, satisfy this standard (Table 1). The other primers that fell beyond this range had efficiencies of 111.1 to 123.0%. Since these values are not too far off from the acceptable range of efficiencies, they were still used for the differential expression using qPCR.

Quantification of gene expression via qPCR was conducted with the selected primers, and results were then analyzed using the Pfaffl method. This method, unlike the alternative ΔΔCt method, considers the specific primer efficiencies for both the housekeeping and target gene [25]. Gene expression ratios calculated using the Pfaffl method used the average of all Ct values of the control group as the calibrator or reference Ct. This was performed so that both parental strains would be considered, unlike when the highest Ct value from the control group is used and only one of the two parents is represented.

**Figure 3:**
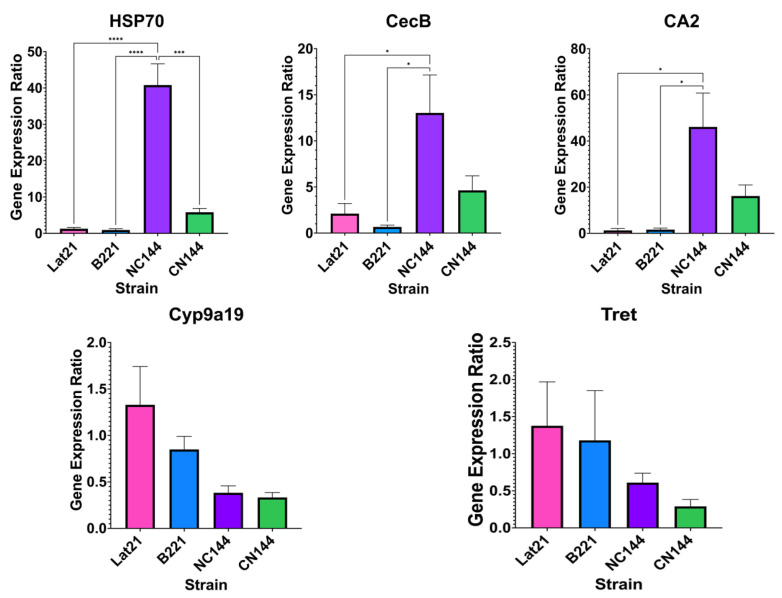
Relative expression of the differentially expressed genes *HSP70* (heat shock protein 70), *CecB* (cecropin-B-like), *CA2* (carbonic anhydrase 2), *Cyp9a19* (cytochrome P450), and *Tret* (facilitated trehalose transporter Tret1-2 homolog isoform X2, X3, X1) in the Philippine-reared *Bombyx mori* parental (Lat21 and B221) and hybrid (NC144 and CN144) strains, based on the results of quantitative real-time PCR. Relative expressions were normalized using the household gene *rp49*. Of these five genes, *HSP70*, *CecB*, and *CA2* were expected to be upregulated in the hybrids, while *Cyp9a19* and *Tret* were expected to be downregulated based on bioinformatics analysis. Asterisks indicate statistical significance based on Tukey’s multiple comparison test, with the number of asterisks corresponding to *—*p* ≤ 0.05, ***—*p* ≤ 0.001, and ****—*p* ≤ 0.0001.

**Figure 4:**
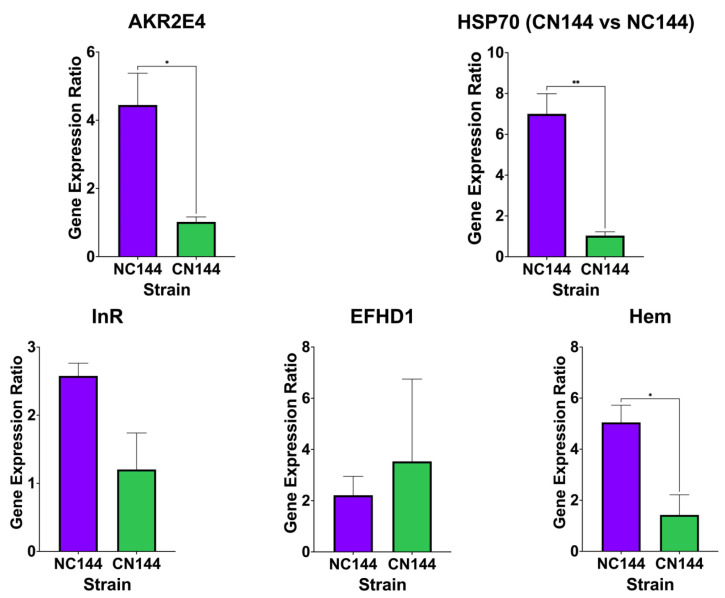
Relative expression of the differentially expressed genes *HSP70* (heat shock protein 70), *AKR2E4* (aldo-keto reductase AKR2E4-like), *InR* (insulin receptor), *EFHD1* (EF-hand domain-containing protein 1), and *Hem* (hemocytin) in the Philippine-reared *Bombyx mori* hybrid strains NC144 and CN144, based on the results of quantitative real-time PCR. Relative expressions were normalized using the household gene *rp49*. Of these five genes, HSP70 and AKR2E4 were expected to be upregulated in NC144 while *InR*, *EFHD1*, and *Hem* were expected to be downregulated based on bioinformatics analysis. Asterisks indicate statistical significance based on Tukey’s multiple comparison test, with the number of asterisks corresponding to *—*p* ≤ 0.05 and **—*p* ≤ 0.01.

Figure 3 shows the relative expression of the DEGs in the parents vs. hybrids. Based on the results of the bioinformatics analysis, *HSP70*, *CecB*, and *CA2* were expected to be upregulated in the hybrids, while *Cyp9a19* and *Tret* were expected to be downregulated. It can be seen in Figure 4 that the expected changes in expression were followed for the gene expression ratios determined using qPCR, such that upregulated genes had higher gene expression ratios in the hybrids, NC144 and CN144, even when looking at them separately. This was also true for downregulated genes with lower gene expression ratios in each of the two hybrids. The statistical analysis indicates that most of the DEGs, particularly *HSP70*, *CecB*, and *CA2*, displayed significant differences only between the parental strains and NC144. This finding suggests that the higher expression levels of NC144 for these genes may mask their expression in CN144, resulting in an overall perception of increased expression when the hybrids are evaluated together. Future studies could improve the clarity of these results by performing differential expression analysis separately between each parent and hybrid. Additionally, the analysis did not reveal significant differences in the expression levels of *Cyp9a19* and *Tret* across either the parental or hybrid strains, which aligns with the expectations based on their low log2 fold change values of −1.75 and −1.45, respectively, as suggested by the bioinformatics analysis.

On the other hand, of the DEGs being validated in CN144 vs. NC144, *HSP70* and *AKR2E4* were expected to be upregulated in NC144 while *InR*, *EFHD1*, and *Hem* were expected to be downregulated. From these, only *HSP70*, *AKR2E4*, and *EFHD1* followed the expected trend in gene expression, while *InR* and *Hem* appeared to be upregulated (Figure 4). Additionally, it was observed that only the expression levels of *HSP70*, *AKR2E4*, and *Hem* were significantly different between the two hybrids based on the unpaired T-test conducted. The algorithm used for mapping and the quantification of multimappers during bioinformatics analysis could have caused the observed deviation and the insignificant difference in relative expression from qPCR data [57]. With multimappers, the over- or underestimation of gene expression is possible due to the sharing of reads between active and inactive copies of a gene [42].

Overall, the qPCR validation of gene expression was successful for seven of the nine genes tested. This means that it is likely that the DEGs identified from the bioinformatics pipeline used to be associated with hybrid vigor.

## 4. Summary and Conclusions

Two parental *Bombyx mori* strains, Lat 21 and B221, and their hybrids, NC144 (Lat21 × B221) and CN144 (B221 × Lat21), were selected for RNA sequencing to determine the molecular basis for the historically observed hybrid vigor. Sequencing yielded a total of 127–210 M reads, with 115–200 M reads passing the Q30 filter of fastp. Good-quality reads were then mapped to the *B. mori* reference genome (GCF_030269925.1) using STAR, which resulted in high mapping rates, with a high percentage of them being multimappers.

Reads that mapped to the reference genome were used for reference-based assembly using StringTie. Alongside this, de novo assembly was conducted using Trinity. Based on the assessment of the two assemblies, the reference-based assembly was better in terms of BUSCO completeness and E90N50 and N50 values. It was, therefore, selected for further downstream analysis.

Using featureCounts, gene-level counts per strain were then determined based on the annotation file of the reference genome. When multimappers were included in the analysis, a total of 623 M to 1.09 B mapped reads were assigned to various genomic regions (18,219 genes). The resulting gene-level counts were used for differential gene expression analysis in DESeq2, which was conducted twice: once to compare parental strains and hybrids and again to compare the two hybrids.

Gene ontology enrichment analysis revealed that upregulated genes in the hybrids were involved in immune or defense response, especially to bacterial exposure, protein maturation, protein folding, and nucleotide metabolism. This could be associated with the observed disease resistance and tolerance of the silkworms to cooler temperatures. Genes potentially contributing to these properties include the upregulated AMPs (Cecropin-B, Enbocin, Lebocin, Gloverin, and Attacin 1) and HSPs (HSP70, HSP90, HSP20.8, HSP23.7, and HSP20.4). Based on the inducible nature of the upregulated genes in the hybrids, the samples might have been exposed to stressors like bacteria or extreme temperatures before they were collected. This means that the upregulated genes that were identified also have potential use as markers for the diagnosis of stress in *B. mori.*

On the other hand, the downregulated genes in CN144 vs. NC144 are more noteworthy since, based on the enriched GO terms, they are primarily involved in regulating cellular and biological processes, signaling, cell communication, and calcium or protein binding. Genes associated with these terms include transcription factors like Zinc Finger Proteins and Forkhead box proteins that regulate organ differentiation, growth and development, sexual dimorphism, and immune response. Among these transcription factors is SGF-3, which is involved in H-fibroin and Sericin 1 regulation.

Other annotated DEGs of interest include those encoding various cuticular proteins and the two sericin genes, sericin 1 (upregulated in hybrids) and sericin 3 (downregulated in NC144). Cuticular proteins contribute to the thickening of the cuticle, which can result in improved tolerance to low temperature-induced stress. Meanwhile, sericin 1 and 3 are the main sericin components of the *B. mori* cocoon that provide its rigid structure, and these are typically removed during the degumming process in silk extraction.

Nine DEGs that passed the *p*adj < 0.1 threshold were selected for qPCR validation based on their corresponding biological significance. For the parents vs. hybrids comparison, these included *HSP70*, *CecB*, and *CA2*, which were predicted to be upregulated in the hybrids, and *Cyp9a19* and *Tret*, which were predicted to be downregulated. On the other hand, the DEGs validated in the CN144 vs. NC144 comparison were *HSP70* and *AKR2E4* (upregulated in NC144) and *InR*, *EFHD1*, and *Hem* (downregulated in NC144). Primers designed to target these genes were able to successfully amplify them through qPCR. Using the Pfaffl method, gene expression ratios were calculated from the Ct values determined by qPCR. Except for *InR* and *Hem*, the rest of the genes followed the predicted change in expression, thereby validating the results of the bioinformatics analysis.

Hybridization is one of the ways through which sericulturists can achieve the desired economically important traits and improve the silk output. Transcriptomic studies like this can help them better understand the molecular basis for the observed heterosis or hybrid vigor, especially for complex traits like disease resistance and tolerance to extreme temperatures. The genes identified as associated with these desired traits can be used as molecular markers for the identification and selection of strains to be used for marker-assisted breeding, as well as for diagnostic tools for stress. For example, HSP70 and Cecropin B-like, identified as differentially expressed, can be associated with tolerance to extreme temperatures and disease resistance caused by bacteria, respectively. This is useful for marker-assisted breeding and the identification of samples exposed to specific stressors (e.g., bacteria and extreme temperature). Overall, this study provides better support for evidence-based improvements to be implemented in local silkworm breeding programs, which will help Filipino sericulturists meet the increasing global and local demand for silk.

Moving forward, however, there are still a lot of things that could be achieved to improve the study further. The first of these is improving the data collection and consistency in rearing conditions at the rearing facilities, such that the identification of heritable traits and the genes associated with them will be easier. If these cannot be improved, QTL mapping can be performed as an alternative approach to identifying molecular markers or genotypes that may be associated with the desired traits. With the current data available, more DEGs can be selected for validation to assess if they could be used as markers. Still, in the future, spatial transcriptomics for organs like the silk glands, gut, fat bodies, and Malpighian tubules can also be performed to improve the data collected. The four strains included in this study could even be compared to the same strains reared in other facilities in the Philippines or the parents’ place of origin. Furthermore, studies may be conducted on the mulberry leaves fed to the silkworms, since they could also contribute to the *B. mori* phenotypes observed.

## Figures and Tables

**Table 1:** Summary of the different metrics used to assess the transcriptome assemblies of the Philippine-reared *Bombyx mori* parental (Lat21 and B221) and hybrid (NC144 and CN144) strains resulting from (1) reference-based assembly using StringTie after two-pass mapping with STAR and (2) de novo assembly using Trinity. C: complete, S: single-copy, D: duplicated, F: fragmented, and M: missing.

Assembly	BUSCO (Lepidoptera)	Total Number of Transcripts	N50	E90N50
Reference-based (StringTie)	C: 92.7% [S: 89.4%, D: 3.3%], F: 1.9%, M: 5.4%	70,217	4669	3184
de novo (Trinity)	C: 88.3% [S: 87.4%, D: 0.9%], F: 3.5%, M: 8.2%	406,566	1220	1701

**Table 2:** Primers used for qPCR validation of differentially expressed genes in Philippine-reared *Bombyx mori* parental strains (Lat21 and B221) and corresponding hybrids (CN144 and NC144).

Target	Putative Function	Primer Name	Sequence (5′->3′) Forward Reverse	Size (bp)	Primer Efficiency
*Heat shock protein 70* (*HSP70*)	Involved in adaptation to extreme temperatures [37]	*HSP70*	TAAGGACATCGGCACAGTAATC	137	94.6%
			GAGTGAAGGCCACGTATGAA		
*Cecropin B-like* (*LOC101739536*)	Antimicrobial peptide for humoral response [38]	*CecB*	CACATCAAGTGATCAGTACAGC	211	87.5%
			GCTTTGACGATGCCATCAC		
*Carbonic anhydrase 2* (*LOC101745610*)	Regulates the pH gradient in the silk glands for silk protein assembly [39]	*CA2*	TCTTCGATGTTCTGGATTCGAT	161	111.1%
			ACAATGCTCGGGCTTAAGATT		
*Cytochrome P450* (*Cyp9a19*)	Metabolism of hormones, plant secondary metabolites, and insecticides [40]	*Cyp9a19*	CACCGAGGAGAACAACAGATT	183	85.9%
			TCCTTCATGGTGCCCATTATC		
*Facilitated trehalose transporter Tret1-2 homolog isoform X2*, *X3*, *X1* (*LOC101744355*)	Potential function as receptors for virus entry [41]. Involved in host defense [42]	*Tret*	GTGCAGTGGGTCGTGGTAA	214	123.0%
			ACTCTCAATTTTCGGACACCA		
*Aldo-keto reductase AKR2E4-like* (*LOC101735876*)	Ecdysone metabolism and in xenobiotic degradation [43]	*AKR2E4*	GCTCCATCGCCCAGAAATA	152	99.9%
			CTCGTCTGGTGTAAGAGTGAAG		
*EF-hand domain-containing protein 1* (*LOC101743940*)	Involved in resistance to *Bombyx mori* nuclear polyhedrosis virus (BmNPV) infection [44]	*EFHD1*	CAACCTTAACGCCAACAAGTATC	142	111.7%
			GGAATTTCGACGCGGTATCT		
*Hemocytin* (*LOC692743*)	Has a protective role of during *Nosema bombycis* infection [45]	*Hem*	CTTGAGCTTAGGATCGACTGAC	194	88.9%
			CATTAAGCCGTAACGAGTAGGA		
*Insulin Receptor* (*InR*)	Involved in normal growth and development [46]	*InR*	CTTCGTTCGTGGCAACTGGT	208	115.3%
			GACGGACATACTGAGCTCGAC		
*Housekeeping gene: Ribosomal Protein 49*	-	*rp49*	CAGGCGGTTCAAGGGTCAATAC	213	91.6%
			TGCTGGGCTCTTTCCACGA		

**Table 3:** Top 10 gene ontology (GO) terms enriched in upregulated and downregulated genes in the Philippine-reared *Bombyx mori* hybrids (NC144 and CN144) when parental strains (Lat21 and B221) are used as reference, generated using g:Profiler for biological process, cellular component, and molecular function. GO terms listed passed the *p*adj < 0.05 filter.

GO Term ID	Source	GO Description	*p*adj
***Upregulated genes in the hybrids (reference: Parental strains Lat21 and B221)***			
GO:0042742	Biological Process	defense response to bacterium	8.43 × 10^−12^
GO:0009617	Biological Process	response to bacterium	1.40 × 10^−11^
GO:0044183	Molecular Function	protein folding chaperone	2.12 × 10^−11^
GO:0051604	Biological Process	protein maturation	2.41 × 10^−11^
GO:0006457	Biological Process	protein folding	2.93 × 10^−11^
GO:0140662	Molecular Function	ATP-dependent protein folding chaperone	4.01 × 10^−10^
GO:0098542	Biological Process	defense response to other organism	1.02 × 10^−9^
GO:0043207	Biological Process	response to external biotic stimulus	1.72 × 10^−9^
GO:0051707	Biological Process	response to other organism	1.72 × 10^−9^
GO:0044419	Biological Process	biological process involved in interspecies interaction between organisms	1.72 × 10^−9^
GO:0009607	Biological Process	response to biotic stimulus	1.72 × 10^−9^
***Downregulated genes in the hybrids (reference: Parental strains Lat21 and B221)***			
GO:1901565	Biological Process	organonitrogen compound catabolic process	3.20 × 10^−5^
GO:0044282	Biological Process	small molecule catabolic process	1.04 × 10^−4^
GO:1901606	Biological Process	alpha-amino acid catabolic process	1.06 × 10^−4^
GO:0016491	Molecular Function	oxidoreductase activity	1.26 × 10^−4^
GO:0009063	Biological Process	amino acid catabolic process	1.78 × 10^−4^
GO:0170040	Biological Process	proteinogenic amino acid catabolic process	2.23 × 10^−4^
GO:0044248	Biological Process	cellular catabolic process	3.92 × 10^−4^
GO:0170035	Biological Process	L-amino acid catabolic process	4.52 × 10^−4^
GO:0009056	Biological Process	catabolic process	6.34 × 10^−4^
GO:1901575	Biological Process	organic substance catabolic process	7.86 × 10^−4^

**Table 4:** Top 10 gene ontology (GO) terms enriched in upregulated and downregulated genes in the comparison between Philippine-reared *Bombyx mori* hybrids NC144 and CN144, with CN144 as reference, generated using g:Profiler for biological process, cellular component, and molecular function. GO terms listed passed the threshold *p*adj < 0.05.

GO Term ID	Source	GO Description	*p*adj
***Upregulated Genes in NC144 (reference: CN144)***			
GO:0000723	Biological Process	telomere maintenance	6.38 × 10^−3^
GO:0032200	Biological Process	telomere organization	7.33 × 10^−3^
GO:0003678	Molecular Function	DNA helicase activity	7.62 × 10^−3^
***Downregulated Genes in NC144 (reference: CN144)***			
GO:0050794	Biological Process	regulation of cellular process	1.61 × 10^−5^
GO:0050789	Biological Process	regulation of biological process	1.67 × 10^−5^
GO:0065007	Biological Process	biological regulation	6.11 × 10^−5^
GO:0035556	Biological Process	intracellular signal transduction	1.55 × 10^−3^
GO:0005509	Molecular Function	calcium ion binding	3.22 × 10^−3^
GO:0005515	Molecular Function	protein binding	3.45 × 10^−3^
GO:0023052	Biological Process	signaling	9.00 × 10^−3^
GO:0007154	Biological Process	cell communication	9.75 × 10^−3^
GO:0007165	Biological Process	signal transduction	3.03 × 10^−2^
GO:0051171	Biological Process	regulation of nitrogen compound metabolic process	4.12 × 10^−2^
GO:0080090	Biological Process	regulation of primary metabolic process	4.12 × 10^−2^

**Table 5:** List of differentially expressed genes identified in the comparison between Philippine-reared *Bombyx mori* parental (Lat21 and B221) and hybrid (NC144 and CN144) strains, with the parental strains as reference, that have potential importance in the silk industry based on the literature. These DEGs were selected for validation of gene expression using qPCR.

GO Term ID	log2FoldChange	*p*adj	Protein	Associated GO Terms
***Upregulated genes in the hybrids (reference: parental strains Lat21 and B221)***				
HSP70	3.58	2.01 × 10^−13^	Heat shock protein 70	Stress-inducible proteins involved in the adaptation and resistance to thermal and cold stresses of *B. mori* [37]
LOC101739536	3.48	3.43 × 10^−9^	Cecropin-B-like	One of the major antimicrobial peptides induced in the *B. mori* hemolymph as a humoral defense against microorganisms [38]
LOC101745610	3.03	3.28 × 10^−5^	Carbonic anhydrase 2	Involved in regulating and creating the pH gradient in the silk glands. This pH gradient is potentially involved in the assembly of silk proteins and their consequent formation into fibers [39]
***Downregulated genes in the hybrids (reference: parental strains Lat21 and B221)***				
Cyp9a19	−1.75	3.18 × 10^−8^	Cytochrome P450	P450s are involved in the metabolism of hormones, plant secondary metabolites, and insecticides possibly found on mulberry leaves. Cyp9a19 is one of the P450 genes with relatively high expression in fat bodies, silk glands, and malpighian tubules. It is also one of the most responsive P450s to pesticide exposure [40]
LOC101744355	−1.45	2.44 × 10^−6^	Facilitated trehalose transporter Tret1-2 homolog isoform X2, X3, X1	Sugar transporters were constitutively expressed in susceptible *B. mori* and were hypothesized to function as receptors for virus entry [41]. It was observed to be upregulated after the infection of susceptible *B. mori*, suggesting involvement in host defense [42].

**Table 6:** List of differentially expressed genes identified in the comparison between Philippine-reared *Bombyx mori* hybrids NC144 and CN144, with CN144 as the reference, that have potential importance in the silk industry based on the literature. These DEGs were selected for the validation of gene expression using qPCR.

Gene ID	log2FoldChange	*p*adj	Protein	Function
***Upregulated genes in NC144 (reference: CN144)***				
HSP70	1.81	1.77 × 10^−4^	Heat shock protein 70	Stress-inducible proteins involved in adaptation and resistance to thermal and cold stresses of *B. mori* [37].
LOC101735876	1.33	1.66 × 10^−3^	Aldo-keto reductase AKR2E4-like	Based on kinetic and structural studies, it has a potential role in *B. mori* ecdysone metabolism and in xenobiotic degradation, especially of the commonly used pesticide diazinon [43].
***Downregulated genes in NC144 (reference: CN144)***				
LOC101743940	−2.98	8.63 × 10^−7^	EF-hand domain-containing protein 1	It has potential involvement in resistance to *B. mori* nuclear polyhedrosis virus (BmNPV) infection since this was downregulated in the resistant strains after BmNPV infection [43].
LOC692743	−2.18	4.90 × 10^−4^	Hemocytin	RNAi technology showed that the inhibition of hemocytin led to a proliferation of *Nosema bombycis*, a fungi-related unicellular parasite, within the silkworm. This suggests a protective role of hemocytin during *N. bombycis* infection possibly through pro-inflammatory effects like facilitating pathogen adherence, hemocyte agglutination, and melanization [45].
InR	−3.50	5.32 × 10^−4^	Insulin receptor	Using RNAi to knock down the insulin receptor gene in *B. mori* led to growth inhibition and malformation, such as abnormal body color in black [46].

## Data Availability

The RNA-seq dataset generated in this study has been deposited in the National Center for Biotechnology Information (NCBI) Gene Expression Omnibus, and is accessible through GEO Series Accession Number GSE287287.

## Supplementary Materials

The following supporting information can be downloaded at https://www.mdpi.com/article/10.3390/insects16030243/s1, Figure S1: TapeStation gel image of RNAseq sample libraries prepared from the RNA of Philippine-reared Bombyx mori parental (Lat21 and B221) and hybrid (NC144 and CN144) strains using the TruSeq Stranded RNA kit (Illumina). Table S1: Results of the evaluation of libraries prepared from the RNA of Philippine-reared *Bombyx mori* parental (Lat21 and B221) and hybrid (NC144 and CN144) strains using the TruSeq Stranded RNA Library Prep Kit (Illumina); Table S2: Statistics for the raw and pre-processed RNA-seq reads of Philippine-reared *Bombyx mori* parental (Lat21 and B221) and hybrid (NC144 and CN144) strains; Table S3: Results of two-pass mapping via STAR of pre-processed reads of Philippine-reared *Bombyx mori* parental (Lat21 and B221) and hybrid (NC144 and CN144) strains, with GCF_030269925.1 (NCBI) as the reference genome; Table S4: Gene-level counts obtained from featureCounts for reads (including multimappers) of Philippine-reared *Bombyx mori* parental (Lat21 and B221) and hybrid (NC144 and CN144) strains; Table S5: Differentially expressed genes between Philippine-reared *Bombyx mori* parental strains (Lat21 and B221) and their hybrids (NC144 and CN144) that fall within the threshold set at *p*adj < 0.1 and |log2FoldChange| > 1, as determined using DESeq2; Table S6: Differentially expressed genes between the two Philippine-reared *Bombyx mori* hybrids CN144 and NC144 that fall within the threshold set at *p*adj < 0.1 and |log2FoldChange| > 1, as determined using DESeq2; Table S7: DEGs associated with the Top 10 gene ontology (GO) terms enriched in upregulated and downregulated genes in the Philippine-reared *Bombyx mori* hybrids (NC144 and CN144) when parental strains (Lat21 and B221) are used as reference. GO terms were generated using g:Profiler for biological process, cellular component with a *p*adj < 0.05 filter; meanwhile, the cut-off values for the DEGs were *p*adj < 0.1 and |log2FoldChange| > 1; Table S8: DEGs associated with the Top 10 gene ontology (GO) terms enriched in upregulated and downregulated genes in the comparison between Philippine-reared *Bombyx mori* hybrids NC144 and CN144, with CN144 as reference. GO terms were generated using g:Profiler for biological process, cellular component with a *p*adj < 0.05 filter; meanwhile, the cut-off values for the DEGs were *p*adj < 0.1 and |log2FoldChange| > 1.
