# Supplementary material for: Transcriptomic Profiling Provides Insight into the Molecular Basis of Heterosis in Philippine-Reared Bombyx mori Hybrids
Source: Insects. 2025 Feb 26;16(3):243. doi: 10.3390/insects16030243 (PMC11942671; doi:10.3390/insects16030243)
Supplement: Supplementary file 1 [file insects-16-00243-s001.zip › Table S1 - Results of Evaluation of RNA Libraries.pdf]

Table S1. Results of the evaluation of libraries prepared from the RNA of Philippine-reared *Bombyx mori* parental (Lat21 and B221) and hybrid (NC144 and CN144) strains using the TruSeq Stranded RNA Library Prep Kit (Illumina).

Sample #	Sample name	Average library size (bp)	Concentration (ng/ul)	Stock Concentration (nM)
**1**	**Lat21 Biorep 1**	298	12.9	65.59
**2**	**Lat21 Biorep 2**	301	13.1	65.94
**3**	**Lat21 Biorep 3**	297	21.2	108.15
**4**	**B221 Biorep 1**	314	8.17	39.42
**5**	**B221 Biorep 2**	293	6.14	31.75
**6**	**B221 Biorep 3**	300	17.8	89.9
**7**	**NC144 Biorep 1**	283	14.8	79.24
**8**	**NC144 Biorep 2**	308	23.2	114.13
**9**	**NC144 Biorep 3**	303	24.2	121.01
**10**	**CN144 Biorep 1**	292	17.0	88.21
**11**	**CN144 Biorep 2**	296	26.1	133.60
**12**	**CN144 Biorep 3**	284	14.9	79.49
