# Supplementary material for: Transcriptomic Profiling Provides Insight into the Molecular Basis of Heterosis in Philippine-Reared Bombyx mori Hybrids
Source: Insects. 2025 Feb 26;16(3):243. doi: 10.3390/insects16030243 (PMC11942671; doi:10.3390/insects16030243)
Supplement: Supplementary file 1 [file insects-16-00243-s001.zip › Table S2 - Statistics for the raw and pre-processed RNA-seq reads.pdf]

Table S2. Statistics for the raw and pre-processed RNA-seq reads of Philippine-reared *Bombyx mori* parental (Lat21 and B221) and hybrid (NC144 and CN144) strains.

	Before Trimming and Filtering		After Trimming and Filtering		Percent of Reads that passed the filters
	**Mean Length (bp) R1/R2**	**Number of Reads (Millions)**	**Mean Length (bp) R1/R2**	**Number of Reads (Millions)**	
Lat21 Biorep 1	137/138	161.34	137/137	149.10	92.41%
Lat21 Biorep 2	133/134	150.79	133/132	150.79	91.78%
Lat21 Biorep 3	126/127	185.31	126/126	175.24	94.57%
B221 Biorep 1	135/136	154.77	135/135	142.26	91.92%
B221 Biorep 2	130/131	127.37	130/130	115.16	90.41%
B221 Biorep 3	135/136	158.21	135/133	141.64	89.52%
NC144 Biorep 1	136/137	150.42	136/135	127.93	85.05%
NC144 Biorep 2	137/137	148.78	137/136	138.23	92.91%
NC144 Biorep 3	136/138	155.84	136/136	135.66	87.05%
CN144 Biorep 1	135/135	210.11	135/135	200.08	95.23%
CN144 Biorep 2	135/136	152.93	135/134	139.43	91.17%
CN144 Biorep 3	130/130	179.66	129/129	168.96	94.04%
