# Supplementary material for: Transcriptomic Profiling Provides Insight into the Molecular Basis of Heterosis in Philippine-Reared Bombyx mori Hybrids
Source: Insects. 2025 Feb 26;16(3):243. doi: 10.3390/insects16030243 (PMC11942671; doi:10.3390/insects16030243)
Supplement: Supplementary file 1 [file insects-16-00243-s001.zip › Table S3 - Results of two-pass mapping via STAR.pdf]

Table S3. Results of two-pass mapping via STAR of pre-processed reads of Philippine-reared *Bombyx mori* parental (Lat21 and B221) and hybrid (NC144 and CN144) strains, with GCF_030269925.1 (NCBI) as the reference genome.

Sample #	Strain	Percent of Uniquely Mapped Reads (No. of Reads)	Percent of Reads Mapped to Multiple Loci (No. of Reads)	Percent of Reads Mapped to too Many Loci (No. of Reads)	Percent of Other Unmapped Reads (No. of Reads)
1	Lat21 Biorep 1	44.42% (33.1M)	45.87% (34.2M)	0.97% (726K)	8.73% (6.5M)
2	Lat21 Biorep 2	46.43% (32.1M)	44.76% (31.0M)	0.96% (665K)	7.84% (5.4M)
3	Lat21 Biorep 3	38.55% (33.8M)	46.53% (40.8M)	0.41% (361K)	14.50% (12.7M)
4	B221 Biorep 1	46.88% (33.3M)	44.34% (31.5M)	0.69% (488K)	8.09% (5.8M)
5	B221 Biorep 2	42.29% (24.3M)	47.53% (27.4M)	0.61% (352K)	9.57% (5.5M)
6	B221 Biorep 3	39.42% (27.9M)	49.53% (35.1M)	0.96% (680K)	10.09% (7.1M)
7	NC144 Biorep 1	42.46% (27.1M)	48.00% (30.7M)	0.83% (531K)	8.71% (5.5M)
8	NC144 Biorep 2	43.54% (30.1M)	46.96% (32.5M)	0.40% (277K)	9.09% (6.3M)
9	NC144 Biorep 3	44.69% (30.3M)	44.96% (30.5M)	0.86% (582K)	9.49% (6.4M)
10	CN144 Biorep 1	45.70% (45.7M)	46.43% (46.4M)	0.24% (239K)	7.64% (7.6M)
11	CN144 Biorep 2	44.63% (31.1M)	43.30% (30.2M)	1.10% (770K)	10.96% (7.6M)
12	CN144 Biorep 3	43.99% (37.2M)	46.66% (39.4M)	0.49% (418K)	8.86% (7.5M)
