# Supplementary material for: Transcriptomic Profiling Provides Insight into the Molecular Basis of Heterosis in Philippine-Reared Bombyx mori Hybrids
Source: Insects. 2025 Feb 26;16(3):243. doi: 10.3390/insects16030243 (PMC11942671; doi:10.3390/insects16030243)
Supplement: Supplementary file 1 [file insects-16-00243-s001.zip › Table S4 - Gene-level counts obtained from featureCounts .pdf]

Table S4. Gene-level counts obtained from featureCounts for reads (including multimappers) of Philippine-reared *Bombyx mori* parental (Lat21 and B221) and hybrid (NC144 and CN144) strains.

	Assigned	Unassigned	
		Unmapped	No Features
**Lat21 Biorep 1**	981,168,774	15,771,411	519,350,104
**Lat21 Biorep 2**	991,631,575	13,614,859	490,316,933
**Lat21 Biorep 3**	987,363,007	27,900,880	598,264,386
**B221 Biorep 1**	898,929,934	13,725,334	461,157,665
**B221 Biorep 2**	623,807,188	12,729,784	483,199,697
**B221 Biorep 3**	993,012,546	17,297,874	548,499,794
**NC144 Biorep 1**	685,898,426	13,814,809	480,467,537
**NC144 Biorep 2**	1,092,997,767	14,444,343	508,871,178
**NC144 Biorep 3**	909,118,341	15,624,253	439,161,886
**CN144 Biorep 1**	848,196,640	17,046,638	902,086,324
**CN144 Biorep 2**	695,549,571	18,192,431	467,558,516
**CN144 Biorep 3**	752,718,067	17,378,805	613,439,351
