# Supplementary material for: Transcriptomic Profiling Provides Insight into the Molecular Basis of Heterosis in Philippine-Reared Bombyx mori Hybrids
Source: Insects. 2025 Feb 26;16(3):243. doi: 10.3390/insects16030243 (PMC11942671; doi:10.3390/insects16030243)
Supplement: Supplementary file 1 [file insects-16-00243-s001.zip › Table S5 - DEGs in Parental (Reference) vs Hybrid Strains.pdf]

**Table S5.** List of differentially expressed genes between Philippine-reared *Bombyx mori* parental strains (Lat21 and B221) and their hybrids (NC144 and CN144) that fall within the threshold set at padj<0.1 and |log2FoldChange|>1, as determined using DESeq2.

| Gene ID | normalized mean exp | log2 FoldChange | padj | Protein ID | description |
|---|---|---|---|---|---|
| LOC101739498 | 1491.39156459995 | -1.45544219442205 | 2.8410905970186E-17 | XP_037867558.1 | synaptic vesicle glycoprotein 2A |
| HSP70 | 4563.23245649729 | 3.57976611749775 | 2.00810904895693E-13 | XP_012547740.1 | heat shock protein 70 |
| LOC101737350 | 127.868238635579 | 2.85580049222047 | 2.03841643717725E-12 | XP_037872693.1 XP_062529345.1 | mucin-22 |
| LOC101740667 | 841.744020258496 | -1.26924043083819 | 7.15167951865033E-12 | XP_004921754.1 | PREDICTED: *Bombyx mori* monocarboxylate transporter 10 (LOC101740667) |
| LOC101741141 | 173.732377812865 | -1.6178459420177 | 5.94670625298408E-11 | XP_021209237.2 | uncharacterized LOC101741141 |
| LOC101739275 | 533.568583873366 | -1.17934767070207 | 6.94678374776077E-11 | XP_062524181.1 | *Bombyx mori* protein 60A (LOC101739275), transcript variant X2, mRNA |
| LOC101736069 | 844.69428026042 | -1.17581926184331 | 6.94678374776077E-11 | XP_062524628.1 | myosin-2 heavy chain |
| DnaJ-3 | 422.907470534457 | 2.04475362897708 | 8.67098469054237E-11 | NP_001157381.1 | DnaJ homolog shv |
| LOC101742904 | 1328.90503451389 | -1.59527976990137 | 8.67098469054237E-11 | XP_012550199.1 | Zn finger homeodomain 1 |
| LOC101746176 | 2258.25170315698 | -1.71577428078178 | 9.23143867745779E-10 | XP_004922820.1 | lysoplasmalogenase TMEM86B |
| LOC101745781 | 1435.98217196295 | -2.53339006195692 | 2.77189782161716E-09 | XP_004932532.1 | mitochondrial glycine transporter B |
| LOC100101178 | 231.767065831833 | 1.40171446321869 | 2.92399755762559E-09 | NP_001093276.1 | small ribonucleoprotein particle protein SmF |
| LOC101739536 | 39.0964611175189 | 3.47597220457464 | 3.43496746329508E-09 | NP_001037032.2 | cecropin-B-like |
| LOC101745993 | 795.838407487173 | -1.40195330159384 | 8.41339884112732E-09 | XP_062526585.1, XP_012553009.2 | uncharacterized LOC101745993 |
| Cyp9a19 | 643.007509661094 | -1.74605477731189 | 3.17825525220008E-08 | NP_001103404.1 | cytochrome P450 |
| Tudor-SN | 3985.06181459347 | 1.16444037824444 | 3.30623908082404E-08 | XP_062525679.1 | Staphylococcal nuclease domain-containing protein 1 |
| LOC101739179 | 242.648124923251 | -1.1506710440812 | 3.35589675155778E-08 | XP_037873083.1 | uncharacterized LOC101739179 |
| LOC101737531 | 523.437300395557 | 1.17423234484729 | 1.54772136486025E-07 | XP_062525860.1 | valine--tRNA ligase |
| LOC101744658 | 1561.31965476653 | -3.12710353360826 | 2.20066357768571E-07 | XP_004928152.1 | uncharacterized LOC101744658 |
| LOC101746297 | 1184.95150952152 | 1.75812628701919 | 2.95319005049682E-07 | XP_004928340.1 | hypoxia up-regulated protein 1 |
| LOC101739308 | 1800.18216286995 | 4.35376566360034 | 3.50631609220288E-07 | XP_004927140.1 | trypsin alpha-3 |
| LOC101745130 | 372.839507117474 | -1.0367098447738 | 3.68094206110646E-07 | XP_037876306.1 | putative phospholipase B-like 2 |
| LOC101744777 | 139.65403069828 | -1.87292355713279 | 3.70360800905535E-07 | XP_012546139.3 | uncharacterized LOC101744777 |

| | | | | | | |
|---|---|---|---|---|---|---|
| Xdh1 | 4159.81059680068 | -1.12407719822368 | 4.94892511965508E-07 | NP_001037325.1 | xanthine dehydrogenase rosy |
| LOC101738819 | 721.50844660415 | -1.42961158048039 | 5.63537086614685E-07 | XP_004932233.1 XP_021207671.1 XP_062525766.1 XP_062525768.1 XP_062525767.1 XP_062525765.1 XP_012550935.1 | glutathione hydrolase 1 proenzyme isoform X4, glutathione hydrolase 1 proenzyme isoform X2, glutathione hydrolase 1 proenzyme isoform X2, glutathione hydrolase 1 proenzyme isoform X3, glutathione hydrolase 1 proenzyme isoform X2, glutathione hydrolase 1 proenzyme isoform X2, glutathione hydrolase 1 proenzyme isoform X1 |
| Enb2 | 89.4468778448216 | 3.4935578860381 | 5.63537086614685E-07 | NP_001091844.1 | antibacterial peptide enbocin 2 |
| LOC733070 | 1746.15292853436 | 1.54790579711783 | 5.86102698827627E-07 | NP_001037632.1 | Sec61 translocon subunit beta |
| LOC101745494 | 1538.48511437988 | -1.6439147146496 | 6.41357115897963E-07 | XP_062524620.1, XP_062524621.1, XP_062524622.1, XP_062524619.1 | uncharacterized protein LOC101745494 isoform X1, phosphatidate phosphatase LPIN3 isoform X2, phosphatidate phosphatase LPIN2 isoform X3, uncharacterized protein LOC101745494 isoform X1 |
| LOC101746462 | 791.364419790475 | -1.30404451747431 | 6.41357115897963E-07 | XP_004924250.1 | transcription factor sem-2 |
| Hsp23.7 | 109.657644899004 | 1.9205654483646 | 6.71995744447293E-07 | NP_001036942.1 | heat shock protein hsp23.7 |
| LOC101744595 | 417.639556572696 | -1.51458292859631 | 6.92290184402983E-07 | XP_004922188.1, XP_062526893.1 | serine protease snake |
| LOC101738757 | 691.747089891893 | -1.23160197253786 | 7.86869611802475E-07 | XP_062524760.1 XP_012549160.2 XP_062524762.1 XP_062524761.1 XP_062524759.1 | solute carrier family 12 member kcc isoform X3 solute carrier family 12 member kcc isoform X1 solute carrier family 12 member kcc isoform X5 solute carrier family 12 member kcc isoform X4 solute carrier family 12 member kcc isoform X2 |
| LOC134199358 | 236.587913657269 | -1.0429782654424 | 8.77908471613942E-07 | XP_062526063.1 | uncharacterized LOC134199358 |
| Nep-L | 1105.9253577944 | -1.18518141119831 | 1.36884929622889E-06 | XP_062528977.1, XP_037872516.1, NP_001036959.1, XP_012548893.1, XP_012548894.1, XP_062528926.1, XP_062529005.1 | neutral endopeptidase 24.11 isoform X5 neutral endopeptidase 24.11 isoform X4 neutral endopeptidase 24.11 precursor neutral endopeptidase 24.11 isoform X1 neutral endopeptidase 24.11 isoform X2 neutral endopeptidase 24.11 isoform X3 neutral endopeptidase 24.11 isoform X6 |
| LOC101745692 | 1487.96627447686 | 1.51064826676137 | 1.44831237546078E-06 | XP_004932411.1 | Protein disulfide-isomerase A6 homolog CaBP1 |

| | | | | | |
|---|---|---|---|---|---|
| LOC101739975 | 405.859029965568 | -4.80947599953337 | 1.5756140921033E-06 | XP_004929869.1 | sodium/potassium/calcium exchanger 4 |
| LOC101739156 | 4305.24031083657 | 1.2982135906548 | 1.69697261304206E-06 | XP_012552876.1, XP_012552877.1, NP_001268822.1 | neutral alpha-glucosidase AB-like isoform X1, neutral alpha-glucosidase AB-like isoform X2, glucosidase 2 subunit alpha precursor |
| LOC101743809 | 154.123491726347 | 2.52152720003657 | 2.31905059560411E-06 | XP_037877132.1 | vanin-like protein 1 |
| LOC101744355 | 124.975028859061 | -1.4512850466453 | 2.44011810801587E-06 | XP_021202079.2, XP_062528207.1, XP_037871263.1 | facilitated trehalose transporter Tret1-2 homolog isoform X2, facilitated trehalose transporter Tret1-2 homolog isoform X3, facilitated trehalose transporter Tret1-2 homolog isoform X1 |
| LOC101741694 | 147.474241037001 | -1.00132114021497 | 7.93852352401125E-06 | XP_004933383.1, XP_037873666.1 | proton-coupled folate transporter isoform X1 proton-coupled folate transporter isoform X2 |
| ST3 | 861.934537039047 | -2.02811578100262 | 8.51759218210986E-06 | XP_062532363.1, NP_001182631.1 | sugar transporter protein 3 isoform X1 sugar transporter protein 3 |
| LOC101739798 | 612.916886019785 | -1.01062905884099 | 9.59143418145597E-06 | XP_004932150.1 | ubiquitin carboxyl-terminal hydrolase calypso |
| LOC101745938 | 539.615708145143 | -1.53779139387711 | 9.9504156112008E-06 | XP_004922278.1 | probable galactose-1-phosphate uridylyltransferase |
| LOC101745023 | 30.6424448849582 | 3.695794599561 | 1.12643311125724E-05 | XP_004922749.1 | protein D2 |
| LOC101736773 | 97.9703601028025 | -1.05849843228155 | 1.46713390334029E-05 | XP_062524353.1, XP_062524352.1, XP_062524343.1, XP_062524342.1, XP_062524350.1, XP_062524350.1, XP_062524349.1, XP_062524347.1, | dual specificity calcium/calmodulin-dependent 3',5'-cyclic nucleotide phosphodiesterase 1 isoform X8 dual specificity calcium/calmodulin-dependent 3',5'-cyclic nucleotide phosphodiesterase 1 isoform X7 dual specificity calcium/calmodulin-dependent 3',5'-cyclic nucleotide phosphodiesterase 1 isoform X2 dual specificity calcium/calmodulin-dependent 3',5'-cyclic nucleotide phosphodiesterase 1 isoform X1 dual specificity calcium/calmodulin-dependent 3',5'-cyclic nucleotide phosphodiesterase 1 isoform X6 dual specificity calcium/calmodulin-dependent 3',5'-cyclic nucleotide phosphodiesterase 1 isoform X5 dual specificity calcium/calmodulin-dependent 3',5'-cyclic nucleotide phosphodiesterase 1 isoform X4 dual specificity calcium/calmodulin-dependent 3',5'-cyclic nucleotide phosphodiesterase 1 isoform X3 |

| | | | | | |
|---|---|---|---|---|---|
| | | | | XP_062524344.1,<br>XP_062524346.1,<br>XP_062524345.1,<br>XP_062524351.1 | dual specificity calcium/calmodulin-dependent 3',5'-cyclic nucleotide phosphodiesterase 1 isoform<br>dual specificity calcium/calmodulin-dependent 3',5'-cyclic nucleotide phosphodiesterase 1 isoform X3<br>dual specificity calcium/calmodulin-dependent 3',5'-cyclic nucleotide phosphodiesterase 1 isoform X3<br>dual specificity calcium/calmodulin-dependent 3',5'-cyclic nucleotide phosphodiesterase 1 isoform X6 |
| LOC101740405 | 1752.08840234285 | -1.79942657168631 | 1.59993657987903E-05 | XP_037874032.1,<br>XP_004925341.2,<br>XP_062530369.1,<br>XP_062530370.1,<br>XP_062530371.1 | glutamine synthetase 2 cytoplasmic isoform X2<br>glutamine synthetase 2 cytoplasmic isoform X1<br>glutamine synthetase 2 cytoplasmic isoform X1<br>glutamine synthetase 2 cytoplasmic isoform X1<br>glutamine synthetase 2 cytoplasmic isoform X2 |
| GSTe4 | 229.154704085946 | -1.37298824985462 | 1.7529514888031E-05 | NP_001108460.1 | glutathione S-transferase epsilon 4 |
| LOC119630258 | 54612.948365611 | -1.15452844113521 | 1.89799799666892E-05 | | uncharacterized protein LOC119630258 |
| LOC101736376 | 98.4145594660063 | -1.76790684090931 | 1.98507183695909E-05 | XP_004932673.1 | protein takeout |
| LOC101736818 | 5293.58458585307 | -1.27637604978149 | 3.16837874865931E-05 | XP_004931470.1,<br>XP_004931469.1 | purine nucleoside phosphorylase isoform X2<br>purine nucleoside phosphorylase isoform X1 |
| LOC101745610 | 660.593357670806 | 3.02921903984142 | 3.28499407841559E-05 | XP_012545108.1 | carbonic anhydrase 2 |
| LOC733141 | 903.399667249746 | 1.44491028784883 | 3.28499407841559E-05 | NP_001037674.1 | transport protein Sec61 gamma subunit |
| LOC101743498 | 2696.68815059914 | 1.36528464754546 | 3.56550601167185E-05 | NP_001266403.1 | heat shock protein 90 Gp93 precursor |
| LOC101738706 | 594.137345336189 | -1.07634136050119 | 3.73881356275764E-05 | XP_004926514.1 | Uncharacterized protein LOC101738706 |
| LOC101739317 | 707.139164701336 | 1.07998599186293 | 5.03562827035316E-05 | XP_004928703.1 | vesicle-trafficking protein SEC22 |
| LOC101739124 | 349.801771844309 | -1.52816747895773 | 5.95673494294776E-05 | XP_004926517.1 | ceramide phosphoethanolamine synthase |
| LOC101745352 | 559.570406388175 | 1.37238901914802 | 6.43891110400928E-05 | XP_004932946.1 | 10 kDa heat shock protein, mitochondrial |
| LOC101743955 | 665.728826564223 | -1.18855439608415 | 6.50531024414822E-05 | XP_012551662.2,<br>XP_012551663.2,<br>XP_037876515.1,<br>XP_037876516.1 | protein Skeletor, isoforms D/E isoform X1<br>protein Skeletor, isoforms B/C isoform X3<br>protein Skeletor, isoforms B/C isoform X2, uncharacterized protein LOC101743955 isoform X4 |
| LOC100101160 | 1296.63329746276 | 1.75544391090255 | 6.67575304467944E-05 | NP_001040330.1,<br>NP_001093086.1 | translocon-associated protein gamma isoform X1<br>translocon-associated protein gamma isoform X2 |
| LOC692769 | 191.007838175233 | -1.19866223815419 | 6.71968311864669E-05 | NP_001037353.1 | Rhesus blood group-associated glycoprotein Rh50 |

| | | | | | |
|---|---|---|---|---|---|
| LOC101741137 | 1893.0525062456 | -1.65394831075084 | 7.02340331803494E-05 | XP_062528032.1, XP_062528033.1, XP_062528035.1, XP_062528034.1, XP_004922659.1 | Tryptophan 2,3-dioxygenase vermilion isoform X2, Tryptophan 2,3-dioxygenase vermilion isoform X2, Tryptophan 2,3-dioxygenase vermilion isoform X2, Tryptophan 2,3-dioxygenase vermilion isoform X2, Tryptophan 2,3-dioxygenase vermilion isoform X1 |
| LOC101744125 | 470.735034069758 | 1.0284427821966 | 7.07721712368491E-05 | XP_004927640.1 | zinc finger protein 706 |
| LOC101738086 | 74.7281235292577 | -2.28089392118162 | 8.30847455087972E-05 | XP_004926300.1, XP_062531260.1 | low density lipoprotein receptor adapter protein 1 isoform X1, low density lipoprotein receptor adapter protein 1 isoform X2 |
| LOC791084 | 1305.94092648888 | 1.11979956486818 | 8.70041347949438E-05 | NP_001073348.1 | chaperonin containing TCP1 subunit 8 |
| LOC101741721 | 313.56672220661 | 1.24521292227271 | 8.75986244591362E-05 | XP_004927216.1 | zinc transporter 7 |
| LOC101738119 | 696.439028579476 | 1.86051035979902 | 8.94680470261882E-05 | XP_004925129.2 | translocon-associated protein subunit delta |
| LOC100301978 | 2406.37374725689 | 1.39806252590554 | 9.63630887599195E-05 | NP_001154815.1 | putative cold-induced protein |
| LOC732862 | 2003.71934950313 | 1.22727859919463 | 0.000126871425265165 | NP_001040332.1 | signal sequence receptor beta |
| NGR-B3 | 1152.08519805456 | -1.34510354772085 | 0.000126871425265165 | NP_001127734.1 | neuropeptide receptor B3 |
| cts | 284.999071905177 | -1.1512084498198 | 0.000126871425265165 | NP_001243989.1, XP_012551916.2 | cis, cis-muconate transporter protein cis, cis-muconate transporter protein isoform X1 |
| yellow-12 | 126.26649989656 | -1.32154550200346 | 0.000130418142399571 | NP_001266377.1, XP_062529176.1 | protein yellow-like |
| LOC101741707 | 2260.45574318346 | 1.15769313259507 | 0.000151220010384331 | XP_062530856.1, XP_004923957.1 | heat shock protein 60A |
| LOC101737435 | 556.724183534143 | 1.05062750227983 | 0.000151220010384331 | NP_001296552.1 | dnaJ homolog subfamily C member P58IPK |
| LOC101746159 | 87.6409682921067 | -1.57657058056209 | 0.00016107095586024 | XP_004931899.3 | protein lin-28 homolog |
| LOC100134924 | 460.112372985235 | -1.54013072974688 | 0.000228451757161791 | XP_062524219.1 | cadherin-2 |
| LOC101740353 | 670.93112594576 | 1.06887125727921 | 0.000232836137648392 | XP_012550870.1 | protein NDUFAF4 homolog |
| LOC101744776 | 653.603568858416 | 1.66218451298479 | 0.000233083071280774 | XP_004921981.1 | mesencephalic astrocyte-derived neurotrophic factor homolog |
| LOC778464 | 650.499193462847 | 1.46244410175471 | 0.000246854276768355 | NP_001091763.1 | Signal peptidase complex subunit Spase22-23 |
| LOC101742173 | 190.553519851536 | -2.08989368318285 | 0.000279898853816348 | XP_012551044.1 | uncharacterized LOC101742173 |
| LOC101738074 | 1335.20892508722 | 1.1734368049347 | 0.000282110547934604 | XP_062528585.1, XP_037871803.1 | UDP-glucose:glycoprotein glucosyltransferase isoform X1 |
| LOC101741324 | 1004.31769603787 | 1.02118666458277 | 0.000285174614671506 | XP_004933463.1 | BOS complex subunit NCLN |
| LOC692906 | 786.110208990838 | 1.25819022017286 | 0.000300887093803785 | NP_001040214.1 | Signal peptidase complex subunit Spase25 |

| | | | | | |
|---|---|---|---|---|---|
| Kr-h1 | 117.805292450976 | -1.54085955408729 | 0.00030378025808258 | NP_001171332.1,<br>XP_062533059.1,<br>XP_062533060.1,<br>XP_037877642.1 | Kruppel homolog 1,<br>Kruppel homolog 1 isoform X1<br>Kruppel homolog 1 isoform X1<br>Kruppel homolog 1 isoform X1 |
| LOC101735876 | 1621.50969835032 | 1.66222791373639 | 0.00031757960065035 | XP_004926779.1,<br>XP_004926780.1 | aldo-keto reductase AKR2E4-like isoform X1<br>aldo-keto reductase AKR2E4-like isoform X2 |
| Hsp20.8 | 478.962311321187 | 2.06001115565417 | 0.000348331867669713 | NP_001091794.1 | heat shock protein hsp20.8 |
| Cyp9a20 | 1478.75723582722 | -1.62151324545212 | 0.000358977556266406 | NP_001077079.1 | cytochrome P450 9a20 |
| LOC101736622 | 313.879799312561 | 1.01389545603365 | 0.000361899371706106 | XP_004928268.1 | proteasome subunit beta type-2 |
| LOC119629228 | 81.5028157347668 | -1.38619499725587 | 0.000366901440533373 | XP_037870288.1 | gremlin-2 |
| LOC101746090 | 933.016993671698 | -1.55794258221525 | 0.000373343008006485 | XP_004923313.2 | putative glutathione-specific gamma-<br>glutamylcyclotransferase 2 |
| LOC101744652 | 298.409635981156 | -1.82073471930548 | 0.000402276744326421 | XP_037867702.1 | synaptic vesicle glycoprotein 2A |
| LOC101736611 | 443.347018450891 | -1.21035775982385 | 0.000416690101923045 | XP_004923651.2 | GATOR1 complex protein NPRL2 |
| LOC101736428 | 50.8660438669317 | -1.342686299283 | 0.000443952178069819 | | Uncharacterized protein LOC101736428 |
| LP4 | 3713.60961838608 | 3.39803619446165 | 0.000445748488118759 | XP_012550539.1,<br>NP_001095199.1 | low molecular lipoprotein 30K pBmHPC-21 isoform X1<br>low molecular lipoprotein 30K pBmHPC-21 |
| LOC101739481 | 73.1270323618654 | -1.47563766630035 | 0.000445748488118759 | XP_004933047.1 | Lysyl oxidase homolog 3 |
| LOC732968 | 500.712975429036 | 1.05689780038345 | 0.000446573468585823 | NP_001040431.1,<br>XP_037873832.1 | cyclic AMP-regulated protein<br>cyclic AMP-regulated protein isoform X1 |
| LOC101738305 | 42.7928109157169 | -1.10080356975158 | 0.000455196645771538 | XP_012546741.1 | L-dopachrome tautomerase yellow-f2 |
| Desat3 | 3887.32737682468 | 2.03346217537779 | 0.000500637867346491 | NP_001037018.1,<br>XP_012545279.1 | acyl-CoA desaturase, acyl-CoA desaturase isoform X1 |
| LOC101742363 | 966.839922133989 | 1.45247327449295 | 0.00051027311953562 | XP_004930906.1 | clavesin-1 |
| Y-d | 804.722116520334 | -1.21148523915806 | 0.00051027311953562 | NP_001037422.1,<br>XP_062529193.1,<br>XP_062529195.1,<br>XP_012545566.1,<br>XP_062529190.1,<br>XP_062529192.1,<br>XP_062529191.1,<br>XP_062529189.1, | yellow-d<br>yellow-d isoform X1<br>yellow-d isoform X1 yellow-d<br>isoform X1<br>yellow-d isoform X1<br>yellow-d isoform X1<br>yellow-d isoform X1<br>yellow-d isoform X1<br>yellow-d isoform X1 |

| | | | | | |
|---|---|---|---|---|---|
| | | | | XP_062529194.1,<br>XP_062529196.1 | yellow-d isoform X2 |
| LOC101745057 | 320.612562757487 | -1.08953342385147 | 0.000518702655498103 | XP_062531079.1 | uncharacterized protein LOC101745057 |
| LOC101737905 | 214.45683568911 | -1.37801222800458 | 0.000518702655498103 | XP_004924776.1 | elongation of very long chain fatty acids protein AAEL008004 |
| LOC134201066 | 275.825464593745 | 3.12175857518138 | 0.000558861341214597 | | uncharacterized LOC134201066 |
| CECB1 | 26.2064024848729 | 2.96205869469054 | 0.000568311197976321 | NP_001037460.1,<br>XP_062532230.1,<br>XP_062532229.1 | cecropin-B precursor<br>cecropin-B isoform X2<br>cecropin-B isoform X1 |
| LOC732910 | 9434.07918058502 | -1.53369093025758 | 0.000591736570114548 | NP_001040376.1,<br>XP_062528333.1,<br>XP_062528332.1,<br>XP_062528331.1 | phosphoribosylaminoimidazole carboxylase, phosphoribosylaminoimidazole succinocarboxamide synthetase,<br>phosphoribosylaminoimidazole carboxylase, phosphoribosylaminoimidazole succinocarboxamide synthetase isoform X1,<br>phosphoribosylaminoimidazole carboxylase, phosphoribosylaminoimidazole succinocarboxamide synthetase isoform X1<br>phosphoribosylaminoimidazole carboxylase, phosphoribosylaminoimidazole succinocarboxamide synthetase isoform X1 |
| SDH2b | 332.664891141925 | -1.29262662138697 | 0.000591736570114548 | NP_001188510.1 | sorbitol dehydrogenase-2b |
| LOC101747176 | 1828.8213394769 | -1.30783294940381 | 0.000621223169726979 | XP_062531196.1 | homogentisate 1,2-dioxygenase |
| LOC101745620 | 138.565510112396 | 1.35742878931259 | 0.000621223169726979 | XP_004928576.1 | zinc finger protein GLI1 |
| jhe1 | 2197.55184994261 | 3.02095560637419 | 0.000621223169726979 | NP_001037027.1 | juvenile hormone esterase 1 |
| LOC101746745 | 97.2765430598348 | 3.97287003990289 | 0.00064843931593787 | NP_001296488.1,<br>XP_004922544.1 | low molecular 30 kDa lipoprotein PBMHP-12-like<br>low molecular 30 kDa lipoprotein PBMHP-12-like isoform X1 |
| BmILP | 208.444134147143 | 2.24833305023513 | 0.000650671327182327 | NP_001233285.1,<br>XP_012548888.1 | insulin-like peptide<br>insulin-like peptide isoforom X1 |
| LOC101746583 | 1056.37670843101 | -1.28830302197189 | 0.000682722319980968 | XP_021206232.1,<br>XP_004930190.1 | sphingomyelin phosphodiesterase isoform X1<br>sphingomyelin phosphodiesterase isoform X2 |

| | | | | | |
|---|---|---|---|---|---|
| LOC101746637 | 452.486271556465 | -1.32165412196399 | 0.000696358114244455 | XP_062524290.1, XP_004930937.1 | uncharacterized protein LOC101746637 |
| LOC101740965 | 1681.54266970761 | -1.24185604971243 | 0.000699467980362457 | XP_004926194.2 | myrosinase 1 |
| LOC101747054 | 5667.59433024327 | 2.36118389735761 | 0.00070400381729456 | XP_037872304.1 XP_062528934.1 | cytosolic 10-formyltetrahydrofolate dehydrogenase |
| CecB1 | 59.0048214246738 | 3.0349485485554 | 0.000763790277200023 | NP_001096031.1 | cecropin-B |
| LOC692912 | 3568.34334249227 | -1.33950508903666 | 0.00079284552148625 | NP_001040220.1 | triacylglycerol lipase |
| LOC119630291 | 63.0045136671452 | -1.73127864662378 | 0.000796459721727147 | | U5 spliceosomal RNA |
| LOC101742944 | 247.771750147133 | 1.05146636553602 | 0.000841133113607589 | XP_004929408.2 | uncharacterized protein LOC101742944 |
| LOC119630540 | 31.5369406759354 | 2.40704010785873 | 0.000895140243969384 | XP_037876153.1 | uncharacterized protein LOC119630540 |
| LOC101739681 | 96.7771467023697 | 2.45044014346217 | 0.000897769203119373 | XP_004926104.1 | cecropin-B |
| LOC101740569 | 573.297601242334 | 2.20187704668074 | 0.000970846463727197 | XP_004934355.1 | luciferin 4-monooxygenase |
| LOC101745731 | 3404.2958437151 | 1.34151315741346 | 0.000978843997041915 | XP_004930183.1, XP_004930184.1, XP_004930185.1 | translocating chain-associated membrane protein 1 |
| LOC101739958 | 51.1964408404379 | 2.88177820877923 | 0.000984279578622466 | XP_004926106.1 | cecropin-B-like |
| LOC692643 | 39.3745501780009 | 1.9365004386584 | 0.00100099447382406 | NP_001037088.1, XP_062531511.1 | Hemolin hemolin isoform X1 |
| LOC101740726 | 303.343478048845 | 4.22000492631472 | 0.00101625257887791 | XP_004926112.1 | uncharacterized protein LOC101740726 |
| LOC101744519 | 234.241941806551 | -1.78303586542485 | 0.00107186674170481 | XP_021205750.2, XP_004929418.2 | neutral ceramidase isoform X1 neutral ceramidase isoform X2 |
| LOC101744862 | 277.052474947882 | 1.16229576338391 | 0.00110121237802109 | XP_004930603.1 | transmembrane emp24 domain-containing protein 3 |
| LOC101735904 | 6519.24958021326 | 2.51341281591905 | 0.00110121237802109 | NP_001274758.1 | low molecular mass 30 kDa lipoprotein 19G1-like |
| LOC101739439 | 126.475972337197 | 1.12862368852367 | 0.00110121237802109 | XP_062532036.1, XP_004923418.1 | piggyBac transposable element-derived protein 4 isoform X1, piggyBac transposable element-derived protein 4 isoform X2 |
| LOC101743964 | 480.148120747042 | 1.02066950690879 | 0.00113919056890342 | XP_037875986.1 | protein FAM98A |
| LOC732871 | 1187.62999271159 | 1.09060685416085 | 0.00119055298304697 | NP_001040339.1, XP_062526328.1, XP_062526329.1, XP_062526331.1 | GTP binding protein |
| LOC101738524 | 214.104038239588 | -1.38373251555354 | 0.00119055298304697 | XP_004924912.1 | uncharacterized protein LOC101738524 |
| LOC110386433 | 91.1605023616273 | 2.70749032943547 | 0.00136365946619486 | XP_021208385.1 | chymotrypsin inhibitor SCI-III-like |
| LOC692974 | 214.994291649882 | 1.14536125048321 | 0.00139922455651033 | NP_001040278.1 | stromal cell-derived factor 2 precursor |

| | | | | | |
|---|---|---|---|---|---|
| LOC101744406 | 79.8674818355076 | -1.20696854110779 | 0.00139922455651033 | XP_062531407.1, XP_012545692.2 | putative fatty acyl-CoA reductase CG5065 |
| LOC101737145 | 46.5258084004188 | -1.19080909189869 | 0.00151023439001079 | XP_004923866.1 | myrosinase 1 |
| LOC101740782 | 164.404391712532 | -1.03075297203375 | 0.0015152685864598 | XP_004927346.1, XP_062531770.1 | Kv channel-interacting protein 1 |
| LOC105842288 | 43.3445282407669 | 4.28727743580757 | 0.00157470883755692 | XP_012549781.2 | uncharacterized protein LOC105842288 |
| LOC101745431 | 129.43307860596 | -1.03334080193478 | 0.00159171459453156 | XP_037872407.1, XP_037872408.1 | iron-sulfur cluster co-chaperone protein HscB isoform X1 iron-sulfur cluster co-chaperone protein HscB isoform X2 |
| LOC101743217 | 1585.24369288826 | -1.76079020465766 | 0.00177800932362558 | XP_004924938.1 | glycine cleavage system H protein |
| LOC101737070 | 1257.57475911845 | 1.00690245851131 | 0.00181358130738267 | XP_004928888.1 | transmembrane emp24 domain-containing protein 2 |
| LOC110385036 | 1403.48412193855 | -1.17762359542924 | 0.00183802076320103 | XP_062532208.1 | Uncharacterized protein LOC110385036 |
| LOC101741449 | 659.946065348288 | 1.44359798764013 | 0.00186597171927653 | XP_062531344.1, XP_021206640.1, XP_004930776.1, XP_062531346.1 | putative fatty acyl-CoA reductase CG5065 |
| LOC732945 | 5877.10234835179 | -1.10280542811089 | 0.00191628663930323 | NP_001040409.1 | eukaryotic translation initiation factor 4E-binding protein 2 |
| Lzm | 8318.48710235367 | 2.21693175304055 | 0.00192049599274162 | NP_001037448.1 | lysozyme |
| Ppib | 1930.03814166501 | 1.32283446638995 | 0.00217641168881657 | NP_001040479.1 | peptidylprolyl isomerase B |
| LOC101739002 | 1300.57617039862 | -1.02756829542949 | 0.00242176227281417 | XP_012552187.1, XP_037873969.1, XP_062530368.1 | PHAF1 protein CG7083 |
| LOC101741082 | 13201.5164940306 | -1.45553604671936 | 0.00255758796547595 | XP_004931117.1, XP_062526098.1, XP_021206910.1, XP_062526099.1 | bifunctional purine biosynthesis protein ATIC |
| LOC101747018 | 379.81073279887 | 1.90263496810121 | 0.00256078942166503 | NP_001266366.1 | low molecular mass 30 kDa lipoprotein 19G1-like |
| Atg8 | 2051.42167248173 | -1.02665228811709 | 0.00265696367537304 | NP_001040244.1 | autophagy related protein Atg8 |
| LOC101745576 | 83.1730647616992 | -1.17593921998324 | 0.00267865543251817 | XP_004929231.1 | prostatic acid phosphatase |
| LOC101736379 | 51.1789686341305 | 1.73526042218496 | 0.00267865543251817 | XP_062532283.1, XP_012551634.2 | myrosinase 1 |
| LOC101739328 | 564.656954011342 | -3.92047826417977 | 0.00269638443275621 | XP_004930761.1 | putative fatty acyl-CoA reductase CG5065 |
| LOC101739287 | 196.543676532568 | -1.01610526154237 | 0.0027162388625688 | XP_004933641.2 | divergent protein kinase domain 2A |
| LOC119629051 | 42.1811882526311 | -1.61477558245157 | 0.00277977158115765 | | uncharacterized LOC119629051 |

| | | | | | |
|---|---|---|---|---|---|
| tan | 334.241227798048 | -1.09538794497906 | 0.00280398108104768 | NP_001170882.1, XP_021207941.1, XP_012551408.1 | C45 family peptidase tan C45 family peptidase tan isoform X2 C45 family peptidase tan isoform X1 |
| UGT340C1 | 35.0461297598063 | 2.50340761432223 | 0.00281696041097427 | NP_001243962.1 | UDP-glycosyltransferase UGT340C1 |
| LOC101736374 | 61.8339851066285 | -3.96996324962868 | 0.00282972402357309 | XP_062527164.1, XP_004931634.1 | dipeptidase 1 |
| Leb3 | 1330.82902575126 | 1.84214592981215 | 0.00285796764229343 | NP_001119732.2 | lebocin |
| LOC101741624 | 63.43995659193 | -1.32576191473173 | 0.00286212489010447 | XP_004925623.2, XP_037874397.1 | neuroendocrine convertase 1 isoform X1 neuroendocrine convertase 1 isoform X2 |
| LOC101743515 | 55.9144151398918 | -1.14853727929378 | 0.00302191191193839 | XP_012549756.1 | transcription factor SUM-1 |
| LOC101737915 | 350.929731816692 | -1.29956616342198 | 0.00304759425198301 | NP_001296534.1, XP_037873016.1, XP_062529503.1, XP_037873018.1, XP_037873017.1 | dopamine N-acetyltransferase-like dopamine N-acetyltransferase-like isoform X2 dopamine N-acetyltransferase-like isoform X1 dopamine N-acetyltransferase-like isoform X2 dopamine N-acetyltransferase-like isoform X2 |
| LOC101741659 | 312.408806576837 | -1.34807339427852 | 0.00312634141201518 | XP_004924568.2, XP_062532060.1 | L-threonine ammonia-lyase |
| Hsp90 | 2954.01722591377 | 2.24209747335606 | 0.00314423406831213 | NP_001036876.1 | Heat shock protein 83 |
| LOC101745172 | 861.207390139352 | -1.4538603379023 | 0.00320829479099481 | XP_062530008.1, XP_004922382.1 | holotricin-3 |
| LOC134198658 | 420.59290867108 | -1.41903246438695 | 0.00329477157638712 | XP_012549965.1, XP_012549964.1, XP_021206769.2 | neural-cadherin-like isoform X2 neural-cadherin-like isoform X1 neural-cadherin-like isoform X3 |
| LOC105841804 | 112.234806752859 | 1.48218224419347 | 0.00349808169754909 | XP_012546790.1, XP_012546788.1 | serine protease inhibitor swm-1-like isoform X2 serine protease inhibitor swm-1-like isoform X1 |
| Lp-c12 | 1991.87421670531 | 2.37953323748553 | 0.00357734046784558 | NP_001095196.1, XP_012550486.1 | low molecular lipoprotein 30K pBmHPC-12 |
| LOC101737000 | 1620.72634033352 | -1.51003378716437 | 0.00358369375676706 | XP_037874820.1 | pancreatic lipase-related protein 2 |
| LOC101745375 | 1237.37727391023 | 1.17065496661549 | 0.00383314912965068 | XP_004925862.1 | ommochrome-binding protein |
| glv3 | 1075.95060499195 | 3.29578020506277 | 0.0038341661465414 | NP_001093312.1, XP_062532688.1 | gloverin 3 precursor gloverin 3 isoform X1 |
| LOC101742570 | 85.2548322212345 | -1.18907518398172 | 0.00383613583188519 | XP_004933525.1 | Growth arrest and DNA damage-inducible 45 |

| | | | | | |
|---|---|---|---|---|---|
| LOC101741765 | 16.9559637542661 | 1.07227373604172 | 0.00404591790485433 | XP_012545598.2,<br>XP_012545599.2 | netrin receptor UNC5C isoform X1<br>netrin receptor UNC5C isoform X2 |
| LOC101737095 | 1655.75187169649 | 1.01168837805783 | 0.00406515737842575 | XP_004923047.1 | dolichyl-diphosphooligosaccharide--protein glycosyltransferase subunit STT3A |
| LOC101743583 | 12224.8492219742 | -1.04063767298407 | 0.004077590719895 | XP_004924304.1,<br>XP_021202424.1 | MAP kinase-interacting serine/threonine-protein kinase 1 isoform X1<br>MAP kinase-interacting serine/threonine-protein kinase 1 isoform X2 |
| Ncd | 43.9724230248664 | 1.60546787659103 | 0.00423987299875065 | NP_001119723.1 | kinesin-like protein Ncd |
| Phf5a | 40.37086416548 | 1.15749509401854 | 0.00447693601869955 | NP_001091825.1 | PHD finger protein 5A |
| LOC110386865 | 88.0582924806431 | -1.36618964955445 | 0.00447693601869955 | | uncharacterized protein LOC110386865 |
| LOC101744689 | 24.7246352258155 | -1.19582925067507 | 0.00487252440668507 | XP_004923014.1 | protein takeout |
| LOC105842565 | 735.188087995294 | -1.25507204846131 | 0.00509436521831679 | XP_012551806.1 | uncharacterized protein LOC105842565 |
| LOC101740246 | 186.781535647957 | 1.06285136788034 | 0.00524192517169641 | XP_012549706.1 | prefoldin subunit 5 |
| P450 | 20.1838786462904 | -1.5353934076656 | 0.0054108857815586 | NP_001140197.1,<br>XP_037869524.1,<br>XP_062526789.1 | farnesoate epoxidase precursor<br>farnesoate epoxidase isoform X1<br>farnesoate epoxidase isoform X1 |
| H4-l | 1352.62571107792 | 1.20537728524078 | 0.00590632524772817 | NP_001153669.1,<br>XP_062531484.1 | histone H4-like protein<br>histone H4-like protein isoform X1 |
| LOC101741887 | 138.441552747413 | 1.08543355328475 | 0.00593368042581214 | XP_004932924.1 | exonuclease 1 |
| LOC119630287 | 98.8456450215575 | -1.50740610557543 | 0.00620681034377556 | | U5 spliceosomal RNA |
| LOC101739666 | 76.5941734206737 | -1.06045635857991 | 0.00644562851961729 | XP_021203491.2,<br>XP_012546467.2,<br>XP_062529283.1 | protein eva-1 isoform X2<br>protein eva-1 isoform X1<br>protein eva-1 isoform X3 |
| LOC101738415 | 125.563165939178 | 1.0352195291675 | 0.00672514960350716 | XP_004932689.1 | dehydrodolichyl diphosphate synthase complex subunit Nus1 |
| LOC692570 | 1468.40370105883 | -1.11421341288065 | 0.00689373396971955 | XP_037870226.1,<br>XP_037870227.1,<br>XP_037870228.1 | ATP-binding cassette sub-family G member 1 isoform X1,<br>ATP-binding cassette sub-family G member 1 isoform X1,<br>ATP-binding cassette sub-family G member 1 isoform X2 |
| Fkbp45 | 1941.32057869908 | 1.05802950376152 | 0.00694465627241942 | NP_001037356.1 | 45 kDa immunophilin FKBP45 |
| LOC101740111 | 175.802071116582 | -1.38284888364043 | 0.00734507071038829 | XP_004930976.1 | zinc transporter ZIP10 |
| LOC119630290 | 100.165069772351 | -1.45778214682253 | 0.00792451517439642 | | U5 spliceosomal RNA |

| | | | | | |
|---|---|---|---|---|---|
| LOC101746885 | 14.2434880175348 | 4.08176006463196 | 0.00798676752418091 | NP_001296545.1, XP_062530138.1 | low molecular mass 30 kDa lipoprotein 19G1-like precursor<br>low molecular mass 30 kDa lipoprotein 19G1-like isoform X1 |
| Attacin1 | 134.480999048215 | 1.7214574677815 | 0.00850383898819812 | NP_001037006.1, XP_062524867.1 | Attacin precursor<br>Attacin isoform X1 |
| Ugt2 | 6569.27251367387 | 1.27210207295051 | 0.00877213707861194 | NP_001127730.1 | uridine diphosphate glucosyltransferase 2 |
| LOC134200902 | 27.245386128843 | 4.13223477737581 | 0.00884383675738066 | | Uncharacterized protein LOC134200902 |
| CPR39 | 21.0503161442335 | 1.75327612393147 | 0.00889090066051583 | NP_001166714.1 | cuticular protein RR-1 motif 39 |
| LOC101743449 | 15.8875903494252 | 3.55120200205614 | 0.00902840180493364 | XP_062527947.1 | chorion class A protein L12-like |
| UGT10287A | 87.6026792233279 | 1.35087565078637 | 0.0100370432938898 | NP_001182387.1, XP_012550240.1, XP_037867794.1 | UDP-glucosyltransferase precursor<br>UDP-glucosyltransferase isoform X1<br>UDP-glucosyltransferase isoform X1 |
| LOC692945 | 2146.20925818068 | 1.01018970645088 | 0.010043738663799 | NP_001040250.1 | kazal-type proteinase inhibitor |
| LOC101735336 | 895.403146180093 | -1.28220776773275 | 0.0104890461981821 | XP_037872163.1, XP_037872164.1 | calcium/calmodulin-dependent protein kinase kinase 2 isoform X1<br>calcium/calmodulin-dependent protein kinase kinase 2 isoform X2 |
| LOC101740603 | 827.822148382949 | -1.11944996333453 | 0.0105302611211455 | XP_004930147.2, XP_012549296.2, XP_062524701.1 | uncharacterized protein LOC101740603 |
| LOC110385258 | 102.887085478833 | -1.12366304796335 | 0.0105302611211455 | | uncharacterized protein LOC110385258 |
| LOC101738029 | 1915.59430757884 | 1.99076010110067 | 0.010817704496116 | XP_037871281.1, XP_062528078.1, XP_062528075.1, XP_062528080.1, XP_062528073.1, XP_062528086.1, XP_062528089.1, XP_062528088.1, XP_062528084.1, XP_062528072.1, XP_062528085.1, XP_037871286.1, XP_062528090.1, | AT-rich interactive domain-containing protein 2 |

| | | | | | |
|---|---|---|---|---|---|
| | | | | XP_062528079.1, XP_062528091.1, XP_062528074.1, XP_062528076.1, XP_062528092.1, XP_062528077.1, XP_062528082.1, XP_062528094.1, XP_062528083.1, XP_062528081.1, XP_062528093.1, XP_062528087.1 | |
| LOC101735781 | 20.6298414039706 | 3.00348569290385 | 0.01084529929098 | XP_004924962.1 | chorion class A protein L12-like |
| LOC101745043 | 372.673776091395 | -1.10122197698319 | 0.01084529929098 | XP_062528455.1, XP_037871719.1, XP_062528454.1, XP_037871718.1 | kynurenine formamidase isoform X2 kynurenine formamidase isoform X2 kynurenine formamidase isoform X2 kynurenine formamidase isoform X1 |
| LOC101736801 | 255.199950658916 | -1.23304067617188 | 0.0111101606930288 | XP_004926504.3 | homeobox protein Hox-B5a |
| LOC101746802 | 694.446817331485 | -1.17028329389695 | 0.0115620224758196 | XP_004926772.1 | aldo-keto reductase AKR2E4-like |
| LOC101746863 | 29.2422467764882 | 3.20966219353083 | 0.0121379555212965 | XP_004930728.1 | major facilitator superfamily domain-containing protein 6 |
| LOC101737039 | 1748.79448232089 | -2.72822587282327 | 0.012332903275809 | XP_021208351.1, XP_062529619.1, XP_062529636.1, XP_062529633.1, XP_062529623.1, XP_062529664.1, XP_062529657.1, XP_062529632.1, XP_062529668.1, XP_062529629.1, XP_062529642.1, XP_062529638.1, | adenosylhomocysteinase-like 1 |

| | | | | | |
|---|---|---|---|---|---|
| | | | | XP_062529655.1, XP_062529649.1 | |
| LOC101740613 | 1847.30903478425 | 1.47616765143695 | 0.012365155968382 | XP_004932707.1 | uncharacterized protein LOC101740613 |
| LOC119628922 | 1079.86854619839 | 4.5983153437266 | 0.0123949678093405 | XP_037868962.2 | uncharacterized protein LOC119628922 |
| LOC101745500 | 85.7404721324844 | 1.84918080927093 | 0.0124661632319172 | XP_004933911.2 | heat shock protein 68 |
| LOC105842846 | 194.048573969737 | 1.15837440547303 | 0.013446887797398 | XP_062526083.1, XP_037868874.1, XP_037868872.1, XP_062526084.1, XP_037868873.1 | bombyxin B-3-like X2<br>bombyxin A-1 homolog isoform X4<br>bombyxin G-1-like isoform X1<br>bombyxin A-2 homolog isoform X5<br>bombyxin A-3 homolog isoform X3 |
| LOC101739550 | 19.6074093114765 | 2.26680411924369 | 0.0139892455995677 | XP_004929547.5 | ecdysone oxidase |
| LOC101736302 | 3129.54958580225 | -1.04475547140136 | 0.0141692603510225 | XP_004922006.1 | uncharacterized LOC101736302 |
| LOC101740411 | 1701.13213216103 | -1.04856518618237 | 0.0142389208143404 | XP_004927149.1 | uncharacterized LOC101740411 |
| LOC101742864 | 138.384020992652 | -1.16154709211944 | 0.014562340764496 | XP_062527373.1, XP_062527374.1, XP_037870441.1, XP_037870442.1 | uncharacterized LOC101742864 |
| LOC101745562 | 477.900622377654 | -1.06988630652565 | 0.0152307133174317 | XP_004924671.1 | 2-Hydroxyacid oxidase 1 |
| LOC101744151 | 258.781667263928 | 1.05418314933205 | 0.015572004615756 | XP_037866899.1 | solute carrier family 35 member B1 homolog |
| LOC778474 | 1516.32074285667 | -1.09756936857433 | 0.0155951395789379 | NP_001091769.1, XP_062528337.1, XP_012552770.1 | putative amine transporter<br>putative amine transporter isoform X2<br>putative amine transporter isoform X1 |
| CecA | 44.7104050918666 | 1.91873527294572 | 0.0156267710590848 | NP_001037462.1 | cecropin A |
| LOC101742735 | 808.837300709598 | 1.16459770836018 | 0.0156993587361761 | XP_037875551.1 | chromatin assembly factor 1 p55 subunit |
| LOC778461 | 2345.83586244131 | 1.19799474821722 | 0.0166183495069841 | NP_001091760.1 | signal sequence receptor subunit 1 l(1)G0320 |
| LOC101735343 | 119.554251338412 | -1.54653913934978 | 0.0171618266733697 | XP_004930731.1, XP_062531290.1 | ankyrin-3 |
| LOC119630671 | 56.8791766464918 | 2.90071209963696 | 0.017223706844201 | XP_037876693.2 | 52 kDa repressor of the inhibitor of the protein kinase |
| LOC101737251 | 2123.67621208589 | 1.61479643695485 | 0.0176729962088477 | XP_037876088.2, XP_062531740.1, XP_062531741.1, XP_062531742.1 | PH-interacting protein isoform X1<br>PH-interacting protein isoform X2<br>bromodomain and WD repeat-containing protein 1 isoform X3<br>PH-interacting protein isoform X4 |

| | | | | | |
|---|---|---|---|---|---|
| LOC101743494 | 24253.7926780067 | -1.1307887138298 | 0.0176729962088477 | XP_004924493.1, XP_012544431.1 | C-1-tetrahydrofolate synthase, cytoplasmic isoform X1 C-1-tetrahydrofolate synthase, cytoplasmic isoform X2 |
| Ci-b1 | 9236.14526874108 | 1.24719938435267 | 0.0182324466963469 | NP_001119717.2 | chymotrypsin inhibitor SCI-II |
| Hsp70 | 115.941462623527 | 1.65168042410604 | 0.018395015526781 | XP_037876891.1 | Hsp70 |
| LOC101744260 | 138.709147854951 | -1.80259782722455 | 0.018441294125608 | XP_004925993.2 | putative fatty acyl-CoA reductase CG5065 |
| LOC119629757 | 116.169385262675 | -2.9668463967865 | 0.0184464349840946 | | uncharacterized LOC119629757 |
| LOC101743224 | 76.8068507589612 | 1.803613168393 | 0.0186901959086726 | XP_004926758.1 | attacin-like |
| LOC692831 | 724.351057283324 | -1.09444170226632 | 0.0187304522569844 | NP_001040141.1, NP_001166839.1, NP_001166840.1, XP_012553085.2, XP_012553086.2, XP_037876126.1 | acyl-CoA delta-11 desaturase isoform 1 acyl-CoA delta-11 desaturase isoform 2 acyl-CoA delta-11 desaturase isoform 3 acyl-CoA delta-11 desaturase isoform X1 acyl-CoA delta-11 desaturase isoform X1 acyl-CoA delta-11 desaturase isoform X2 |
| LOC119630260 | 11.986977660344 | 3.14360834266268 | 0.0190666918905837 | | uncharacterized LOC119630260 |
| LOC101742047 | 322.331726767703 | 1.27921087661129 | 0.0199512180619513 | XP_037872193.1 | fatty acid synthase |
| LOC101738723 | 42.951274530755 | 1.72058086540916 | 0.0200455144531233 | XP_062531525.1 | synaptotagmin-12 |
| LOC101744993 | 7519.20013607904 | -1.09897191027085 | 0.0204746093207336 | XP_062528392.1, XP_004928658.1, XP_062528393.1, XP_062528391.1 | amidophosphoribosyltransferase |
| LOC692784 | 1521.4514052088 | -1.40986727060702 | 0.0211679296724431 | NP_001037382.1 | uricase |
| LOC101741875 | 2125.43876130958 | 2.23964218499818 | 0.0212509547873956 | XP_004928635.1 | ras-related protein Rab-9B |
| LOC101746901 | 80.0570772438471 | -1.47747666743923 | 0.0212509547873956 | XP_004926641.1 | uncharacterized protein LOC101746901 |
| Tyms | 74.6144251920115 | 1.82233354166967 | 0.0212650507766771 | NP_001040281.1, NP_001166836.1 | thymidylate synthase |
| PGRP-S5 | 508.118577984121 | -1.31597343409624 | 0.0213603437951237 | NP_001036858.1, XP_062528827.1 | peptidoglycan recognition protein S6 precursor peptidoglycan recognition protein S6 isoform X1 |
| LOC134201672 | 14.9208147945466 | 3.23975129158921 | 0.0221861278834124 | XP_062532898.1 | Uncharacterized protein LOC134201672 |
| LOC101738787 | 372.433525201031 | -1.14943918521123 | 0.0222168823435213 | XP_004923610.1 | nuclear factor interleukin-3-regulated protein |
| LOC134201454 | 1357.89798903564 | -1.12567278514481 | 0.0225945828535303 | XP_062532210.1 | involucrin-like |
| UGT10287B | 48.3702086791745 | 3.40616212753603 | 0.0226448415338428 | NP_001182388.1 | UDP-glucosyltransferase |
| LOC101746808 | 117.615463610939 | -1.31155975767193 | 0.0229670461804352 | XP_004929726.1 | uncharacterized LOC101746808 |
| RnrS | 400.588517453661 | 1.73061990360022 | 0.0232575280030518 | NP_001244266.1 | ribonucleoside-diphosphate reductase subunit M2 |

| | | | | | |
|---|---|---|---|---|---|
| LOC101735832 | 203.551513322014 | 1.74400332414866 | 0.0234309959868219 | XP_037876874.1 | heat shock protein 68 |
| LOC101736553 | 11.3530263475136 | -2.09448318084491 | 0.0236397062421985 | XP_012552225.1,<br>XP_021208619.1 | pancreatic lipase-related protein 2 isoform X2<br>pancreatic lipase-related protein 2 isoform X1 |
| LOC101740858 | 157.319035470788 | 1.48089514999165 | 0.0243526149204144 | XP_004923890.1 | chromatin accessibility complex protein 1 |
| LOC134199436 | 17.4378570969179 | 1.84953495221627 | 0.0243526149204144 | XP_062526266.1 | uncharacterized LOC134199436 |
| UGT50A1 | 73.5709525165124 | -1.15899441604614 | 0.0245691183010296 | NP_001243994.1,<br>XP_037873014.1,<br>XP_062529502.1 | UDP-glycosyltransferase family 50 member B3 precursor<br>UDP-glycosyltransferase UGT50A1 isoform X1<br>UDP-glycosyltransferase UGT50A1 isoform X1 |
| LOC778480 | 156.073344316312 | 1.01764965462265 | 0.0245691183010296 | NP_001091775.1 | signal recognition particle 14 |
| LOC100862772 | 22.0820827087456 | 2.48056076775057 | 0.0247348999674308 | NP_001243928.1 | heat shock protein 68 |
| LOC110385107 | 18.6584772562621 | -1.0631278826409 | 0.0247730282418018 | XP_062532094.1 | retrovirus-related Pol polyprotein from transposon 297 |
| LOC101745473 | 260.235399662765 | 1.36223185271828 | 0.0248708454566476 | XP_012546338.1 | uncharacterized protein LOC101745473 |
| LOC101737212 | 241.964476014298 | -1.04510769236494 | 0.0252303299603738 | XP_037872374.1 | alpha-tocopherol transfer protein-like |
| LOC101740267 | 1805.73210151667 | 2.20303404366232 | 0.0260106863437318 | XP_004923823.1 | septin-2 |
| LOC101745977 | 13.7294305961267 | -1.5182646435775 | 0.0261644918374421 | XP_062527187.1 | dynein intermediate chain 3, ciliary |
| LOC101738400 | 60.448128747762 | 1.36371920965892 | 0.0269517124546503 | XP_037872418.1<br>XP_037872417.1 | histone deacetylase 8 |
| LOC101738170 | 28.3809024435543 | -1.27315827038731 | 0.0274933046406919 | XP_037872578.1,<br>XP_062529448.1 | L-dopachrome tautomerase yellow-f2 isoform X1<br>L-dopachrome tautomerase yellow-f2 isoform X2 |
| LOC119630793 | 32.5898736919212 | -1.17207694905168 | 0.0276706682312038 | | uncharacterized LOC119630793 |
| Lp-c23 | 45064.9214980526 | 1.51622251556511 | 0.0283966815385098 | NP_001095198.2 | low molecular lipoprotein 30K pBmHPC-23 |
| LOC692353 | 3654.95595765909 | 1.11934124499364 | 0.0293055793389563 | NP_001036781.1 | histone H2A-like protein |
| Ahsa1 | 210.925456477966 | 1.0208557627042 | 0.029930821497103 | NP_001036909.1 | activator of 90 kDa heat shock protein ATPase homolog 1 |
| LOC119629437 | 11.7839329015224 | -1.9944586749131 | 0.0305189834513544 | | 5S ribosomal RNA |
| LOC119629571 | 25.5384505676577 | 2.43332730562361 | 0.0305189834513544 | XP_037871664.1 | uncharacterized LOC119629571 |
| glv4-like | 72.4548135131686 | 2.63932197109904 | 0.0305189834513544 | NP_001036932.1,<br>XP_062532690.1,<br>XP_062532689.1 | gloverin 4-like precursor<br>gloverin 4-like isoform X1<br>gloverin 4-like isoform X1 |
| PGRP | 29.2209647960478 | 2.30738408033578 | 0.0306027809130162 | NP_001243949.1 | peptidoglycan recognition protein |
| LOC101743336 | 55.5409681126412 | 1.49642800411611 | 0.0311953707403287 | NP_001037030.2 | cecropin-A-like |
| LOC101739241 | 697.181277834543 | 1.92575318402899 | 0.0324261149687485 | XP_012552265.3 | rolyl 3-hydroxylase sudestada1 |
| LOC119629373 | 119.33742995955 | -3.62525541996763 | 0.0325993787671155 | XP_037870978.1 | uncharacterized LOC119629373 |
| LOC733093 | 374.176639941003 | 1.5568326139202 | 0.0329688770831267 | NP_001296526.1 | heat shock protein 70 A1 |

| | | | | | |
|---|---|---|---|---|---|
| LOC101735764 | 92.675589990549 | -1.63943355314533 | 0.0330109264999492 | XP_062524454.1, XP_037867220.1, XP_037867218.1, XP_037867221.1, XP_037867219.1 | calcium and integrin-binding family member 3 isoform X3 calcium and integrin-binding family member 3 isoform X3 calcium and integrin-binding family member 3 isoform X1 calcium and integrin-binding family member 3 isoform X3 calcium and integrin-binding family member 3 isoform X2 |
| LOC101740880 | 69.1985888812151 | -1.00523676409355 | 0.0331480480548105 | XP_012549173.1, XP_021206196.1, XP_012549174.2, XP_062524662.1 | potassium voltage-gated channel subfamily KQT member 1 isoform X3 potassium voltage-gated channel subfamily KQT member 1 isoform X1 potassium voltage-gated channel subfamily KQT member 1 isoform X2 potassium voltage-gated channel subfamily KQT member 1 isoform X4 |
| LOC101736421 | 277027.034613437 | 2.82743780942066 | 0.0347542550254683 | XP_037874502.1, XP_037874486.1, XP_037874497.1, XP_037874494.1, XP_062531996.1, XP_037874506.1, XP_062531994.1, XP_037874498.1 | protein split ends isoform X5 protein split ends isoform X1 protein split ends isoform X3 protein split ends isoform X2 protein split ends isoform X8 protein split ends isoform X7 protein split ends isoform X6 protein split ends isoform X4 |
| LOC119630575 | 6448.9207824859 | -1.05028546196146 | 0.0355063350461083 | XP_062532209.1 | trichohyalin |
| LOC101742749 | 887.27135418598 | -1.0891099007833 | 0.0357615730016993 | XP_062524253.1, XP_062524251.1, XP_021206856.1, XP_062524252.1 | tyrosine aminotransferase |
| LOC101739276 | 14.1439425717369 | -2.56053806906039 | 0.0357615730016993 | XP_037870248.1, XP_004929545.3, XP_037870249.1 | ecdysone oxidase-like |
| LOC100302629 | 3533.057020847 | -2.71045058970371 | 0.0358836325992253 | XP_062531485.1, XP_062531486.1 | ecdysone oxidase |

| | | | | | |
|---|---|---|---|---|---|
| LOC101741759 | 41.586698815009 | 1.47274383889692 | 0.0359356960853331 | XP_062526252.1, XP_062526252.1, XP_021202223.2 | bombyxin A-3 homolog isoform X3 bombyxin A-3 homolog isoform X2 bombyxin A-3 homolog isoform X1 |
| LOC101740124 | 37.4681394951867 | 1.60362009457746 | 0.0362301613867487 | XP_037872052.1 | uncharacterized LOC101740124 |
| LOC101746558 | 13.0300817945233 | 2.76349481036059 | 0.0371223219928386 | XP_021202430.2 | cell adhesion molecule Dscam2 |
| LOC101747088 | 33.7263862125189 | -1.55401383002557 | 0.0393190805169851 | XP_004931356.1, XP_004931357.1 | ataxin-2 homolog isoform X1 ataxin-2 homolog isoform X2 |
| LOC119629207 | 653.140368642991 | -1.25281004175177 | 0.0393190805169851 | | small nucleolar RNA SNORA57 |
| LOC134198949 | 13.2084428486214 | -1.42415165426306 | 0.0393260446387141 | | uncharacterized LOC134198949 |
| LOC134200408 | 11.0781655796893 | -1.3311610597263 | 0.0398033325752259 | | uncharacterized LOC134200408 |
| LOC101747037 | 2427.73534412509 | -1.11270192710995 | 0.0415351418954165 | XP_012547004.1 | cysteine dioxygenase type 1 |
| PAH | 4921.47388352778 | -1.27985090720794 | 0.0415649745038296 | NP_001274766.1, XP_004924935.1 | phenylalanine hydroxylase phenylalanine hydroxylase isoform X1 |
| Crt | 10895.2889081311 | 1.14250003501469 | 0.0415649745038296 | NP_001037075.1 | calreticulin |
| LOC101735884 | 1343.21666492908 | -1.22296955661905 | 0.042043978879422 | XP_012548006.1 | nose resistant to fluoxetine protein 6 |
| Ibm1 | 18.3494635254597 | -1.14100555834214 | 0.042443677260338 | NP_001159813.1 | IAP-binding motif 1 |
| LOC134200009 | 26.7165873530477 | -1.54799622424247 | 0.0434921505709454 | | uncharacterized LOC134200009 |
| LOC101740852 | 24.8745647242781 | -1.09114585742916 | 0.0444748894977591 | XP_062526654.1, XP_021209147.1 | cytochrome P450 18a1 |
| LOC101737902 | 298.919371438918 | 1.18437288769685 | 0.0444799057809028 | XP_012544010.1 | arylsulfatase B |
| LOC101739986 | 784.858303637723 | 1.24811748064522 | 0.0444799057809028 | XP_037875636.1 | galectin-4 |
| LOC101744263 | 18.0595267400822 | 1.59797291282342 | 0.0447806400295644 | XP_037868996.1 | uncharacterized LOC101744263 |
| LOC101736743 | 721.733741010515 | -2.2438157469229 | 0.0447806400295644 | XP_037869085.1 | elongator complex protein 2 |
| LOC119630090 | 21.5983301201252 | -2.36183110683812 | 0.045031147312186 | XP_037874317.1 | uncharacterized LOC119630090 |
| LOC101746848 | 114.692177299774 | 1.13594589223875 | 0.0450925526693969 | XP_037873653.1, XP_062529882.1, XP_037873654.1, XP_037873655.1 | DNA replication licensing factor MCM4 isoform X1, DNA replication licensing factor MCM4 isoform X4, DNA replication licensing factor MCM4 isoform X2, DNA replication licensing factor MCM4 isoform X3 |
| LOC101743616 | 29.2198302963825 | -1.76112741473822 | 0.0473497222844879 | XP_062532139.1, XP_012545358.1, XP_012545362.1, XP_062532140.1 | nephrin isoform X1 nephrin isoform X1 nephrin isoform X2 synaptogenesis protein syg-2 isoform X3 |
| LOC101741714 | 317.922334672253 | 1.32840083592265 | 0.0480485995932583 | XP_004925552.1 | ribonucleoside-diphosphate reductase large subunit |

| | | | | | |
|---|---|---|---|---|---|
| LOC101746872 | 49.989656274428 | 2.79077116656523 | 0.0481455125173657 | XP_004932419.1 | uncharacterized protein LOC101746872 |
| LOC101737302 | 30.3188193050843 | -1.3070506758309 | 0.0483049666086591 | XP_004927325.1 | uncharacterized protein LOC101737302 |
| LOC101741979 | 132.874232571034 | 1.17434195724387 | 0.0489167358305642 | XP_004931317.1 | deoxyuridine 5'-triphosphate nucleotidohydrolase |
| LOC134200639 | 42.8116983391009 | 1.45729475509087 | 0.0491109542499995 | | uncharacterized protein LOC134200639 |
| LOC101739035 | 1282.36483423002 | -1.10761828775453 | 0.0494024316307357 | XP_021205499.1,<br>XP_037872308.1,<br>XP_037872306.1 | small G protein signaling modulator 2 isoform X3<br>small G protein signaling modulator 2 isoform X2<br>small G protein signaling modulator 2 isoform X1 |
| LOC105841601 | 392.238055182754 | 1.57664001595501 | 0.0495095442934927 | XP_062529529.1,<br>XP_062529530.1,<br>XP_037873095.1,<br>XP_037873096.1,<br>XP_062529528.1 | protein charlatan isoform X1<br>protein charlatan isoform X2<br>protein charlatan isoform X1<br>protein charlatan isoform X1<br>protein charlatan isoform X1 |
| LOC119630502 | 24.8368697860129 | -1.02802310139755 | 0.0498542557990677 | | uncharacterized protein LOC119630502 |
| LOC101740018 | 225.77666206631 | -1.08542194162207 | 0.0504519269600634 | XP_012548957.2,<br>XP_004929950.2 | forkhead box protein L2 isoform X2<br>forkhead box protein L2 isoform X1 |
| LOC101736314 | 2017.39670686637 | -1.14616280249645 | 0.0512944366463836 | XP_062530751.1,<br>XP_062530755.1,<br>XP_062530754.1,<br>XP_062530753.1,<br>XP_037874501.1,<br>XP_062530752.1 | cytochrome P450 6B1 |
| LOC101741905 | 32859.3058271771 | 2.59283395995493 | 0.0519543915389649 | XP_004924019.1,<br>XP_004924018.1 | uncharacterized protein LOC101741905 isoform X2,<br>uncharacterized protein LOC101741905 isoform X1 |
| LOC101741300 | 81.3663099204056 | 1.45318689210324 | 0.052205566522537 | XP_004926677.1 | deoxynucleoside kinase |
| GlcNAcase3 | 683.264779184467 | 1.05413288378381 | 0.0526008020400793 | NP_001037096.1,<br>XP_062525533.1 | beta-N-acetylglucosaminidase 3 precursor<br>beta-N-acetylglucosaminidase 3 isoform X1 |
| LOC101744799 | 1382.47962393261 | 2.93232565301703 | 0.0531212899585852 | XP_021204789.1,<br>XP_062527713.1 | iduronate 2-sulfatase |
| LOC101744006 | 32.3472676039629 | -1.01598106780587 | 0.0532472529977994 | XP_004933139.1 | uncharacterized protein LOC101744006 |
| LOC101744691 | 12180.8676687929 | 1.71590218100421 | 0.0532472529977994 | XP_004923175.2 | heterogeneous nuclear ribonucleoprotein U-like protein 1 |
| LOC101743940 | 36877.4838548098 | 1.91049223809589 | 0.0534963796006792 | XP_062528562.1,<br>XP_004928482.1 | EF-hand domain-containing protein 1 |
| AGPAT5 | 27.8409254128827 | -1.70536004920594 | 0.0536273362387192 | NP_001268824.1 | 1-acylglycerol-3-phosphate O-acyltransferase 5 |

| | | | | | |
|---|---|---|---|---|---|
| LOC119630248 | 13.548866802057 | 1.94966498927059 | 0.0543268971090893 | XP_062531086.1 | serine/arginine repetitive matrix protein 1-like |
| LOC134199185 | 1897.52268948325 | 2.49737271936273 | 0.0547353449665124 | XP_062525458.1,<br>XP_062525456.1,<br>XP_062525460.1,<br>XP_062525459.1,<br>XP_062525457.1 | uncharacterized protein LOC134199185 |
| Hsp20.4 | 126.334994987812 | 1.89312661785977 | 0.0550670611385422 | NP_001037038.1 | heat shock protein 20.4 |
| LOC119630311 | 15.8664935605926 | -1.49185412721001 | 0.0556031367875773 | XP_037875193.1 | putative nuclease HARBI1 |
| LOC119630288 | 182.871375282565 | -1.29887590445542 | 0.0562038296004731 | | U5 spliceosomal RNA |
| UGT340C2 | 45.9780327003637 | 1.58999762291108 | 0.0569853015850378 | NP_001243963.1 | UDP-glycosyltransferase UGT340C2 |
| LOC101740583 | 242.025450491248 | 2.31708575010245 | 0.0571573389589515 | XP_021203081.2 | chorion peroxidase |
| CPG24 | 65.678942738795 | -1.01254363906923 | 0.0572210896039784 | NP_001166782.1,<br>XP_037876993.1 | cuticular protein glycine-rich 24 precursor<br>cuticular protein glycine-rich 24 isoform X1 |
| LOC119630267 | 13.1664822655045 | 1.1761885771667 | 0.0575503324122134 | XP_037875001.1 | uncharacterized protein LOC119630267 |
| LOC119630809 | 19.3555838263061 | 1.04707506705559 | 0.057571374629263 | XP_037877261.1 | zinc finger protein 26 |
| LOC101746240 | 236.488509589913 | 1.91699185479067 | 0.0596924743521581 | XP_012544310.2 | uncharacterized protein LOC101746240 |
| LOC101745917 | 75.3923273953162 | 1.84460040699014 | 0.0606947581337444 | XP_037866530.1,<br>XP_037866531.1,<br>XP_062533116.1 | uncharacterized protein LOC101745917 isoform X1.<br>uncharacterized protein LOC101745917 isoform X2.<br>uncharacterized protein LOC101745917 isoform X3 |
| LOC134198652 | 16.3368402536688 | -1.59169078059376 | 0.0608083752362799 | XP_012549612.1 | facilitated trehalose transporter Tret1-2 homolog |
| GlcNAcase2 | 3805.20144842812 | 1.12771264612461 | 0.0609592497538929 | NP_001093291.1 | beta-N-acetylglucosaminidase 2 |
| LOC101742274 | 13.2196701635318 | 2.64530019914878 | 0.0609823232016177 | XP_004931806.2 | basic juvenile hormone-suppressible protein 2 |
| LOC101745759 | 256.318536889352 | -1.00136745615735 | 0.0611067476241625 | XP_062531138.1,<br>XP_062531136.1,<br>XP_062531135.1,<br>XP_062531137.1,<br>XP_062531134.1,<br>XP_012545264.2 | uncharacterized protein LOC101745759 isoform X2<br>uncharacterized protein LOC101745759 isoform X1<br>uncharacterized protein LOC101745759 isoform X1<br>uncharacterized protein LOC101745759 isoform X1<br>uncharacterized protein LOC101745759 isoform X1<br>uncharacterized protein LOC101745759 isoform X1 |
| LOC101740082 | 29995.4126011658 | 1.48633421525081 | 0.0629989171166272 | XP_037869538.1,<br>XP_037869539.1,<br>XP_012547623.2,<br>XP_012547623.2,<br>XP_037869545.1, | sericin 1 |

| | | | | | |
|---|---|---|---|---|---|
| | | | | XP_037869558.1, XP_037869540.1, XP_062526808.1, XP_021205340.2, XP_062526800.1, XP_062526807.1, XP_062526810.1, XP_062526802.1, XP_062526811.1, XP_037869541.1, XP_021205333.2, XP_037869548.1, XP_037869549.1, XP_021205342.2, XP_037869546.1, XP_037869550.1, XP_062526803.1, XP_021205348.2, XP_037869551.1, XP_037869552.1, XP_037869553.1, XP_037869556.1, XP_037869555.1, XP_062526805.1, XP_062526806.1, XP_037869554.1, XP_037869559.1, NP_001037506.2 | |
| LOC134199281 | 46.1213185889463 | -1.34087599344747 | 0.0632632866632986 | XP_062525756.1 | uncharacterized protein LOC134199281 |
| LOC101741740 | 124.144152984506 | -1.36260087045208 | 0.0638572788325833 | XP_004932779.1 | SET and MYND domain-containing protein 4 |
| LOC134200653 | 13.7134569359426 | 1.69884630286007 | 0.0648913705308018 | | uncharacterized protein LOC134200653 |
| LOC101745061 | 99.793998827565 | 1.03880327320636 | 0.0670572148051314 | XP_004922061.3 | NAD-dependent protein deacylase sirtuin-6 |

| | | | | | |
|---|---|---|---|---|---|
| LOC101743433 | 291.597792367243 | 2.05056564599028 | 0.0678189639096714 | XP_012552375.2, XP_062532658.1 | uncharacterized protein LOC101743433 isoform X2, uncharacterized protein LOC101743433 isoform X1 |
| LOC119629447 | 12.6450129094471 | -1.54291842344148 | 0.0681961138801844 | | 5S ribosomal RNA |
| LOC101743815 | 73.356217457276 | -1.28707086701085 | 0.0686678465542827 | XP_004923169.1 | uncharacterized protein LOC101743815 |
| LOC101738158 | 44.1701183078862 | 1.33691044054148 | 0.0694459080492506 | XP_062529130.1, XP_004922782.1 | coiled-coil domain-containing protein 63 |
| LOC101746928 | 134.503610549079 | -1.22785966616621 | 0.069795139310091 | XP_004921652.1 | 15-hydroxyprostaglandin dehydrogenase [NAD(+)] |
| LOC101736650 | 66.0538457716172 | 1.99957557981112 | 0.0702166342516859 | XP_037876747.1, XP_037876746.1 | uncharacterized protein LOC101736650 isoform X2, uncharacterized protein LOC101736650 isoform X1 |
| LOC119629526 | 39.7384273174173 | -1.22776595893893 | 0.0703927602983986 | | U12 minor spliceosomal RNA |
| LOC119630015 | 114.199571739266 | -1.64018221958856 | 0.0703945135575716 | XP_037873758.1 | magnetosome-associated protein MamJ |
| LOC134199182 | 17.0534182194163 | -1.07229270864482 | 0.0709748916843385 | | uncharacterized LOC134199182 |
| LOC101745296 | 29.8158344437763 | 1.62711822180726 | 0.0751196797214374 | XP_004929822.2 | JNK-interacting protein Aplip1 |
| LOC105842367 | 488.348649376963 | -1.42737144892439 | 0.0751452173147303 | XP_012550362.2 | uncharacterized protein LOC105842367 |
| LOC692922 | 37.1185743169383 | 1.02571653947175 | 0.0751689833158014 | NP_001040230.1 | adenylate cyclase |
| LOC105841445 | 53.4298991504349 | 2.22956563112465 | 0.0756687093505554 | XP_062526804.1, XP_062526809.1, XP_062526801.1 | uncharacterized protein LOC105841445 isoform X1, uncharacterized protein LOC105841445 isoform X2, uncharacterized protein LOC105841445 isoform X1 |
| CYP 340.00 | 55.1226476904209 | 1.36763649557707 | 0.0770428042222588 | | |
| LOC105841321 | 19.8010124823893 | -2.33876742984214 | 0.0774672053577769 | XP_037875404.1 | uncharacterized LOC105841321 |
| LOC101739125 | 278.062567775863 | 1.34677072808843 | 0.0781467712883543 | XP_062533049.1, XP_004926666.2 | Alaserpin isoform X2, alaserpin isoform X1 |
| LOC101736547 | 281.454753565818 | 1.31885822251908 | 0.078301011347601 | XP_037869998.1, XP_021207244.1, XP_021207245.1 | gamma-glutamyl hydrolase A isoform X1 gamma-glutamyl hydrolase A isoform X2 gamma-glutamyl hydrolase A isoform X2 |
| LOC119628559 | 23041.5265473525 | 1.88895778499032 | 0.0801399133954935 | | uncharacterized protein LOC119628559 |
| LOC100101209 | 2040.1278035986 | 1.15553839169542 | 0.0814800794804629 | XP_012544978.2, XP_037875828.1, XP_012544980.2, XP_062531901.1, XP_037875829.1 | double-stranded RNA-specific editase Adar isoform X1, double-stranded RNA-specific editase 1 isoform X2, double-stranded RNA-specific editase 1 isoform X3, double-stranded RNA-specific editase 1 isoform X3, double-stranded RNA-specific editase 1 isoform X4 |
| LOC134199656 | 11.7176240673627 | 1.36768348265182 | 0.082870861916679 | XP_062527038.1 | uncharacterized protein LOC134199656 |
| LOC119629864 | 84.0336768717354 | 1.82961011739399 | 0.0847560751434482 | XP_037873251.1 | NPC intracellular cholesterol transporter 1 homolog 1b |

| | | | | | |
|---|---|---|---|---|---|
| Cyce | 108.869305380789 | 1.01681208181241 | 0.0850080960526269 | NP_001188506.1, XP_012545852.1 | cyclin E |
| LOC134199489 | 190.62783834337 | 1.39055631910336 | 0.0858855841517221 | | uncharacterized protein LOC134199489 |
| LOC134201028 | 14.5886779833302 | -1.25268969798733 | 0.0870844231768207 | | uncharacterized protein LOC134201028 |
| LOC119629649 | 315.908612819731 | -1.1905943355533 | 0.0872344557090954 | | U5 spliceosomal RNA |
| LOC101736651 | 305.504498033449 | 1.06568176922517 | 0.0883620062678849 | XP_037871451.1, XP_004923797.2 | GPI mannosyltransferase 3 |
| LOC101742639 | 13.4120344123591 | 1.32471884020413 | 0.0914799614902256 | XP_037867651.1, XP_037867652.1 | high-affinity choline transporter 1 |
| LOC101742146 | 17.4723593116092 | 1.62015883246518 | 0.0914799614902256 | XP_004923692.2 | membrane-bound alkaline phosphatase-like |
| LOC105841810 | 20.9836173405421 | 1.09001071994732 | 0.0921565626267202 | XP_012546801.1 | myb-like protein X |
| LOC105842299 | 114.95364392898 | 1.25892211435075 | 0.0924731313504484 | XP_062530074.1, XP_062530073.1, XP_062530072.1, XP_062530071.1 | meckelin |
| LOC101738735 | 59.8799242721851 | -1.85322527320911 | 0.0939601890719516 | XP_004925059.1 | myogenesis-regulating glycosidase |
| LOC101735447 | 9821.32283826755 | 2.28262485669233 | 0.0947716352065013 | XP_012550392.2, XP_062530135.1, XP_062530134.1, NP_001266309.1 | low molecular mass 30 kDa lipoprotein 19G1-like isoform X2 low molecular mass 30 kDa lipoprotein 19G1-like isoform X2 low molecular mass 30 kDa lipoprotein 19G1-like isoform X1 low molecular mass 30 kDa lipoprotein 19G1-like precursor |
| LOC134201634 | 24.0521735508606 | -1.03891577694612 | 0.0950613701598367 | XP_062532854.1 | uncharacterized protein LOC134201634 |
| Sxl | 1674.95187329196 | 1.78078482661617 | 0.095633545175203 | NP_001036780.1, NP_001166854.1, XP_012548066.2 | sex-lethal isoform L, sex-lethal isoform S, sex-lethal isoform X1 |
| LOC100862826 | 18.380292875736 | 2.44367874827898 | 0.0961283638007018 | NP_001243976.1 | atonal |
| LOC101743716 | 751.881431623234 | 1.29406387751767 | 0.096460547252266 | XP_037872880.1 | uncharacterized PE-PGRS family protein PE_PGRS20 |
| LOC134199191 | 130.817052485601 | 1.82509486768009 | 0.0967681495291598 | | uncharacterized protein LOC134199191 |
| LOC134200161 | 16.4637678253775 | 2.02324567794958 | 0.0967681495291598 | | uncharacterized protein LOC134200161 |
| LOC119628463 | 14.9613874511356 | -2.27306351658784 | 0.0972739469123437 | XP_037866602.1 | uncharacterized protein LOC119628463 |
| LOC100302610 | 1036.25775025412 | 1.33521007992006 | 0.0972739469123437 | XP_062532153.1, XP_062532152.1, XP_062532154.1 | prosperohomeobox protein prospero isoform X1, prosperohomeobox protein prospero isoform X1, prosperohomeobox protein prospero isoform X2 |

| | | | | | |
|---|---|---|---|---|---|
| LOC101738468 | 1101.93348534172 | 1.48265452271752 | 0.0973454242030114 | XP_012552862.1, XP_012552863.1 | general transcription factor IIF subunit 1 isoform X1, general transcription factor IIF subunit 1 isoform X2 |
| LOC101740217 | 173.819692882918 | -1.02500818185932 | 0.097550590883087 | XP_004922032.1 | TBC1 domain family member 16 |
| LOC119629612 | 133.10448670662 | 1.05893231121482 | 0.0987762103888099 | XP_037872012.1 | uncharacterized protein LOC119629612 |
