# Supplementary material for: Transcriptomic Profiling Provides Insight into the Molecular Basis of Heterosis in Philippine-Reared Bombyx mori Hybrids
Source: Insects. 2025 Feb 26;16(3):243. doi: 10.3390/insects16030243 (PMC11942671; doi:10.3390/insects16030243)
Supplement: Supplementary file 1 [file insects-16-00243-s001.zip › Table S6 - DEGs in CN144 (Reference) vs NC144.pdf]

Table S6. Differentially expressed genes between the two Philippine-reared *Bombyx mori* hybrids CN144 and NC144 that fall within the threshold set at padj<0.1 and |log2FoldChange|>1, as determined using DESeq2.

Gene ID	normalized mean exp	log2 FoldChange	padj	Protein ID	description
LOC101747111	2406.6682905122	2.33135135506646	2.18620459313705E-20	XP_021202220.2	zinc finger MIZ domain-containing protein 1
LOC101743057	6777.59842196569	-1.88583812949687	1.47504903676553E-10	XP_037872717.1,<br>XP_037872716.1	uncharacterized protein LOC101743057 isoform X2<br>uncharacterized protein LOC101743057 isoform X1
LOC101742562	398.66224006976	4.17672878451055	1.47504903676553E-10	XP_021207470.1	homeobox protein 2
LOC110385547	219988.058420422	-3.06502487284164	1.26525644154298E-09	XP_037870877.1,<br>XP_037870879.1,<br>XP_037870875.1	exportin-5
LOC101745228	41174.2643688724	-3.33025884251959	1.87213641663997E-09	XP_004926205.1	transmembrane protein 209
LOC101741424	7523.22807111357	-2.54834262818374	2.4555781753478E-09	XP_004924228.3	peroxidase
LOC105842720	335.224492055999	-2.24925934445861	2.56653705889023E-09	XP_062526770.1,<br>XP_062526769.1,<br>XP_062526771.1,<br>XP_062526766.1,<br>XP_062526764.1,<br>XP_062526763.1,<br>XP_062526762.1,<br>XP_062526768.1,<br>XP_062526765.1,<br>XP_037869143.1,<br>XP_062526767.1	spidroin-1 isoform X10<br>spidroin-1 isoform X9<br>glycine-rich protein 1 isoform X11<br>uncharacterized protein LOC105842720 isoform X5<br>keratin, type I cytoskeletal 9 isoform X3<br>keratin, type I cytoskeletal 9 isoform X2<br>translation initiation factor IF-2 isoform X1<br>uncharacterized protein LOC105842720 isoform X8<br>translation initiation factor IF-2 isoform X4<br><br>uncharacterized protein LOC105842720 isoform X7<br>translation initiation factor IF-2 isoform X6
LOC101736123	4145.53483858992	-2.14857256167573	9.89135922086932E-09	XP_004922768.2	glucose dehydrogenase [FAD, quinone]
LOC101746496	8940.2039150259	-3.29641212786144	1.68253647360673E-08	XP_012551216.2	probable RNA-binding protein 46
LOC101739982	1944.02435320314	-1.81926631617303	6.2297910916552E-08	XP_062525342.1,<br>XP_062525341.1,<br>XP_037867855.1,<br>XP_037867856.1	chromatin-remodeling ATPase INO80
LOC101737252	42328.4893986436	-3.42553343465496	6.2297910916552E-08	XP_004926924.2,	neuronal acetylcholine receptor subunit beta-4 isoform X1<br>neuronal acetylcholine receptor subunit beta-4 isoform X2

				XP_062527576.1	
LOC101739693	9006.04805852403	-2.44850728019989	1.13578769589897E-07	XP_004928906.1, XP_021205389.1	jmjC domain-containing histone demethylation protein 1 isoform X1, jmjC domain-containing histone demethylation protein 1 isoform X2
LOC101745749	965.957296597355	-2.56356883264431	2.82847370945101E-07	XP_037873090.2	SLIT-ROBO Rho GTPase-activating protein 3
LOC101746347	4611.25444519914	-2.41787938895575	3.22310946857659E-07	XP_037866526.1	laminin subunit alpha lam-3
LOC101737089	388120.766878796	-3.82961245652929	3.28326893264386E-07	XP_062525199.1, XP_037867524.1, XP_037867526.1, XP_037867523.1, XP_037867525.1, XP_037867522.1	RNA-binding protein FXR1 isoform X6 RNA-binding protein FXR1 isoform X3 RNA-binding protein FXR1 isoform X5 RNA-binding protein FXR1 isoform X2 RNA-binding protein FXR1 isoform X4 RNA-binding protein FXR1 isoform X1
LOC101738350	4254.08822395275	-3.53756493964132	4.93888117214922E-07	XP_037866593.1, XP_062524064.1	sodium bicarbonate cotransporter 3 isoform X2 sodium bicarbonate cotransporter 3 isoform X1
LOC101747200	3671.24043021133	-1.75810886765778	8.6303296709191E-07	XP_021209198.1, XP_021209200.1, XP_021209199.1, XP_021209197.1	protein unc-79 homolog isoform X2 protein unc-79 homolog isoform X4 protein unc-79 homolog isoform X3 protein unc-79 homolog isoform X1
LOC101743940	49819.3010457905	-2.97794319037706	8.6303296709191E-07	XP_062528562.1, XP_004928482.1	EF-hand domain-containing protein 1
LOC101742111	34409.7739612354	-3.20472929404104	8.69836564497093E-07	XP_037872285.1	proteoglycan 4
LOC119630671	85.415846780449	3.65301342081918	1.43791288613137E-06	XP_037876693.2	52 kDa repressor of the inhibitor of the protein kinase
LOC101742916	50850.4053955374	-2.10996481458835	2.54023505398287E-06	XP_037872739.1	RING finger protein unkempt
LOC101739974	1616.27268920317	-2.74254953627432	3.67477610957076E-06	XP_037870327.1, XP_021205742.2, XP_037870328.1	histone acetyltransferase KAT7 isoform X1 histone acetyltransferase KAT7 isoform X3 histone acetyltransferase KAT7 isoform X2
LOC110386833	1147.48083711097	-1.48361288204412	4.75510399260897E-06	XP_037872239.1	PHD finger protein 12
LOC105842669	1418.40578484788	-2.62982134425258	4.75510399260897E-06	XP_037872687.1	uncharacterized protein LOC105842669
LOC101743230	2558.66090147099	-2.73890338151454	5.71518424629087E-06	XP_037877747.1	beclin 1-associated autophagy-related key regulator
LOC134201495	30976.6492529945	-2.52669760079617	5.71518424629087E-06		uncharacterized protein LOC134201495
LOC101740010	75846.8137355492	-2.86288420258085	7.56058044366138E-06	XP_021204679.1, XP_004927938.1	proton channel OtopLc isoform X1, proton channel OtopLc isoform X2

LOC101738201	70106.4355437657	-2.62078201665973	7.56058044366138E-06	XP_037872091.1,<br>XP_037872097.1,<br>XP_037872096.1,<br>XP_037872094.1,<br>XP_037872092.1,<br>XP_037872095.1	protein ECT2 isoform X1<br>protein ECT2 isoform X6<br>protein ECT2 isoform X5<br>protein ECT2 isoform X3<br>protein ECT2 isoform X2<br>protein ECT2 isoform X4
LOC134200953	108.739741097574	-3.68046056124035	7.93762961610706E-06		uncharacterized protein LOC134200953
LOC101735698	183.964288511506	-1.75050267218705	8.04174542268943E-06	XP_062529559.1,<br>XP_037873141.1,<br>XP_037873140.1,<br>XP_037873138.1,<br>XP_062529557.1	probable tubulin polyglutamylase ttll-15 isoform X2<br>probable tubulin polyglutamylase ttll-15 isoform X4<br>probable tubulin polyglutamylase ttll-15 isoform X3<br>probable tubulin polyglutamylase ttll-15 isoform X1<br>probable tubulin polyglutamylase ttll-15 isoform X2
LOC101739226	11745.5692107165	-1.83690300434286	1.10275233355169E-05	XP_062530444.1,<br>XP_062530442.1,<br>XP_062530441.1,<br>XP_062530443.1	prolow-density lipoprotein receptor-related protein 1 isoform X4, low-density lipoprotein receptor-related protein 1 isoform X2, low-density lipoprotein receptor-related protein 1 isoform X1, low-density lipoprotein receptor-related protein 1 isoform X3
LOC101739770	2201.38491283753	-3.61467481365967	1.19397441863635E-05	XP_004922516.1	forkhead box protein D5
LOC101737102	8015.80191523323	-3.39752718660997	1.34262012604569E-05	XP_021203257.2,<br>XP_021203255.2,<br>XP_004925601.2	WD repeat-containing and planar cell polarity effector protein fritz isoform X3<br>WD repeat-containing and planar cell polarity effector protein fritz isoform X1<br>WD repeat-containing and planar cell polarity effector protein fritz isoform X2
LOC101744498	34453.47267107	-1.78341938720058	1.3448943726243E-05	XP_062528206.1,<br>XP_062528205.1,<br>XP_062528204.1,<br>XP_062528203.1	neurobeachin isoform X4<br>neurobeachin isoform X3<br>neurobeachin isoform X2<br>neurobeachin isoform X1
LOC134199088	171.621379814334	-2.93937827194295	1.55121445127041E-05		uncharacterized LOC134199088
LOC134201467	1983.69834603284	-3.24700070240536	1.55668545826329E-05	XP_062532251.1	actin nucleation-promoting factor WASL-like
LOC101743536	17010.1283727439	-1.84559787091676	1.58962632403794E-05	XP_021203316.2,<br>XP_037874530.1,<br>XP_021203313.2	protein fem-1 homolog CG6966 isoform X2<br>protein fem-1 homolog CG6966 isoform X1<br>protein fem-1 homolog CG6966 isoform X1

LOC101736279	153.634242899839	-2.02224954090839	1.82019876272172E-05	XP_004928427.1	organic solute transporter alpha-like protein
LOC101735795	6302.38006994531	-2.70085492236307	2.06954859260405E-05	XP_037871036.1,<br>XP_037871031.1,<br>XP_037871035.1,<br>XP_037871034.1,<br>XP_037871026.1,<br>XP_037871027.1,<br>XP_037871032.1,<br>XP_037871033.1,<br>XP_037871037.1,<br>XP_062527923.1,<br><br>XP_037871029.1,<br>XP_037871030.1,<br>XP_037871028.1	MOB kinase activator-like 2 isoform X9<br>MOB kinase activator-like 2 isoform X7<br>MOB kinase activator-like 2 isoform X8<br>MOB kinase activator-like 2 isoform X8<br>MOB kinase activator-like 2 isoform X2<br>MOB kinase activator-like 2 isoform X3<br>MOB kinase activator-like 2 isoform X7<br>MOB kinase activator-like 2 isoform X7<br>MOB kinase activator-like 2 isoform X7<br>probable RNA-directed DNA polymerase from transposon BS isoform X1<br>MOB kinase activator-like 2 isoform X5<br>MOB kinase activator-like 2 isoform X6<br>MOB kinase activator-like 2 isoform X4
LOC134201654	823.643366434671	-4.90263306181904	2.23546016630071E-05	XP_062532881.1	uncharacterized protein LOC134201654
LOC101736421	415064.113571183	-4.64163428031321	2.60327882436114E-05	XP_037874502.1,<br>XP_037874486.1,<br>XP_037874497.1,<br>XP_037874494.1,<br>XP_062531996.1,<br>XP_037874506.1,<br>XP_062531994.1,<br>XP_037874498.1	protein split ends isoform X5<br>protein split ends isoform X1<br>protein split ends isoform X3<br>protein split ends isoform X2<br>protein split ends isoform X8<br>protein split ends isoform X7<br>protein split ends isoform X6<br>protein split ends isoform X4
LOC134201689	820.455027976195	-4.77587106697207	2.60327882436114E-05	XP_062532914.1	Uncharacterized protein LOC134201689
LOC101745742	2202.20780332897	-2.90403474049509	2.73832759147694E-05	XP_004933010.1,<br>XP_062532249.1	shootin-1
LOC101742624	951426.938204232	-3.01699118515038	2.99481043933982E-05	XP_062530235.1,<br>XP_062530274.1,<br>XP_037873880.1,<br>XP_037873876.1,<br>XP_062530276.1,<br>XP_062530275.1,	uncharacterized protein LOC101742624 isoform X6<br>dystonin isoform X46<br>uncharacterized protein LOC101742624 isoform X12<br>uncharacterized protein LOC101742624 isoform X5<br>dystonin isoform X48<br>dystonin isoform X47

				XP_062530273.1,	dystonin isoform X45
				XP_062530234.1,	uncharacterized protein LOC101742624 isoform X4,
				XP_062530232.1,	uncharacterized protein LOC101742624 isoform X2,
				XP_062530265.1,	uncharacterized protein LOC101742624 isoform X36,
				XP_062530253.1,	uncharacterized protein LOC101742624 isoform X25,
				XP_062530243.1,	uncharacterized protein LOC101742624 isoform X15,
				XP_062530261.1,	uncharacterized protein LOC101742624 isoform X33,
				XP_062530245.1,	uncharacterized protein LOC101742624 isoform X17,
				XP_062530238.1,	uncharacterized protein LOC101742624 isoform X9,
				XP_062530244.1,	uncharacterized protein LOC101742624 isoform X16,
				XP_062530242.1,	uncharacterized protein LOC101742624 isoform X14,
				XP_062530240.1,	uncharacterized protein LOC101742624 isoform X11,
				XP_062530233.1,	uncharacterized protein LOC101742624 isoform X3,
				XP_062530239.1,	uncharacterized protein LOC101742624 isoform X10,
				XP_062530257.1,	uncharacterized protein LOC101742624 isoform X29,
				XP_062530251.1,	uncharacterized protein LOC101742624 isoform X23,
				XP_062530247.1,	uncharacterized protein LOC101742624 isoform X19,
				XP_062530236.1,	uncharacterized protein LOC101742624 isoform X7,
				XP_062530254.1,	uncharacterized protein LOC101742624 isoform X26,
				XP_062530249.1,	uncharacterized protein LOC101742624 isoform X21,
				XP_062530246.1,	uncharacterized protein LOC101742624 isoform X18,
				XP_037873869.1,	uncharacterized protein LOC101742624 isoform X1,
				XP_062530256.1,	uncharacterized protein LOC101742624 isoform X28,
				XP_062530250.1,	uncharacterized protein LOC101742624 isoform X22,
				XP_062530241.1,	uncharacterized protein LOC101742624 isoform X13,
				XP_062530252.1,	uncharacterized protein LOC101742624 isoform X24,
				XP_062530248.1,	uncharacterized protein LOC101742624 isoform X20,
				XP_062530237.1,	uncharacterized protein LOC101742624 isoform X8,
				XP_062530266.1,	uncharacterized protein LOC101742624 isoform X37,
				XP_062530270.1,	uncharacterized protein LOC101742624 isoform X41,
				XP_062530269.1,	uncharacterized protein LOC101742624 isoform X40,
				XP_062530267.1,	uncharacterized protein LOC101742624 isoform X38,
				XP_062530263.1,	uncharacterized protein LOC101742624 isoform X35,
				XP_062530268.1,	uncharacterized protein LOC101742624 isoform X39,

				XP_062530262.1,<br>XP_062530259.1,<br>XP_062530255.1,<br>XP_062530260.1,<br>XP_062530258.1,<br>XP_062530271.1,<br>XP_062530272.1,<br>XP_062530231.1,<br>XP_037873892.1,<br>XP_037873891.1	uncharacterized protein LOC101742624 isoform X34,<br>uncharacterized protein LOC101742624 isoform X31,<br>uncharacterized protein LOC101742624 isoform X27,<br>uncharacterized protein LOC101742624 isoform X32,<br>uncharacterized protein LOC101742624 isoform X30,<br>microtubule-actin cross-linking factor 1 isoform X43,<br>microtubule-actin cross-linking factor 1 isoform X44,<br>uncharacterized protein LOC101742624 isoform X1,<br>uncharacterized protein LOC101742624 isoform X42,<br>uncharacterized protein LOC101742624 isoform X42
LOC101738544	1045.30496908611	-1.57838553492591	3.91105230242135E-05	XP_012550223.1,<br>XP_004931265.1	zinc finger protein pita isoform X1<br>zinc finger and BTB domain-containing protein 17 isoform X2,
Nanoso	4711.12689876986	-2.68241960847534	3.91105230242135E-05	NP_001093314.1,<br>XP_062528227.1,<br>XP_012544345.1,<br>XP_062528226.1	zinc finger protein NANOS-O<br>zinc finger protein NANOS-O, isoform X2<br>zinc finger protein NANOS-O, isoform X1<br>zinc finger protein NANOS-O, isoform X2
LOC101745046	17477.9795924395	-3.86848247188727	4.36833712655427E-05	XP_037866520.1,<br>XP_062533112.1,<br>XP_037866519.1	conserved oligomeric Golgi complex subunit 4 isoform X1,<br>conserved oligomeric Golgi complex subunit 4 isoform X2,<br>conserved oligomeric Golgi complex subunit 4 isoform X1
LOC101735695	40864.7153335645	-2.44618496924757	4.36833712655427E-05	XP_062524202.1,<br>XP_037877805.1,<br>XP_062524201.1	sodium leak channel NALCN isoform X3<br>sodium leak channel NALCN isoform X2<br>sodium leak channel NALCN isoform X1
LOC101735452	3355.99695936668	-2.74911123077958	4.64501878783784E-05	XP_062531727.1,<br>XP_021209115.1,<br>XP_021209114.1,<br>XP_021209112.1,<br>XP_037875712.1,<br>XP_012552943.1,<br>XP_021209113.1,<br>XP_021209110.1	protein enabled isoform X6<br>protein enabled isoform X5<br>protein enabled isoform X4<br>protein enabled isoform X3<br>protein enabled isoform X1<br>protein enabled isoform X1<br>protein enabled isoform X2<br>protein enabled isoform X1

LOC101745478	2963.99420631452	-2.90752817282201	5.88513981667997E-05	XP_037875325.1, XP_037875326.1, XP_037875328.1	molybdenum cofactor biosynthesis protein 1 isoform X1, molybdenum cofactor biosynthesis protein 1 isoform X2, molybdenum cofactor biosynthesis protein 1 isoform X3
LOC101743221	31076.4240944938	-3.38929582320344	6.44753765143901E-05	XP_012545781.1, XP_012545779.1, XP_062532341.1	inositol polyphosphate-5-phosphatase A isoform X3, inositol polyphosphate-5-phosphatase A isoform X1, uncharacterized protein LOC101743221 isoform X2
LOC110385711	175.871001279503	-2.29211671086071	7.22467486350763E-05	XP_021205610.1	putative uncharacterized protein DDB_G0279653
LOC101742772	12252.7535578865	-1.95071367166999	7.22467486350763E-05	XP_012543788.3, XP_012543787.3	uncharacterized protein LOC101742772
LOC101745670	159.748996916653	-2.12394431784734	7.22467486350763E-05	XP_004926346.1, XP_012545976.1	uncharacterized protein LOC101745670 isoform X2, uncharacterized protein LOC101745670 isoform X1
CPR60	79.1943255302961	-9.48881146452428	7.51257913879218E-05	NP_001166697.1	cuticular protein RR-2 motif 60
LOC101740344	1397.10705024671	-2.40156663668099	7.58035542156512E-05	XP_004933707.3, XP_021208635.1	clusterin associated protein 1
LOC101744797	3517.6056753097	-3.04945331931242	9.76340542576604E-05	XP_062524486.1, XP_021204623.2	integrator complex subunit 9 isoform X2 integrator complex subunit 9 isoform X1
LOC101739623	5927.13769107253	-3.11475113679385	0.00010439005132879	XP_021208538.2, XP_012551644.2, XP_062526724.1	E3 ubiquitin-protein ligase MGRN1 isoform X1 E3 ubiquitin-protein ligase MGRN1 isoform X2 E3 ubiquitin-protein ligase MGRN1 isoform X3
LOC105842130	92.0073268776603	-1.85548110354906	0.00010439005132879	XP_037874433.1	voltage-dependent calcium channel subunit alpha-2/delta-3
CPR104	1260.8226981055	-2.54324465990257	0.000117137307517607	NP_001036854.1	cuticular protein RR-2 motif 104
LOC101736050	7810.074193793	-1.39042296965173	0.000117398224024041	XP_021204783.2, XP_037870931.1, XP_037870930.1	eukaryotic translation initiation factor 4 gamma 2 isoform X1, eukaryotic translation initiation factor 4 gamma 2 isoform X2, eukaryotic translation initiation factor 4 gamma 2 isoform X1
LOC101746770	1475.48068771866	-3.39355561238077	0.000117398224024041	XP_037872371.1, XP_037872366.1, XP_021205486.1, XP_037872368.1, XP_037872370.1, XP_012548225.1,	RIMS-binding protein 2 isoform X13 RIMS-binding protein 2 isoform X1 peripheral-type benzodiazepine receptor-associated protein 1 isoform X2 RIMS-binding protein 2 isoform X4 RIMS-binding protein 2 isoform X8 peripheral-type benzodiazepine receptor-associated protein 1 isoform X6

				XP_021205488.1,<br>XP_012548223.1,<br>XP_012548227.1,<br>XP_037872367.1,<br>XP_062529006.1,<br>XP_012548228.1,<br>XP_062529007.1	peripheral-type benzodiazepine receptor-associated protein 1 isoform X7<br>RIMS-binding protein 2 isoform X5<br>peripheral-type benzodiazepine receptor-associated protein 1 isoform X10<br>peripheral-type benzodiazepine receptor-associated protein 1 isoform X3<br>peripheral-type benzodiazepine receptor-associated protein 1 isoform X9<br>peripheral-type benzodiazepine receptor-associated protein 1 isoform X11<br>peripheral-type benzodiazepine receptor-associated protein 1 isoform X12
CPR102	2501.63260952374	-2.55385898386452	0.000117398224024041	NP_001166664.1,<br>XP_062530797.1	cuticular protein RR-2 motif 102 precursor<br>cuticular protein RR-2 motif 102 isoform X1
LOC101739647	1866.79679604388	-0.9580309182309	0.000126892181759154	XP_004930050.1,<br>XP_062524770.1,<br>XP_021206285.1,<br>XP_062524769.1	E3 ubiquitin-protein ligase Topors
LOC101742572	4051.27413935698	-2.71840708647758	0.000126892181759154	XP_037870319.1,<br>XP_062527319.1,<br>XP_062527318.1	apoptosis-resistant E3 ubiquitin protein ligase 1 isoform X1,<br>apoptosis-resistant E3 ubiquitin protein ligase 1 isoform X3,<br>apoptosis-resistant E3 ubiquitin protein ligase 1 isoform X2
CPR103	1244.52503765474	-2.58235895949303	0.000134558730359815	NP_001036855.1	cuticular protein RR-2 motif 103
LOC101738096	1755.29345627151	-2.51241275023846	0.000136206798175178	XP_004931378.1	Another transcription unit
LOC101736510	4742.00347461841	-1.76232372544194	0.000146420688195658	XP_021208198.2,<br>XP_012552320.2	ubiquitin carboxyl-terminal hydrolase 32 isoform X2<br>ubiquitin carboxyl-terminal hydrolase 32 isoform x1
LOC101744691	15975.1229999472	-2.66937252555322	0.000156808535980824	XP_004923175.2	heterogeneous nuclear ribonucleoprotein U-like protein 1
HSP70	7146.59783198894	1.80561170735753	0.000176543636927549	XP_012547740.1	heat shock protein 70
LOC101735737	1344.09609288381	-1.82801799491941	0.0001886286446836	XP_037868300.1	ankyrin repeat domain-containing protein 13D
LOC105842535	160.579438942235	-1.41906861113041	0.000198821320320305	XP_062532270.1,<br>XP_021208143.2,<br>XP_037876537.1	uncharacterized protein LOC105842535 isoform X2,<br>uncharacterized protein LOC105842535 isoform X2,<br>uncharacterized protein LOC105842535 isoform X1
LOC101737947	66.0622683739783	-1.83769628484853	0.000199795760338078	XP_004925821.1	Uncharacterized protein LOC101737947

LOC105842191	1348.5576704848	-4.22463317890653	0.000199795760338078		uncharacterized LOC105842191
LOC134198715	34.0293854602916	-4.14760777581431	0.000199795760338078		uncharacterized LOC134198715
_Bre4	38309.7725199757	-2.1606308985385	0.000206389427325247	NP_001243988.1, XP_012545894.1, XP_012545895.1	Glycosyltransferase precursor, Glycosyltransferase isoform X1 Glycosyltransferase isoform X1
LOC101745604	9577.54492051839	-2.11378985636979	0.000206389427325247	XP_004924245.1, XP_004924244.1, XP_004924246.1	DNA repair protein RAD52 homolog isoform X2 uncharacterized protein LOC101745604 isoform X1 DNA repair protein RAD52 homolog isoform X3
LOC134201556	643.471140231256	-3.93157068721831	0.000206389427325247	XP_062532663.1	basic proline-rich protein-like
LOC101739743	566.304782839672	-2.93823799830297	0.000206963641142965	XP_004928993.2	uncharacterized protein LOC101739743
LOC134199681	475.754056253068	-3.1848732023437	0.000210029711718554		uncharacterized LOC134199681
LOC101736947	30895.9233835639	-2.68405601532352	0.000210029711718554	XP_004930824.1	uncharacterized protein LOC101736947
LOC105841467	18.3271687860345	-3.04144762679494	0.000210029711718554		uncharacterized LOC105841467
LOC101745344	40.9843936765201	-2.10696948510451	0.000227040134074213	XP_012548339.1	thioredoxin domain-containing protein 3 homolog
LOC101744757	25827.144036529	-2.04175370424675	0.000244672326450715	XP_037872690.1	forkhead box K
LOC119629653	1132.49369511963	-2.68890710050885	0.00033017559016325		small nucleolar RNA SNORD36
LOC105841770	90.8002526929707	-3.27989751255796	0.00034139459366551	XP_037868210.1	uncharacterized protein LOC105841770
STIP	743.387638499431	-1.32794198123144	0.00034139459366551	NP_001091840.1, XP_012550228.2, XP_012550229.2	septin interacting protein 1 septin and tuftelin interacting protein isoform X1 septin and tuftelin interacting protein isoform X2
LOC134199678	1682.59888986202	-5.3164375102137	0.00034523051208085		uncharacterized protein LOC134199678
LOC101736789	846.6157228007	-2.29430389883053	0.000351689892224123	XP_062526666.1, XP_012553001.2, XP_021209174.2	myelin transcription factor 1 isoform X3 myelin transcription factor 1 isoform X1 myelin transcription factor 1 isoform X2
LOC101746167	3826.97272714231	-1.00014933692491	0.000369064236539247	XP_062529970.1, XP_062529965.1, XP_062529974.1, XP_004933414.1, XP_021208400.1, XP_062529973.1, XP_062529963.1, XP_062529967.1, XP_062529966.1,	dystroglycan 1 isoform X3, dystroglycan 1 isoform X1, dystroglycan 1 isoform X6, dystroglycan 1 isoform X3, dystroglycan 1 isoform X2, dystroglycan 1 isoform X4, dystroglycan 1 isoform X1, dystroglycan 1 isoform X1,

				XP_062529971.1, XP_062529968.1, XP_062529972.1, XP_004933416.1, XP_062529975.1, XP_062529969.1, XP_021208401.1, XP_062529964.1	dystroglycan 1 isoform X1, dystroglycan 1 isoform X3, dystroglycan 1 isoform X1, dystroglycan 1 isoform X3, dystroglycan 1 isoform X5, dystroglycan 1 isoform X6, dystroglycan 1 isoform X3, dystroglycan 1 isoform X2, dystroglycan 1 isoform X1
LOC105842604	1865.46580831552	-4.08598599003526	0.00037421259018791	XP_062532742.1	Uncharacterized protein LOC105842604
LOC101735821	121926.93644744	-4.7918928621963	0.000413222392777722	XP_012546957.1, XP_021204471.1	Negative elongation factor A isoform X2 Negative elongation factor A isoform X1
Cyp307a1	1633473.99806759	-3.34687871440064	0.000422406415993435	NP_001104833.1	cytochrome P450, family 307, subfamily a, polypeptide 1
LOC101739744	1508.69685338254	-2.68473833595804	0.000422406415993435	XP_062528975.1, XP_037872375.1, XP_037872378.1, XP_037872377.1	citron rho-interacting kinase isoform X4 citron rho-interacting kinase isoform X1 citron rho-interacting kinase isoform X3 citron rho-interacting kinase isoform X2
LOC101735945	52540.2291661616	-3.89676678593015	0.000445386396087825	XP_037871579.1, XP_062528425.1	probable E3 ubiquitin-protein ligase HERC2 isoform X1 probable E3 ubiquitin-protein ligase HERC2 isoform X2
LOC101739763	291.042125249075	-1.15462033189927	0.000449099534947568	XP_062532741.1, XP_062532740.1, XP_037877163.1, XP_062532739.1	ribosomal protein S6 kinase-like 1 isoform X4 ribosomal protein S6 kinase delta-1 isoform X3 ribosomal protein S6 kinase delta-1 isoform X1 ribosomal protein S6 kinase delta-1 isoform X2
LOC101746180	741.016248568136	-3.49881489928936	0.000453468790167583	XP_037869395.1, XP_037869394.1	guanylate cyclase 32E isoform X2 guanylate cyclase 32E isoform X1
LOC101744215	89.9563391670609	-2.83064580897607	0.000453468790167583	XP_012544067.2, XP_012544066.2	neurexin 1 isoform X1, neurexin 1 isoform X2
LOC101736666	1197.16185937188	-2.92849982817009	0.000457383573904068	XP_037871239.1, XP_037871237.1, XP_062527974.1, XP_062527973.1	protein cramped isoform X2, protein cramped isoform X1 protein cramped isoform X3, protein cramped isoform X3
LOC692743	272274.379106343	-2.17960945681604	0.000490121787645425	NP_001104817.1	hemocytin
LOC101747089	432.607522811928	-3.23961634366337	0.000497849739570624	XP_004931628.1	BAI1-associated protein 3

R2D2	842.813509003894	-2.22197842385613	0.000497849739570624	NP_001182007.1,<br>XP_037870654.1	R2D2 protein isoform X1
LOC134201133	1135.97901999876	-3.02633436157721	0.000497849739570624		uncharacterized LOC134201133
LOC119630444	98.1172008761673	-3.41282998844368	0.000497849739570624		uncharacterized LOC119630444
LOC134199623	222.764538236948	-3.01082373131696	0.000502564822488471	XP_062526873.1,<br>XP_062526874.1	uncharacterized protein LOC134199623
LOC134201261	142.398119530141	-1.22775433932937	0.000512893817259493		uncharacterized LOC134201261
LOC119630545	359.954147102912	-1.66707964862532	0.000512893817259493	XP_037876199.1	uncharacterized protein LOC119630545
LOC101736324	511.770746788078	-1.18388796501507	0.000525689900069105	XP_037870889.1,<br>XP_037870887.1,<br>XP_037870888.1,<br>XP_037870886.1,<br>XP_062527916.1,<br>XP_062527915.1,<br>XP_021204805.1	transient receptor potential-gamma protein isoform X4<br>transient receptor potential-gamma protein isoform X2<br>transient receptor potential-gamma protein isoform X3<br>transient receptor potential-gamma protein isoform X1<br>transient receptor potential-gamma protein isoform X1<br>transient receptor potential-gamma protein isoform X1<br>transient receptor potential-gamma protein isoform X5
LOC101737191	1472.65644782324	-1.17763553396705	0.00052713890967832	XP_004924201.1	Ubiquitin-like activating enzyme 4
InR	19261.6589451421	-3.50129996494838	0.000532463961779646	NP_001037011.1,<br>XP_062524423.1,<br>XP_062524422.1	insulin receptor precursor<br>insulin receptor isoform X2<br>insulin receptor isoform X1
LOC101739938	1790.68619295133	-2.01107924564636	0.000532463961779646	XP_062526760.1,<br>XP_037869142.1,<br>XP_062526761.1	helicase domino isoform X1<br>helicase domino isoform X2<br>helicase domino isoform X3
LOC101737181	4523.23422998279	-1.70670636366186	0.000535029736706111	XP_062526876.1,<br>XP_012548523.2,<br>XP_062526875.1,<br>XP_012548518.2	intersectin-2 isoform X4<br>intersectin-2 isoform X3<br>intersectin-2 isoform X2<br>intersectin-2 isoform X1
LOC134200584	32.6327773082417	-2.89743599739067	0.000535029736706111		uncharacterized LOC134200584
LOC101743627	107157.166783321	-2.72808917105914	0.000535029736706111	XP_037875664.1,<br>XP_037875665.1	EH domain-binding protein 1 isoform X1<br>EH domain-binding protein 1 isoform X2
LOC101747141	118.822471869233	-3.79410913279679	0.000536167738256073	XP_012550554.1,<br>XP_004931839.2	runt-related transcription factor 3 isoform X2,<br>runt-related transcription factor 3 isoform X1
LOC101740583	344.421517295187	-3.71843453202714	0.000544240446450315	XP_021203081.2	chorion peroxidase
LOC110386536	1197482.41761508	-2.63215779460668	0.000545425597396061	XP_037873436.1	uncharacterized protein LOC110386536

LOC101741715	5880.74812787905	-2.64118991718273	0.000554216941364786	XP_037877794.1, XP_062524387.1, XP_037877792.1, XP_037877797.1	glutathione hydrolase 7 isoform X1 glutathione hydrolase 6 isoform X2 glutathione hydrolase 7 isoform X1 glutathione hydrolase 6 isoform X2
LOC134201655	2739.07641612333	-4.06464363692703	0.000554216941364786	XP_062532882.1	uncharacterized protein LOC134201655
LOC101739639	236660.60600077	-2.09000788054446	0.000598744402242375	XP_004926240.1, XP_062524521.1	tubulin gamma-1 chain isoform X1 tubulin gamma-1 chain isoform X2
LOC101743771	2556973.2244118	-2.5617615759062	0.000598744402242375	XP_037871235.1, XP_062528180.1, XP_062528179.1, XP_012543850.1	rho GTPase-activating protein 6 isoform X3 rho GTPase-activating protein 6 isoform X3 uncharacterized protein LOC101743771 isoform X2 rho GTPase-activating protein 6 isoform X1
LOC101735700	85.2928264484833	-3.39416732100545	0.000619372265614261	XP_004929162.1	uncharacterized protein LOC101735700
LOC778495	650.768002822203	-2.21251455263491	0.000632975293858525	NP_001091790.1	candidate olfactory receptor
LOC101747187	2027.15791932584	-1.19991747047946	0.000632975293858525	XP_037869437.1, XP_037869436.1, XP_037869439.1, XP_037869438.1, XP_037869440.1, XP_062526358.1, XP_037869441.1, XP_062526359.1, XP_037869442.1, XP_037869443.1, XP_062526360.1	mediator of RNA polymerase II transcription subunit 13 isoform X1 mediator of RNA polymerase II transcription subunit 13 isoform X1, mediator of RNA polymerase II transcription subunit 13 isoform X3, mediator of RNA polymerase II transcription subunit 13 isoform X2, mediator of RNA polymerase II transcription subunit 13 isoform X4, mediator of RNA polymerase II transcription subunit 13 isoform X5, mediator of RNA polymerase II transcription subunit 13 isoform X6, mediator of RNA polymerase II transcription subunit 13 isoform X8, mediator of RNA polymerase II transcription subunit 13 isoform X7, mediator of RNA polymerase II transcription subunit 13 isoform X9, mediator of RNA polymerase II transcription subunit 13 isoform X10
LOC101739964	723.370744102921	-2.34591525858101	0.000640396800261754	XP_037869706.1, XP_037869704.1, XP_062527270.1, XP_062527269.1, XP_037869703.1	uncharacterized protein LOC101739964 isoform X2, uncharacterized protein LOC101739964 isoform X1, uncharacterized protein LOC101739964 isoform X2, uncharacterized protein LOC101739964 isoform X1, uncharacterized protein LOC101739964 isoform X1

LOC110386645	6776.14933872767	-3.21231040222772	0.000659431419085532	XP_037876429.1, XP_037876436.1	monocarboxylate transporter 4
Borealin	64.152603187882	-2.2035625187089	0.000659431419085532	NP_001266370.1, XP_012545696.1	uncharacterized protein LOC101738843 uncharacterized protein LOC101738843 isoform X1
LOC101747074	671.61394947312	-1.69880407111825	0.000665481319140908	XP_037866802.1, XP_037866803.1, XP_037866805.1, XP_037866806.1	uncharacterized protein LOC101747074 isoform X1, uncharacterized protein LOC101747074 isoform X2, uncharacterized protein LOC101747074 isoform X3, uncharacterized protein LOC101747074 isoform X4
LOC101736187	8490.87152199028	-2.69874401309342	0.000673020735480275	XP_062527913.1, XP_062527912.1, XP_062527910.1, XP_062527914.1, XP_037870925.1	tyrosine-protein phosphatase 10D isoform X3 tyrosine-protein phosphatase 10D isoform X3 tyrosine-protein phosphatase 10D isoform X1 tyrosine-protein phosphatase 10D isoform X4 tyrosine-protein phosphatase 10D isoform X2
LOC101735622	406.407980767937	-2.36887407391592	0.000675850615748013	XP_021207334.2, XP_021207333.2, XP_062532113.1, XP_062532104.1, XP_062532095.1, XP_062532100.1, XP_062532093.1, XP_062532117.1, XP_062532119.1, XP_012550681.2	histone-lysine N-methyltransferase PRDM16 isoform X7 histone-lysine N-methyltransferase PRDM16 isoform X5 histone-lysine N-methyltransferase PRDM16 isoform X6 histone-lysine N-methyltransferase PRDM16 isoform X4 histone-lysine N-methyltransferase PRDM16 isoform X2 histone-lysine N-methyltransferase PRDM16 isoform X3 histone-lysine N-methyltransferase PRDM16 isoform X1 histone-lysine N-methyltransferase PRDM16 isoform X9 histone-lysine N-methyltransferase PRDM16 isoform X10 histone-lysine N-methyltransferase PRDM16 isoform X8
LOC101736444	7550.19927164659	-2.02855287416014	0.000675850615748013	XP_037875618.1, XP_037875617.1	protein slit isoform X2 protein slit isoform X1
UGT10287B	75.6820217383471	-4.66533513453414	0.000709029264808206	NP_001182388.1	UDP-glucosyltransferase
LOC101741856	123.21444522668	-1.64176031504776	0.000709029264808206	XP_004924098.2	uncharacterized protein LOC101741856
LOC134198832	6352.75442798227	-1.69525701923034	0.000743934482126386		uncharacterized LOC134198832
LOC101740626	94.5555561493006	-1.94134968782678	0.000770265147091882	XP_037872157.1, XP_062529089.1, XP_062529090.1	uncharacterized protein LOC101740626 isoform X1, uncharacterized protein LOC101740626 isoform X2, uncharacterized protein LOC101740626 isoform X3
LOC134199221	353.540198304144	-2.38841117635292	0.0008066020188103	XP_062525577.1	lipase 1-like

LOC101737216	13490.1091896267	-2.48562050114811	0.000819830734655719	XP_004930123.1, XP_012549251.1	Uncharacterized protein LOC101737216
LOC110385321	5360.55653763095	-2.77793715400041	0.000819830734655719	XP_062527568.1, XP_037870206.1, XP_062527569.1	lysosomal-trafficking regulator isoform X1 lysosomal-trafficking regulator isoform X2 lysosomal-trafficking regulator isoform X1
LOC101735777	593.552785079147	-1.45565302507132	0.00082472706604095	XP_004924190.1	U1 small nuclear ribonucleoprotein 70 kDa
LOC101746240	319.496939281174	-2.36793251132828	0.000862379159264904	XP_012544310.2	uncharacterized protein LOC101746240
LOC101740211	72288.9989207823	-2.89028245004654	0.000927589274746392	XP_012551556.1	peptide transporter family 1
LOC134201214	93.9989895699995	-3.62046843895059	0.000927589274746392		uncharacterized protein LOC134201214
LOC105842623	5621.19840127251	-1.71847726426375	0.000944482265179119	XP_062524485.1	cell adhesion molecule Dscam2
LOC101740860	34747.6778631888	-3.7936452386357	0.000964257509097064	XP_062526850.1	serine/arginine repetitive matrix protein 1-like
LOC119629129	4281.14588868957	-2.43054585762637	0.000975335824859483	XP_037869946.1, XP_062527008.1	zinc finger protein 91
LOC101740720	63129.9691437525	-2.00137116722769	0.000992471890279993	XP_004924089.2	translation initiation factor IF-2, mitochondrial
LOC692928	165.58695589723	1.17666428635807	0.00104291363680067	NP_001040235.1	uncharacterized protein LOC692928
LOC101745654	1999.57926953299	-2.095006535977	0.00106679215808591	XP_004923310.2	transcription initiation factor TFIID subunit 3
LOC101741294	114.001788579425	-2.46731790771513	0.00107119060524374	XP_037877868.1, XP_021203434.1, XP_021203430.1, XP_062524385.1	uncharacterized protein LOC101741294 isoform X3, uncharacterized protein LOC101741294 isoform X2, uncharacterized protein LOC101741294 isoform X1, uncharacterized protein LOC101741294 isoform X4
LOC101741165	113.294021549504	-2.86872248373486	0.00107119060524374	XP_004929396.1	radial spoke head protein 9 homolog
LOC101738029	2615.58173076018	-2.2578299478685	0.00109657726092566	XP_037871281.1, XP_062528078.1, XP_062528075.1, XP_062528080.1, XP_062528073.1, XP_062528086.1, XP_062528089.1, XP_062528088.1, XP_062528084.1, XP_062528072.1, XP_062528085.1,	AT-rich interactive domain-containing protein 2 isoform X1 AT-rich interactive domain-containing protein 2 isoform X8 AT-rich interactive domain-containing protein 2 isoform X5 AT-rich interactive domain-containing protein 2 isoform X11 AT-rich interactive domain-containing protein 2 isoform X3 AT-rich interactive domain-containing protein 2 isoform X17, AT-rich interactive domain-containing protein 2 isoform X20, AT-rich interactive domain-containing protein 2 isoform X19, AT-rich interactive domain-containing

				XP_037871286.1, XP_062528090.1, XP_062528079.1, XP_062528091.1, XP_062528074.1, XP_062528076.1, XP_062528092.1, XP_062528077.1, XP_062528082.1, XP_062528094.1, XP_062528083.1, XP_062528081.1, XP_062528093.1, XP_062528087.1	protein 2 isoform X15, AT-rich interactive domain-containing protein 2 isoform X2 AT-rich interactive domain-containing protein 2 isoform X16, AT-rich interactive domain-containing protein 2 isoform X9 AT-rich interactive domain-containing protein 2 isoform X21, AT-rich interactive domain-containing protein 2 isoform X10, AT-rich interactive domain-containing protein 2 isoform X22, AT-rich interactive domain-containing protein 2 isoform X4 AT-rich interactive domain-containing protein 2 isoform X6 AT-rich interactive domain-containing protein 2 isoform X23, AT-rich interactive domain-containing protein 2 isoform X7 AT-rich interactive domain-containing protein 2 isoform X13, AT-rich interactive domain-containing protein 2 isoform X25, AT-rich interactive domain-containing protein 2 isoform X14, AT-rich interactive domain-containing protein 2 isoform X12, AT-rich interactive domain-containing protein 2 isoform X24, AT-rich interactive domain-containing protein 2 isoform X18
LOC101738468	1389.13880607065	-2.39429551887375	0.00109657726092566	XP_012552862.1, XP_012552863.1	general transcription factor IIF subunit 1 isoform X1 general transcription factor IIF subunit 1 isoform X2
LOC101740918	72.2281122148361	-2.5914309050939	0.00109657726092566	XP_004925477.1, XP_012545250.1	uncharacterized protein LOC101740918 isoform X2, uncharacterized protein LOC101740918 isoform X1
LOC101735871	258.438907899149	-1.34820618386007	0.00114124248769523	XP_012546046.1	sodium-independent sulfate anion transporter
LOC778508	347523.962366188	-2.48613123679452	0.00115953460039131	NP_001091803.1, XP_062529201.1	putative mitochondrial ribosomal protein putative mitochondrial ribosomal protein isoform X1
LOC101744576	16691.6962060236	-3.49515978848275	0.00120329523160157	XP_012550342.2, XP_062525369.1	myosin-11 isoform X2, myosin-11 isoform X1
LOC101739283	3006.53595253386	-1.87187600324033	0.00121552964607312	XP_062527172.1, XP_062527173.1, XP_062527174.1,	dehydrogenase/reductase SDR family member 4

				XP_004931489.1, XP_062527171.1, XP_062527175.1	
LOC119628349	15666.729416306	-2.24041518010341	0.00132931951387433	XP_062529259.1	protein shuttle craft
LOC101740962	4070.86226294446	-3.08274052830476	0.00132941836009538	XP_012544866.1, XP_012544867.1	zinc finger CCCH domain-containing protein 13
LOC105841761	89.4457007100956	-2.15437410433825	0.00134030191719693	XP_062531008.1	uncharacterized protein LOC105841761
MS	33.4334984391522	-3.03518094136746	0.00134405307433806	XP_012553035.1, NP_001166882.1	myosuppressin, isoform X1
LOC101745732	3408.688281752	-1.49220293809335	0.00134405307433806	XP_062526375.1, XP_021206387.1	E3 ubiquitin-protein ligase Bre1 isoform X2<br>E3 ubiquitin-protein ligase Bre1 isoform X1
LOC101741718	824.706890176753	-2.86720276870966	0.00139247753276425	XP_062531122.1, XP_012545932.1	glucose transporter type 1 isoform X2<br>glucose transporter type 1 isoform X1
LOC134199760	65.2709247258402	-1.7582286832744	0.00139247753276425		uncharacterized protein LOC134199760
LOC101738935	1770.01226078656	-1.82481526440169	0.00140645155936099	XP_037876175.1, XP_037876176.1, XP_062531997.1, XP_037876177.1, XP_037876174.1	integrator complex subunit 1 isoform X2<br>integrator complex subunit 1 isoform X3<br>integrator complex subunit 1 isoform X5<br>integrator complex subunit 1 isoform X4<br>integrator complex subunit 1 isoform X1
LOC101735982	15244.7572318725	-1.85345504758419	0.00146078939772731	XP_062528809.1, XP_062528804.1, XP_062528798.1, XP_062528805.1, XP_062528801.1, XP_062528802.1, XP_062528808.1	transcription factor CP2-like protein 1 isoform X5<br>transcription factor CP2-like protein 1 isoform X3<br>transcription factor CP2-like protein 1 isoform X1<br>transcription factor CP2-like protein 1 isoform X3<br>transcription factor CP2-like protein 1 isoform X1<br>transcription factor CP2-like protein 1 isoform X2<br>transcription factor CP2-like protein 1 isoform X4
LOC101735602	99.3923171197881	-2.37928774452425	0.00150767849665052	XP_062532664.1	paired box protein Pax-5
LOC101743806	954.352172022847	-2.00096648241974	0.00157147310476782	XP_037876488.1	protein Skeletor, isoforms B/C
LOC101736469	388.869991585093	-4.09804007106764	0.00160800362771778	XP_012552956.1, XP_004921663.1	DNA fragmentation factor-related protein 2
LOC110385005	8010.32618708869	-2.63328737576769	0.00163306063835759	XP_062528236.1, XP_037873339.1, XP_062528241.1,	uncharacterized protein LOC110385005 isoform X1,<br>uncharacterized protein LOC110385005 isoform X2,<br>uncharacterized protein LOC110385005 isoform X3,

				XP_062528244.1, XP_062528251.1, XP_037873360.1	uncharacterized protein LOC110385005 isoform X3, uncharacterized protein LOC110385005 isoform X3, uncharacterized protein LOC110385005 isoform X4
LOC100862753	4633.20300033314	-3.21746417799973	0.00164700391523224	XP_037871498.1, XP_037871500.1, XP_037871499.1, XP_037871501.1, XP_037871502.1, XP_037871503.1	mkk7 isoform X1 mkk7 isoform X3 mkk7 isoform X2 mkk7 isoform X4 mkk7 isoform X5 mkk7 isoform X6
LOC119629958	115.559885523357	-3.16713223890063	0.00165105323353585		Uncharacterized LOC119629958
LOC101735876	2094.37000100084	1.33401931729504	0.00165748932753489	XP_004926779.1, XP_004926780.1	aldo-keto reductase AKR2E4-like
LOC101739625	2153.39337026582	-2.79268818863719	0.00165748932753489	XP_004922154.1	zinc finger protein Zpr1
LOC134200302	145.644597392462	-2.32107905521484	0.00168556325253205		uncharacterized LOC134200302
LOC101741950	11915.242193252	-2.69236607893883	0.00169092385940618	XP_012544709.2, XP_062526584.1	protein sidekick
LOC101735723	101.563391720309	-3.31876821080085	0.00169092385940618	XP_062526864.1, XP_062526864.1, XP_062526860.1, XP_021204668.1, XP_062526859.1, XP_062526863.1, XP_062526861.1	ETV5-related protein Ets96B isoform X2 ETV5-related protein Ets96B isoform X1 ETV5-related protein Ets96B isoform X1 ETV5-related protein Ets96B isoform X1 ETV5-related protein Ets96B isoform X1 ETV5-related protein Ets96B isoform X1 ETV5-related protein Ets96B isoform X1
LOC134201466	449.727037479727	-2.78559409415699	0.00170350254883932		uncharacterized LOC134201466
LOC101737288	1558.45454814002	-1.93346479574474	0.00171139615975949	XP_004923867.1	uncharacterized protein LOC101737288
LOC101744900	541.721087191047	-2.52938536632064	0.00171139615975949	XP_062529206.1, XP_037872689.1	dynein axonemal heavy chain 3 isoform X2 dynein axonemal heavy chain 3 isoform X1
LOC101746375	1018.48634250976	-3.93333888203995	0.00171626912231461	XP_012543882.3, XP_037867057.1	cell adhesion molecule Dscam2 isoform X1 cell adhesion molecule Dscam2 isoform X2
LOC110386610	25.0822996602347	-4.76969530087125	0.00171626912231461		uncharacterized protein LOC110386610
LOC101737238	6190.53828003343	-3.07987300249038	0.00171626912231461	XP_062530292.1, XP_004922563.1	microvitellogenin isoform X1 microvitellogenin isoform X2
LOC101741238	5642.06225375047	-2.67999757988904	0.00171626912231461	XP_004923430.1	eIF-2-alpha kinase GCN2

LOC101747101	264721.085231134	-2.92349422448752	0.00171904811864477	XP_012546247.1,<br>XP_062529234.1	acylamino-acid-releasing enzyme
LOC101739397	946.065210916597	-3.21919505998869	0.00171907780867508	XP_062524384.1,<br>XP_004925831.1,<br>XP_004925832.1	uncharacterized protein LOC101739397 isoform X3,<br>uncharacterized protein LOC101739397 isoform X1,<br>uncharacterized protein LOC101739397 isoform X2
ae19	765.647846956449	-1.97217349041666	0.00171907780867508	NP_001116501.1,<br>NP_001166806.1	alpha-esterase 19 isoform 1 precursor<br>alpha-esterase 19 isoform 2 precursor
NGR-B1	2128.70938623791	-2.32314877395802	0.00171932732755106	NP_001127732.1,<br>XP_037868159.1,<br>XP_037868153.1,<br>XP_062525503.1,<br>XP_062525501.1,<br>XP_062525504.1,<br>XP_037868156.1,<br>XP_062525505.1,<br>XP_037868158.1,<br>XP_037868157.1,<br>XP_062525506.1	Diuretic hormone 31 Receptor<br>neuropeptide receptor B1 isoform X1<br>neuropeptide receptor B1 isoform X1<br>neuropeptide receptor B1 isoform X2<br>neuropeptide receptor B1 isoform X1<br>neuropeptide receptor B1 isoform X2<br>neuropeptide receptor B1 isoform X1<br>neuropeptide receptor B1 isoform X1<br>neuropeptide receptor B1 isoform X3<br>neuropeptide receptor B1 isoform X1<br>neuropeptide receptor B1 isoform X3
LOC101746812	1147.01455888819	-2.95671803187227	0.00172477173406452	XP_012549568.1,<br>XP_062525074.1	lachesin isoform X1<br>lachesin isoform X2
LOC101744025	480.390653496882	-4.20361921623457	0.00181121294427755	XP_062529284.1	chorion class B protein M2410-like
LOC119628655	601.116855094028	-2.88555762733202	0.00181121294427755		uncharacterized LOC119628655
LOC110384995	21.4503234365751	-4.61998429606582	0.00181121294427755		Uncharacterized LOC110384995
LOC105842097	301.686884900091	-1.87807277261137	0.00181412860927432	XP_062533110.1,<br>XP_012548509.1	uncharacterized protein LOC105842097 isoform X2,<br>uncharacterized protein LOC105842097 isoform X1
LOC101743222	6997.83616181195	-2.30710936958498	0.00183068845272942	XP_037870952.1,<br>XP_037870954.1,<br>XP_037870954.1	fat-like cadherin-related tumor suppressor homolog isoform X1, fat-like cadherin-related tumor suppressor homolog isoform X2, fat-like cadherin-related tumor suppressor homolog isoform X1
LOC105842367	226.668783272784	-1.95522403773861	0.00183523026383939	XP_012550362.2	uncharacterized protein LOC105842367
LOC101737574	1196.09056746143	-1.330053841223	0.00184306474427292	XP_004924203.1,<br>XP_021202374.1	dihydroorotate dehydrogenase (quinone) mitochondrial isoform X2<br>dihydroorotate dehydrogenase (quinone) isoform X1

LOC101745350	558.01176968656	-3.53157269819113	0.00192515605346224	XP_062524623.1, XP_004932324.1	uncharacterized protein LOC101745350
LOC110386034	86.1641427830908	-3.34768922746656	0.00193272703870107		uncharacterized LOC110386034
LOC101743809	223.223265821599	1.33356236636467	0.00193537455169746	XP_037877132.1	vanin-like protein 1
LOC101742301	969.723485509406	-2.0843285244402	0.00193968080807449	XP_012546709.1, XP_012546710.1, XP_021204355.1	hemicentin-1 isoform X1 hemicentin-1 isoform X2 hemicentin-1 isoform X1
LOC119628747	19.3691281187147	3.84807993793631	0.0019510812260865	XP_062525263.1	ATP-dependent DNA helicase pif1-like
LOC101736718	605.427787151267	-2.33333756068357	0.00195276504314984	XP_012547222.2, XP_021204877.2, XP_021204878.2	ankyrin repeat and BTB/POZ domain-containing protein 3 isoform X3, ankyrin repeat and BTB/POZ domain-containing protein 3 isoform X1, ankyrin repeat and BTB/POZ domain-containing protein 3 isoform X2
LOC101744990	25082.7583934652	-2.04363790317407	0.00195489434358441	XP_037870767.1, XP_062527829.1	DENN domain-containing protein 5B isoform X1 DENN domain-containing protein 5B isoform X2
LOC101745343	8175.41445295302	-2.18385331633714	0.00195489434358441	XP_037872474.1	PAX-interacting protein 1
LOC101742046	1115.18279437166	-2.17775435750417	0.00196696999726681	XP_037873961.1	zinc finger protein 808
LOC101744340	36.4742405892625	-2.3092783406052	0.00196880565642584	XP_004932270.2	uncharacterized protein LOC101744340
LOC101743174	9333.51738625978	-2.4296921508327	0.00196949370208388	XP_012546137.1	hemicentin-1
LOC101739353	23434.3528027589	-1.14586843986504	0.00203148795129586	XP_037869964.1, XP_037869963.1	low-density lipoprotein receptor-related protein 2 isoform X2, low-density lipoprotein receptor-related protein 2 isoform X1
LOC134198692	13312.8182454652	-3.4749621309392	0.00203204475263464	XP_062525211.1	choline O-acetyltransferase
LOC101735433	723.356574155404	-2.69425063910013	0.00203204475263464	XP_037867423.1, XP_037867424.1, XP_037867421.1, XP_037867420.1, XP_062525084.1	uncharacterized protein LOC101735433 isoform X3, uncharacterized protein LOC101735433 isoform X5, uncharacterized protein LOC101735433 isoform X2, uncharacterized protein LOC101735433 isoform X1, uncharacterized protein LOC101735433 isoform X4
LOC134199109	36.5045888755248	-2.56851751075503	0.00203204475263464	XP_062525265.1	nascent polypeptide-associated complex subunit alpha, muscle-specific form-like
Gata-beta	11781.3923952301	-2.23418105882946	0.00206033872319007	NP_001037446.1, XP_037871549.1,	transcription factor BCFI, transcription factor BCFI isoform X5,

				XP_062528329.1,<br>XP_037871550.1,<br>XP_037871546.1,<br>XP_037871547.1,<br>XP_037871548.1	transcription factor BCFI isoform X4,<br>transcription factor BCFI isoform X6,<br>transcription factor BCFI isoform X1,<br>transcription factor BCFI isoform X2,<br>transcription factor BCFI isoform X3
LOC110386413	62.0964725746436	-1.37715108383664	0.00206089487616821		Uncharacterized LOC110386413
LOC101742761	22429.1601786535	-2.01426219990836	0.00206089487616821	XP_012545379.1	ankyrin repeat domain-containing protein 12
LOC101745757	35.211879427618	-2.40325169415463	0.00207066677306107	XP_062526061.1	uncharacterized protein LOC101745757
LOC134199575	21.7749644077775	-2.83368911854402	0.00207716514184204		Uncharacterized LOC134199575
LOC101740680	211.918790767458	-1.6929353787899	0.0021052122063618	XP_037875317.1	Uncharacterized LOC101740680
MLC-2	32768.9920411815	-1.53407489855326	0.0021052122063618	NP_001091813.1	myosin regulatory light chain 2
LOC101738454	1712.12432720299	-1.76039366373303	0.0021388505653715	XP_021206661.1,<br>XP_012549827.1,<br>XP_021206663.1,<br>XP_062531311.1	tau-tubulin kinase homolog Asator isoform X1<br>tau-tubulin kinase homolog Asator isoform X3<br>tau-tubulin kinase homolog Asator isoform X2<br>tau-tubulin kinase homolog Asator isoform X1
LOC105842364	82.6938406556721	-3.27286264514446	0.00228444648120371	XP_012550339.1	uncharacterized protein LOC105842364
LOC101742254	84865.5244793361	-2.91672489650717	0.00228444648120371	XP_021203848.2,<br>XP_037874951.1,<br>XP_037874955.1,<br>XP_062530957.1,<br>XP_062530959.1,<br>XP_037874953.1,<br>XP_062530956.1,<br><br>XP_037874952.1,<br>XP_062530955.1,<br><br>XP_021203851.1,<br>XP_062530952.1,<br>XP_062530951.1,<br>XP_062530951.1,<br>XP_062530958.1,	cAMP-specific 3',5'-cyclic phosphodiesterase isoform X3,<br>cAMP-specific 3',5'-cyclic phosphodiesterase isoform X4,<br>cAMP-specific 3',5'-cyclic phosphodiesterase isoform X11,<br>cAMP-specific 3',5'-cyclic phosphodiesterase isoform X11,<br>cAMP-specific 3',5'-cyclic phosphodiesterase isoform X13,<br>cAMP-specific 3',5'-cyclic phosphodiesterase isoform X8,<br>cAMP-specific 3',5'-cyclic phosphodiesterase isoforms N/G isoform X10,<br>cAMP-specific 3',5'-cyclic phosphodiesterase isoform X7,<br>cAMP-specific 3',5'-cyclic phosphodiesterase, isoforms N/G isoform X9,<br>cAMP-specific 3',5'-cyclic phosphodiesterase isoform X7,<br>cAMP-specific 3',5'-cyclic phosphodiesterase isoform X1,<br>cAMP-specific 3',5'-cyclic phosphodiesterase isoform X1,<br>cAMP-specific 3',5'-cyclic phosphodiesterase isoform X2,<br>cAMP-specific 3',5'-cyclic phosphodiesterase isoform X12,<br>cAMP-specific 3',5'-cyclic phosphodiesterase isoform X12,

				XP_021203852.1, XP_021203849.1, XP_062530954.1, XP_021203853.1, XP_037874957.1	cAMP-specific 3',5'-cyclic phosphodiesterase isoforms N/G isoform X5, cAMP-specific 3',5'-cyclic phosphodiesterase isoforms N/G isoform X6, cAMP-specific 3',5'-cyclic phosphodiesterase isoform X14, cAMP-specific 3',5'-cyclic phosphodiesterase isoform X15
LOC101745832	452.53839350074	-1.69398443680128	0.00235008704063943	XP_004929824.1	transcription initiation factor TFIID subunit 7
LOC134200161	22.5250609895011	-2.95439542601274	0.00235008704063943		uncharacterized LOC134200161
LOC101744840	111.274275918965	-2.07534808194956	0.00235008704063943	XP_021203784.1, XP_004926341.1, XP_021203785.1	uncharacterized protein LOC101744840
LOC134199469	48.166344413758	-1.46416782183228	0.00239070710693217		uncharacterized LOC134199469
LOC733030	81636.4654590786	-1.16054504262553	0.00239070710693217	NP_001037594.1, XP_062526334.1, XP_012552964.1,	troponin C troponin C isoform X2 troponin C isoform X1
LOC101744910	1631.76321926275	-3.83904527846232	0.00239070710693217	XP_062529385.1, XP_062529386.1	voltage-dependent T-type calcium channel subunit alpha-1G isoform X1, voltage-dependent T-type calcium channel subunit alpha-1G isoform x2
LOC101739731	702.771470651993	-3.05254542408158	0.00239070710693217	XP_021203274.1	odorant receptor 4
LOC101742731	1298.01205306944	-3.11080139718415	0.00239223770556483	XP_012545353.1	uncharacterized protein LOC101742731
LOC101737821	3570.12505454983	-1.86365687013836	0.00241904527684582	XP_037876048.1, XP_037876047.1, XP_037876049.1	RNA-binding protein 25 isoform X1, RNA-binding protein 25 isoform X1, RNA-binding protein 25 isoform X2
LOC101745031	1173.40831965474	-2.95600545028304	0.00253981418178439	XP_012544933.1	lachesin
LOC101739367	210.055817444995	-1.46718462279267	0.00255672567245622	XP_062526243.1, XP_062526240.1, XP_062526242.1, XP_062526237.1, XP_062526241.1, XP_062526239.1	serine/threonine-protein phosphatase 6 regulatory ankyrin repeat subunit C
LOC101745484	233.091816342266	-2.84177185036129	0.00256470980163575	XP_012548092.2	serine protease gd

LOC119628412	236717.746874873	-3.14632353307513	0.00258226267873346	XP_037875321.1	exocyst complex component Sec3
LOC101745616	236721.680989609	-3.14632879435844	0.00258226267873346	XP_037875324.2	exocyst complex component 1
LOC101738245	13167.3191543604	-2.93111411562097	0.00262085492526664	XP_004923733.3,<br>XP_012543793.2,<br>XP_012543792.2	zinc finger protein 35 isoform X3<br>zinc finger protein 35 isoform X2<br>zinc finger protein 35 isoform X1
LOC110384806	5881.12411275705	-2.74673768091513	0.00266836916927406	XP_037871191.1	transmembrane protein 131 homolog
LOC101740990	42.4527374008405	-2.38746241577588	0.00271320210578395	XP_062526691.1,<br>XP_004921693.2	echinoderm microtubule-associated protein-like CG42247 isoform X2<br>echinoderm microtubule-associated protein-like CG42247 isoform X1
LOC101740152	904.060691140069	-2.63530620792868	0.00272620236039976	XP_062530683.1,<br>XP_062530682.1,<br>XP_012548773.3	putative odorant receptor 19b isoform X2,<br>odorant receptor 43a isoform X1,<br>odorant receptor 43a isoform X1
LOC101736412	76.401000271016	-2.59683358887912	0.00274883243002153	XP_004928768.1	uncharacterized protein LOC101736412
LOC119629021	904.632345083739	-2.62936775113245	0.00276570383497118		uncharacterized LOC119629021
LOC134201666	739.777559619497	-3.53709040838522	0.00276570383497118	XP_062532896.1,<br>XP_062532897.1	uncharacterized protein LOC134201666
LOC101743841	49.4082834914405	-2.21865548110166	0.00277980249492892	XP_004929096.2	uncharacterized protein LOC101743841
LOC101747162	547.833119973123	-1.93748408197839	0.002878043137222	XP_004924187.1,<br>XP_062527036.1,<br>XP_062527037.1	serine/threonine-protein kinase ULK3 isoform X2,<br>serine/threonine-protein kinase ULK3 isoform X1,<br>serine/threonine-protein kinase ULK3 isoform X3
LOC101742538	36477.1093743713	-4.20171980602148	0.0029060092744899	XP_004925774.1	cyclin-J
LOC101742594	538868.124022344	-2.55920540485232	0.0029258073179996	XP_004926820.1,<br>XP_021204074.1,<br>XP_012546369.1,<br>XP_062532441.1,<br>XP_037876755.1	dihydropyrimidinase isoform X2,<br>dihydropyrimidinase isoform X3,<br>dihydropyrimidinase isoform X1,<br>dihydropyrimidinase isoform X4,<br>dihydropyrimidinase isoform X5
LOC134200213	5508.52103232959	-3.95187762512196	0.00293301683809607		uncharacterized LOC134200213
LOC105841880	5323.05146939105	-1.63249907854404	0.00307146157455925	XP_062526741.1,<br>XP_037869252.1,<br>XP_062526739.1,	isintegrin and metalloproteinase domain-containing protein 11 isoform X5,<br>isintegrin and metalloproteinase domain-containing protein 11 isoform X10,

				XP_037869247.1, XP_062526742.1, XP_062526743.1, XP_037869246.1, XP_062526740.1, XP_062526745.1, XP_062526744.1	isintegrin and metalloproteinase domain-containing protein 11 isoform X2, isintegrin and metalloproteinase domain-containing protein 11 isoform X4, isintegrin and metalloproteinase domain-containing protein 11 isoform X6, isintegrin and metalloproteinase domain-containing protein 11 isoform X7, isintegrin and metalloproteinase domain-containing protein 11 isoform X1, isintegrin and metalloproteinase domain-containing protein 11 isoform X3, isintegrin and metalloproteinase domain-containing protein 11 isoform X9, isintegrin and metalloproteinase domain-containing protein 11 isoform X8
LOC101741193	991.819136694867	-2.11184046198258	0.0030902723922614	XP_004922248.1	ATP-dependent RNA helicase abstrakt
LOC101745121	1137.24392233227	-2.62211852697881	0.0030902723922614	XP_037871877.1	PAX3- and PAX7-binding protein 1
LOC101742550	2664.11075481144	-1.79376870296142	0.0030902723922614	XP_062528923.1, XP_062528922.1, XP_037872138.2	eukaryotic translation initiation factor 4E transporter isoform X3, eukaryotic translation initiation factor 4E transporter isoform X1, eukaryotic translation initiation factor 4E transporter isoform X2
LOC119629025	138.899331652508	-4.97772960204017	0.00313057824747691		uncharacterized LOC119629025
LOC101742874	277.932323765707	-1.61209663703829	0.00318292537033618	XP_037868452.1, XP_037868450.1	kinesin-like protein KIF12 isoform X2, kinesin-like protein KIF12 isoform X1
LOC134199003	121.190645725971	-2.96137697224328	0.00321783376593046		uncharacterized LOC134199003
LOC101741863	792.783168102059	-1.22958297329762	0.00327444921866296	XP_037877798.1, XP_037877799.1	uncharacterized protein LOC101741863
LOC101743475	11252.9744991921	-2.19492723051584	0.00340926892509656	XP_004932396.1, XP_012551171.1, XP_004932397.1	syndecan isoform X1, syndecan isoform X2, syndecan isoform X3
LOC101740797	60.0931857348631	-3.36811645176256	0.00340926892509656		uncharacterized LOC101740797

LOC101737164	138.083412004907	-2.73234797648271	0.00343812638967169	XP_012549281.1, XP_021206286.1	uncharacterized protein LOC101737164 isoform X1, uncharacterized protein LOC101737164 isoform X2
LOC134198853	431.911905771069	-2.80190236688578	0.00344287296148639	XP_062524467.1	uncharacterized protein LOC134198853
LOC110385059	89010.804089666	-3.32601550489173	0.0034454448439948	XP_037868471.1	E3 ubiquitin-protein ligase Ubr3
LOC101745689	515.982243249971	-2.73788321282228	0.00344980407499508	XP_037869690.1	intraflagellar transport protein 172 homolog
LOC101745976	2532.42178326498	-1.5492731299081	0.00358556127656548	XP_037867875.1, XP_037867873.1, XP_037867874.1, XP_037867876.1, XP_037867878.1, XP_037867883.1, XP_062525403.1, XP_037867882.1, XP_037867881.1, XP_037867879.1, XP_037867880.1, XP_037867884.1, XP_037867886.1	trithorax group protein osa isoform X2, trithorax group protein osa isoform X1, trithorax group protein osa isoform X3, trithorax group protein osa isoform X4, trithorax group protein osa isoform X5, trithorax group protein osa isoform X11, trithorax group protein osa isoform X7, trithorax group protein osa isoform X10, trithorax group protein osa isoform X9, trithorax group protein osa isoform X6, trithorax group protein osa isoform X8, trithorax group protein osa isoform X12, trithorax group protein osa isoform X13
LOC101739994	498.801702889561	-2.89360503133768	0.00360921582021482	XP_062525209.1	probable sodium/potassium-transporting ATPase subunit beta-3
LOC101736446	894.406792920708	-1.92164724884078	0.00363985171177676	XP_012547608.3, XP_062529556.1, XP_062529554.1, XP_062529555.1	disheveled-associated activator of morphogenesis 1 isoform X1, disheveled-associated activator of morphogenesis 1 isoform X3, disheveled-associated activator of morphogenesis 1 isoform X1, disheveled-associated activator of morphogenesis 1 isoform X2
LOC134200401	23.0596573831131	-3.07848066176849	0.00370052831616018		uncharacterized LOC134200401
LOC134199335	63.5791509472538	-2.06628326188974	0.00370592248381666		uncharacterized LOC134199335
LOC101736013	48.5779668744761	-2.27589981005447	0.00370592248381666	XP_062528856.1, XP_012548289.2, XP_062528857.1	O-acyltransferase like protein isoform X2, O-acyltransferase like protein isoform X1, O-acyltransferase like protein isoform X3
LOC105842234	4720.82249169382	-2.98719906469061	0.00370825438094013	XP_012549506.1	uncharacterized protein LOC105842234
LOC134201159	453.65390927695	-2.77779450576316	0.0037101195794384	XP_062531459.1	uncharacterized protein LOC134201159
LOC101740156	297.697130469066	-3.32884182973394	0.00387073653379134	XP_062531075.1	synapsin

Myb	15866.8927153774	-2.86394402523212	0.0038812404092482	NP_001244265.1, XP_037876990.1, XP_062532676.1, XP_037876988.1, XP_037876989.1	proto-oncogene like protein Myb proto-oncogene like protein Myb myb transcription factor isoform X3, proto-oncogene like protein Myb myb transcription factor isoform X4, proto-oncogene like protein Myb myb transcription factor isoform X1, proto-oncogene like protein Myb myb transcription factor isoform X2
Abd-b	3143.93356075736	-2.76305528722097	0.00388262013048145	NP_001139700.1, NP_001166802.1, XP_062524856.1, XP_012549559.1	abdominal B isoform RBL, abdominal B isoform RBS, abdominal B isoform X1, abdominal B isoform X1
LOC101745387	6392.26625747108	-2.90344807352791	0.0038837808174638	XP_037871743.1	protein crumbs
LOC119630688	140200.780023563	-1.69296165948043	0.00393957968415394	XP_037876902.1	uncharacterized protein LOC119630688
LOC101745111	5178.43430102613	-1.87565449558563	0.00400492910416972	XP_012551514.3, XP_012551512.1	protein jim lovell isoform X2, uncharacterized protein LOC101745111 isoform X1
LOC100101168	15313.4355760845	-2.15062747403545	0.00400492910416972	NP_001093266.1, NP_001093266.1	OCIA domain-containing protein asrij isoform 1, OCIA domain-containing protein asrij isoform 2
LOC101740354	7141.60332122634	-1.70513730284301	0.00400492910416972	XP_037872423.1, XP_062529095.1, XP_062529096.1, XP_062529093.1, XP_062529094.1, XP_062529092.1	myotubularin-related protein 3 isoform X1, myotubularin-related protein 3 isoform X2, myotubularin-related protein 3 isoform X3, myotubularin-related protein 3 isoform X1, myotubularin-related protein 3 isoform X1, myotubularin-related protein 3 isoform X1
LOC101738046	1615.56210479459	-2.21542083734601	0.00406975390445013	XP_037870706.1	cytoplasmic dynein 2 heavy chain 1
LOC134200553	17.9420233528991	-3.84602579773717	0.00417621954609285		uncharacterized LOC134200553
LOC101743257	39442.5170415699	-2.08360429200053	0.00421328154621169	XP_037874420.1, XP_012545377.1, XP_062530616.1	uncharacterized protein LOC101743257
LOC119630273	25.5845213644729	-3.15132278240065	0.00421328154621169		uncharacterized LOC119630273
LOC101737230	147.380626979931	-2.50056504420901	0.00421895550332337	XP_062529907.1	glutamate receptor ionotropic, kainate 2
LOC134199210	422.045766879258	-2.78172374189163	0.004376529393081	XP_062525560.1	uncharacterized protein LOC134199210

Amy	143.972258443272	3.33009899179077	0.0044685781775768	NP_001166624.1	alpha-amylase precursor
LOC134199185	2753.13277078208	-4.02974186382452	0.00449458937886591	XP_062525458.1, XP_062525456.1, XP_062525460.1, XP_062525459.1, XP_062525457.1	uncharacterized protein LOC134199185
LOC101737715	756.161079758794	-2.50624475536052	0.0046327344882936	XP_037874609.1	zinc finger protein 791
LOC101739610	209.516909423675	-2.53646823943016	0.0047174182718274	XP_037868805.1, XP_037868804.1, XP_037868807.1, XP_037868806.1, XP_062526248.1, XP_062526247.1, XP_062526249.1	uncharacterized protein LOC101739610 isoform X2, uncharacterized protein LOC101739610 isoform X1, uncharacterized protein LOC101739610 isoform X4, uncharacterized protein LOC101739610 isoform X3, uncharacterized protein LOC101739610 isoform X2, uncharacterized protein LOC101739610 isoform X1, uncharacterized protein LOC101739610 isoform X4
LOC101746187	1855.76672137832	-2.02246376759288	0.00476480632753112	XP_062531143.1, XP_021203209.1, XP_062531145.1, XP_062531144.1, XP_062531142.1, XP_021203210.1, XP_021203213.1	KAT8 regulatory NSL complex subunit 1 isoform X1, KAT8 regulatory NSL complex subunit 1 isoform X1, KAT8 regulatory NSL complex subunit 1-like protein isoform X3, KAT8 regulatory NSL complex subunit 1 isoform X2, KAT8 regulatory NSL complex subunit 1 isoform X1, KAT8 regulatory NSL complex subunit 1 isoform X1, KAT8 regulatory NSL complex subunit 1-like protein isoform X4
rptor	4542.83973894119	-2.91597532639377	0.00486370463451958	NP_001177772.1, XP_037872553.1, XP_037872554.1	regulatory associated protein of MTOR complex 1, regulatory-associated protein of TOR isoform X1, regulatory-associated protein of TOR isoform X2
LOC101743074	1230.38649578905	-2.80795582039229	0.00486370463451958	XP_062532345.1, XP_062532343.1, XP_037876538.1, XP_062532344.1, XP_012545774.2, XP_037876539.1	alpha-sarcoglycan isoform X4, alpha-sarcoglycan isoform X2, alpha-sarcoglycan isoform X1, alpha-sarcoglycan isoform X3, alpha-sarcoglycan isoform X1, alpha-sarcoglycan isoform X1, alpha-sarcoglycan isoform X1, alpha-sarcoglycan isoform X3,

					alpha-sarcoglycan isoform X1, alpha-sarcoglycan isoform X1,<br>alpha-sarcoglycan isoform X1, alpha-sarcoglycan isoform X1,
LOC101743597	743658.904807646	-2.2925718313708	0.00494583618362473	XP_062527262.1,<br>XP_037870078.1,<br>XP_037870079.1	unconventional myosin-IXAa isoform X1,<br>unconventional myosin-IXAa isoform X2,<br>unconventional myosin-IXAa isoform X3
LOC101739117	49.9890890937354	-2.41410365450563	0.0050394645458477	XP_004924782.2	uncharacterized protein LOC101739117
LOC110386384	80.7648985466966	-2.36303046951233	0.00506417172990874		uncharacterized LOC110386384
LOC101742803	1391.93709687053	-2.80793030829144	0.00506907681540487	XP_004930786.2	HMG box-containing protein 4
LOC101742164	656.389193527355	-2.06984898713679	0.00535997570763953	XP_004928471.2	uncharacterized protein LOC101742164
LOC101738561	3697.25506233704	-2.227516735751	0.00541200620396937	XP_037872272.1,<br>XP_037872273.1	HIRA-interacting protein 3 isoform X1,<br>HIRA-interacting protein 3 isoform X2
LOC101735943	224.96532682019	-2.53528233512078	0.00543162295009168	XP_012552169.1	palmitoyltransferase ZDHHC11
LOC105841801	42.6567190545778	-2.83543400427607	0.00543162295009168		uncharacterized LOC105841801
LOC101738428	764.3828167232	-3.339239093975	0.00548050578885444	XP_004922421.1,<br>XP_037873756.1	uncharacterized protein LOC101738428
LOC101746902	660.007152145714	-2.37401290749781	0.00554567124972961	XP_012546425.1,<br>XP_021204124.1,<br>XP_004926912.2	zinc finger protein 84 isoform X1,<br>zinc finger protein 84 isoform X3,<br>zinc finger protein 84 isoform X2
LOC101739771	668.074551871061	-2.13412136635306	0.00554746277170881	XP_037871322.1	apoptosis-inducing factor 1, mitochondrial
LOC101738309	281.504911737812	-3.05902092813495	0.00578438186276998	XP_062529717.1	uncharacterized protein LOC101738309
LOC110385041	1468.43866864892	-2.0418784479999	0.0058999039951562	XP_037867453.1,<br>XP_037867455.1	uncharacterized protein LOC110385041 isoform X1,<br>uncharacterized protein LOC110385041 isoform X2
LOC101736792	139.469612430839	-1.9873583844159	0.00590310185333588	XP_004923863.1	alpha-crystallin A chain
LOC134201433	40.8950015877391	5.85640694714566	0.00592548581116057		uncharacterized LOC134201433
LOC101747117	1505.92524886139	-2.58600638818576	0.00600857603788895	XP_037873556.1,<br>XP_037873555.1	uncharacterized protein LOC101747117 isoform X2,<br>uncharacterized protein LOC101747117 isoform X1
LOC101736178	149.586395260282	-3.12206946377504	0.00631550453976351	XP_062530580.1	zwei Ig domain protein zig-8
LOC101741396	11644.8138572984	-2.74186662087401	0.0064973766131561	XP_037872223.1	phosphatidylinositol 4-kinase alpha
Eck	22.77922351981	-2.24468514998655	0.0064973766131561	NP_001038956.1	ecdysteroid 22-kinase

LOC101738329	842.266622752609	-1.18620054499238	0.00660620960901209	XP_037871879.1, XP_004923057.1, XP_021209061.1	E3 ubiquitin-protein ligase RNF13
LOC101743873	5583.81102094407	-4.08931009061168	0.00665185469217461	XP_012553435.2	protein HID1
LOC119629806	20.2846403617746	2.90057052856744	0.00667981255636345		uncharacterized LOC119629806
LOC101735787	286.530688366127	-2.00599537999644	0.00667981255636345	XP_004925943.1, XP_062529533.1	zinc finger protein 77 isoform X1, zinc finger protein 77 isoform X2
LOC105842612	38.3439254367563	4.79486588509655	0.0067182922127694	XP_021208078.1, XP_021208082.1	uncharacterized protein LOC105842612 isoform X1, uncharacterized protein LOC105842612 isoform X2
LOC101745667	4170.94764254293	-2.30099569342567	0.0067182922127694	XP_037876561.1	serine/threonine-protein kinase pelle
LOC101739972	6000.69732298218	-2.02059871465033	0.00676643864658254	XP_062528415.1, XP_062528416.1, XP_062528417.1, XP_037872034.1, XP_062528418.1	protein purity of essence isoform X1, protein purity of essence isoform X2, protein purity of essence isoform X4, protein purity of essence isoform X3, protein purity of essence isoform X5
LOC101746044	30.0506658204694	-1.72672024455106	0.00715012931199716	XP_037875093.2	ionotropic receptor 93a
LOC101737197	3781.23074487655	-1.95030652962252	0.00723699570179399	XP_037873564.1, XP_062529828.1	cleavage and polyadenylation specificity factor 73 isoform X1, cleavage and polyadenylation specificity factor 73 isoform X2
LOC101742943	332.597398498176	-2.34484711085773	0.0072668750944018	XP_037877788.1, XP_062533108.1, XP_037877786.1, XP_062533106.1, XP_062533104.1, XP_037877790.1, XP_037877789.1, XP_037877787.1, XP_037877785.1, XP_062533107.1, XP_062533105.1	neuroligin-4 X-linked isoform X1, neuroligin-4 X-linked isoform X1, neuroligin-4 X-linked isoform X1, neuroligin-4 X-linked isoform X1, neuroligin-4 X-linked isoform X1, neuroligin-1 isoform X3, neuroligin-4 X-linked isoform X2 neuroligin-4 X-linked isoform X1, neuroligin-4 X-linked isoform X1, neuroligin-4 X-linked isoform X1, neuroligin-4 X-linked isoform X1
LOC134199907	27.4859620858565	-2.76637380740634	0.00729665813092964		uncharacterized LOC134199907
LOC119630132	50.9994776910005	-2.38645263402345	0.00752383283977786	XP_037874551.1	uncharacterized protein LOC119630132
LOC101745704	80.0279919486681	-1.98237293583292	0.0077512432797165	XP_037872075.1	diacylglycerol kinase eta

LOC101745162	1399.91586246581	-2.66943077906274	0.0077512432797165	XP_037873475.1,<br>XP_037873478.1,<br>XP_062529945.1,<br>XP_037873476.1,<br>XP_037873479.1,<br>XP_062529944.1,<br>XP_062529943.1,<br>XP_062529941.1,<br>XP_062529942.1,<br>XP_062529953.1,<br>XP_062529955.1,<br>XP_062529951.1,<br>XP_062529952.1,<br>XP_062529954.1,<br>XP_062529948.1,<br>XP_062529946.1,<br>XP_062529938.1,	potassium/sodium hyperpolarization-activated cyclic nucleotide-gated channel 2 isoform X1,<br>potassium/sodium hyperpolarization-activated cyclic nucleotide-gated channel 2 isoform X3,<br>potassium/sodium hyperpolarization-activated cyclic nucleotide-gated channel 2 isoform X10,<br>potassium/sodium hyperpolarization-activated cyclic nucleotide-gated channel 2 isoform X2,<br>potassium/sodium hyperpolarization-activated cyclic nucleotide-gated channel 2 isoform X4,<br>potassium/sodium hyperpolarization-activated cyclic nucleotide-gated channel 2 isoform X9,<br>potassium/sodium hyperpolarization-activated cyclic nucleotide-gated channel 2 isoform X9,<br>potassium/sodium hyperpolarization-activated cyclic nucleotide-gated channel 2 isoform X8,<br>potassium/sodium hyperpolarization-activated cyclic nucleotide-gated channel 2 isoform X9,<br>potassium/sodium hyperpolarization-activated cyclic nucleotide-gated channel 2 isoform X16,<br>potassium/sodium hyperpolarization-activated cyclic nucleotide-gated channel 2 isoform X17,<br>potassium/sodium hyperpolarization-activated cyclic nucleotide-gated channel 2 isoform X16,<br>potassium/sodium hyperpolarization-activated cyclic nucleotide-gated channel 2 isoform X17,<br>potassium/sodium hyperpolarization-activated cyclic nucleotide-gated channel 2 isoform X16,<br>potassium/sodium hyperpolarization-activated cyclic nucleotide-gated channel 2 isoform X13,<br>potassium/sodium hyperpolarization-activated cyclic nucleotide-gated channel 2 isoform X11,<br>potassium/sodium hyperpolarization-activated cyclic nucleotide-gated channel 2 isoform X5,

				XP_062529939.1, XP_062529947.1, XP_062529940.1, XP_062529949.1, XP_062529950.1	potassium/sodium hyperpolarization-activated cyclic nucleotide-gated channel 2 isoform X6, potassium/sodium hyperpolarization-activated cyclic nucleotide-gated channel 2 isoform X12, potassium/sodium hyperpolarization-activated cyclic nucleotide-gated channel 2 isoform X7, potassium/sodium hyperpolarization-activated cyclic nucleotide-gated channel 2 isoform X14, potassium/sodium hyperpolarization-activated cyclic nucleotide-gated channel 2 isoform X15
LOC101735441	1814.40489673242	-1.67151552103778	0.00777126477426404	XP_021208347.2	uncharacterized protein LOC101735441
LOC101746252	16.4181347507181	-3.86296132375816	0.00777126477426404	XP_004928121.1	MORN repeat-containing protein 4 homolog
LOC101742029	810.407609480876	-1.73717548670637	0.00791934831448249	XP_037866644.1, XP_037866646.1, XP_037866645.1	E3 ubiquitin-protein ligase lubel isoform X1, E3 ubiquitin-protein ligase lubel isoform X3, E3 ubiquitin-protein ligase lubel isoform X2
LOC101744439	50.4167422828404	-2.30172419820727	0.00791934831448249	XP_037867065.1	neurogenic protein big brain
LOC101736902	1849.55314067278	-1.4840628434656	0.00791934831448249	XP_021206905.2, XP_012550225.2, XP_037868690.1	tolloid-like protein 1
Ser-4	365.05013341141	-2.03350750872338	0.00791934831448249	NP_001037502.1, XP_062526327.1	5-hydroxytryptamine receptor, 5-hydroxytryptamine receptor isoform X1
LOC101742088	25.8899701995222	-2.10440892961952	0.00791934831448249	XP_012552972.1	protein PTHB1
LOC101742142	250.201475248905	-2.32287287564197	0.00791934831448249	XP_037871334.1	nascent polypeptide-associated complex subunit alpha, muscle-specific for
LOC110384702	93.2051771258374	-1.557120514761	0.0080807939040636	XP_021208475.1	uncharacterized protein LOC110384702
Ser3	712.588735526802	-3.6271651200699	0.00812713609300393	NP_001108116.1	sericin 3
LOC101744255	15.2367749711927	4.20129267607069	0.00815568232110517	XP_037867765.1	ATP-dependent DNA helicase pif1-like
LOC101737629	3276.12087245347	-1.76514707941786	0.00817376293791288	XP_037877609.1, XP_062533048.1, XP_037877610.1	protein transport protein Sec31A
LOC101737387	14302.7067562528	-2.25713392903071	0.00825345798590169	XP_004923657.1, XP_062525865.1	AN1-type zinc finger protein 1 isoform X1, AN1-type zinc finger protein 1 isoform X2
LOC119629893	79.5808061723338	-3.15126989588646	0.00834030608879793	XP_037873346.1	uncharacterized protein LOC119629893

LOC101735329	1598.16398844001	-2.05734191765683	0.008342261525425	XP_062524203.1, XP_004927520.1, XP_021204553.1, XP_004927519.1	katanin p80 WD40 repeat-containing subunit B1 isoform X4, katanin p80 WD40 repeat-containing subunit B1 isoform X2, katanin p80 WD40 repeat-containing subunit B1 isoform X3, katanin p80 WD40 repeat-containing subunit B1 isoform X1
LOC134200008	22920.7742800134	-2.67116713819557	0.00844265988379488		uncharacterized LOC134200008
LOC101736485	271.551869954412	-1.8336290268861	0.00844265988379488	XP_004928597.4	zyxin
LOC119631009	53.4154792652377	-2.90995620907474	0.00851677557150828	XP_004923495.1	zinc finger protein GLI2-like
LOC101737509	617.539877482303	-1.55980443513022	0.00881717651120267	XP_062531295.1, XP_037875100.1, XP_037875097.1, XP_037875099.1, XP_062531296.1	GTPase-activating protein CdGAPr isoform X3, GTPase-activating protein CdGAPr isoform X3, GTPase-activating protein CdGAPr isoform X1, GTPase-activating protein CdGAPr isoform X2, GTPase-activating protein CdGAPr isoform X4
LOC101746142	72.8380944055175	-2.63472893277812	0.00902367474811028	XP_062529484.1, XP_012546045.1	uncharacterized protein LOC101746142
LOC105841286	352.850979692729	-2.18365897373635	0.0093534593001926	XP_062528212.1, XP_012543838.1, XP_012543837.2	protein cereblon isoform X3, protein cereblon isoform X2, protein cereblon isoform X1
LOC119629960	63.7515429290641	-3.51562476928297	0.00951087712222717		uncharacterized LOC119629960
LOC101744148	979.56646302975	-2.1277769051075	0.00973761140024418	XP_062531184.1	glutamate receptor ionotropic, kainate 3 isoform X1
LOC134201416	81.4395912634545	-2.94789842785092	0.00979318831105703	XP_062532165.1	uncharacterized protein LOC134201416
LOC105841518	1979.98175088031	-1.72666902776475	0.0101344690894058	XP_062525562.1	uncharacterized protein LOC105841518
LOC101744434	56.5011073644321	-3.30458829988689	0.010285605855122	XP_012551440.3	uncharacterized protein LOC101744434
LOC134200064	734.731921978832	-1.49163114935044	0.010285605855122	XP_062527976.1	uncharacterized protein LOC134200064
LOC134200537	49.5842329130321	1.5076889382804	0.010285605855122		uncharacterized LOC134200537
LOC105841612	350.739873991521	-2.84438453248841	0.0103039849749757	XP_062530430.1, XP_012545700.1	protein 4.1 homolog isoform X1, protein 4.1 isoform X2
LOC101741472	387.379309636129	-2.40095131449288	0.0103367062226315	XP_062529262.1, XP_062529263.1	thrombospondin type-1 domain-containing protein 7A
LOC101741559	1431.75141135555	-1.5436822486382	0.0104590716674003	XP_037876789.2, XP_037876790.2, XP_037876791.2, XP_037876788.2, XP_062532432.1	zinc finger protein 628 isoform X2, zinc finger protein 135 isoform X3, zinc finger protein 628 isoform X4, zinc finger protein 668 isoform X1, zinc finger protein 358 isoform X5

LOC134198893	26.2657115732139	-2.36766803805601	0.0105461006482115		uncharacterized LOC134198893
LOC101742389	68.8792117197644	-3.36056629734853	0.0106771573141153	XP_004925487.1, NP_001040228.2	aminoacylase isoform X1, aminoacylase
LOC101741575	2020.76535989305	-1.68069823163713	0.0109073660883859	XP_037866957.1	BOS complex subunit NOMO1
LOC692416	620.275414163487	1.07949221490367	0.0110903857902836	NP_001036872.1	Bombyrin precursor
LOC101743730	31.2878845152587	-1.90306483502393	0.011118531329679	XP_004925496.2	GS homeobox 1
LOC101744584	26.8271908658572	-3.08253018657436	0.0111416150241279	XP_037869734.2	band 7 protein AGAP004871
LOC110384725	1582.89171341999	-1.56273075012647	0.0112825214838587	XP_037875396.1	WASH complex subunit 2
LOC101736035	341.893541663162	-1.65214929819553	0.0113891660635454	XP_037869826.1, XP_062527279.1, XP_062527280.1	zinc finger protein 658B X1, zinc finger protein 287 X2, zinc finger protein 37 X3
LOC134200472	459.366138125519	-2.7933055063503	0.0113891660635454		
LOC101744388	491.341249743503	-2.47073574019964	0.0114474592652168	XP_004933079.2	heparanase
LOC134201672	23.1438473784381	-4.1977344771441	0.0114474592652168	XP_062532898.1	uncharacterized LOC134201672
LOC101745631	963.147995864242	-1.10313789439941	0.011447813901631	XP_012550917.2, XP_012550914.2, XP_062525712.1, XP_021207643.2, XP_012550916.2	rho guanine nucleotide exchange factor 10 isoform X2, rho guanine nucleotide exchange factor 10 isoform X1, rho guanine nucleotide exchange factor 10 isoform X5, rho guanine nucleotide exchange factor 10 isoform X4, rho guanine nucleotide exchange factor 10 isoform X3
LOC105842452	1309.92486625386	-2.40516038191871	0.0115100885300519	XP_012551085.2	serine/arginine repetitive matrix protein 1
LOC101735699	8405.29698306875	-2.5089168736096	0.0116359218558397	XP_004928591.2, XP_012547877.1, XP_004928592.1, XP_012547878.1	dynamin isoform X5, dynamin isoform X1, dynamin isoform X6, dynamin isoform X4
LOC101745282	31.4282030869465	-2.74073695372075	0.0116727385411735	XP_012545356.3	uncharacterized LOC101745282
LOC101746656	1386.38347556678	-1.1901603125124	0.0116899492864272	XP_004924183.1, XP_062527021.1	protein virilizer isoform X1, protein virilizer X2
LOC101738826	16.0245145129823	3.28895821304215	0.0117078790996682	XP_062531067.1	uncharacterized protein LOC101738826
LOC101740565	1777.95834584129	-2.60185210101729	0.0117194004828776	XP_004932855.1	Krueppel-like factor luna X1
LOC134198974	22.3735299782127	-2.48572991509661	0.0117194004828776		
LOC101742637	51.3814520488942	-4.34120381849589	0.0117582765664952	XP_062531139.1	aminoacylase-1
LOC101736300	18167.862279593	-2.58010399828728	0.0118993953230745	XP_037867536.1, XP_037867535.1,	saccharopine dehydrogenase-like oxidoreductase isoform X1, saccharopine dehydrogenase-like oxidoreductase

				XP_037867539.1, XP_037867537.1	isoform X1, saccharopine dehydrogenase-like oxidoreductase isoform X2, saccharopine dehydrogenase-like oxidoreductase isoform X1
LOC134200214	13934.6434108173	-2.35443715636794	0.0118993953230745		
LOC101745226	4266.99813729871	-1.87272559946699	0.0118993953230745	XP_037874715.1, XP_037874716.1	uncharacterized protein LOC101745226 isoform X1, uncharacterized protein LOC101745226 isoform X2
LOC101741919	318.302412767501	-1.0022198336662	0.0119168287015637	XP_062529690.1, XP_004928309.1	proline-rich protein 11 X2, putative uncharacterized protein DDB_G0285869 isoform X1
LOC105841794	3242.05023181214	-2.29216658285551	0.0119270814301885		
LOC119630270	18.0599587294769	-4.83361871796505	0.0119702873583303	XP_037875005.2	uncharacterized protein LOC119630270
LOC101746708	558.494750438714	-2.48361086506342	0.0121601070397663	XP_004927115.1	uncharacterized protein LOC101746708
LOC101744272	14.9074933602306	-2.3403564276675	0.0122418434584219	XP_021205480.3	odorant receptor 4
LOC134199283	296.842533336681	-2.92753637249345	0.0125087316901003		
LOC101744888	1369.41258830242	-1.40174596624541	0.0125652780801772	XP_037874853.1	basic proline-rich protein
LOC101738715	177.883765187835	-2.75218914904962	0.0127950641065748	XP_021205283.1, XP_004928782.1	PDZ domain-containing protein 8 isoform X2, PDZ domain-containing protein 8 isoform X1
LOC101736634	146.878851761733	-2.91017591836247	0.0127975243874383	XP_037875083.1, XP_062531223.1	mucin-2 isoform X2, uncharacterized protein LOC101736634 isoform X1
LOC101735537	2731.08682564168	-1.40871524400341	0.0128078680126174	XP_037869167.1	intermembrane lipid transfer protein VPS13D
LOC134199848	13331.0124401161	-2.38195213842468	0.0128601965325698	XP_062527493.1	uncharacterized protein LOC134199848
LOC105841689	94792.9679710658	-2.89948303400505	0.012888512362529	XP_037876868.1	nuclear factor 1 B-type
serpin-13	2197.77322528272	-1.69179787505133	0.0128895863683194	XP_037870170.1, XP_037870171.1, XP_037870172.1	serine protease inhibitor 13 isoform X1, serine protease inhibitor 13 isoform X2, serine protease inhibitor 13 isoform X2
LOC101743340	25.5597320537936	-2.71284092941413	0.0128895863683194	XP_021204150.3	uncharacterized LOC101743340
CPFL4	85.378286521954	-2.30408267685892	0.0130313877255284	NP_001157376.1	cuticular protein CPFL family 4 precursor
Notch	1382.89146079353	-1.34166033724705	0.013072706481714	NP_001157370.1	neurogenic locus Notch protein precursor
LOC101739015	207.00209467232	-2.34542689048465	0.013072706481714	XP_062528596.1	cyclic nucleotide-gated cation channel alpha-3
LOC101737811	676.978340808808	-1.12977074799548	0.013072706481714	XP_012544774.2, XP_004924908.2	uncharacterized protein LOC101737811 isoform X2, uncharacterized protein LOC101737811 isoform X1
LOC101744789	53864.4601654127	-2.62908026947436	0.0132891691969846	XP_062528121.1,	ankyrin repeat and LEM domain-containing protein 2 homolog isoform X2,

				XP_004925434.3	ankyrin repeat and LEM domain-containing protein 2 homolog isoform X1
LOC101740269	513.888211427726	-1.41605886935772	0.013422100458671	XP_004924414.3, XP_037876027.1, XP_037876034.1	uncharacterized protein LOC101740269 isoform X1, uncharacterized protein LOC101740269 isoform X2, uncharacterized protein LOC101740269 isoform X3
LOC134199310	13752.1362350938	-2.34756679141588	0.0134557522046971	XP_062525904.1	uncharacterized protein LOC134199310
LOC119630126	27.2390347638098	-2.73165651009937	0.0134557522046971		
LOC119629867	25.8531040798757	-4.36605055755812	0.0135956418824302		
LOC134200829	13468.1028789202	-2.36315990261803	0.0137827052653162		
LOC134199117	107.377409291789	-2.05904738134409	0.0141747878610368		
LOC134198919	130.903340018558	-2.39639256423284	0.0141747878610368		
LOC101747048	4822.10942362937	-2.75833353990643	0.0141747878610368	XP_037869242.1	protein hobbit
LOC110385542	22.3669585211695	-2.79478841936261	0.0141747878610368		
LOC101744936	3057.17257374542	-1.65659668839068	0.0141747878610368	XP_004926203.1	filaggrin-2
LOC134201115	161.422912719426	-2.4143907154516	0.0141747878610368	XP_062531293.1	metabotropic glutamate receptor 1-like
LOC134199843	13162.267162471	-2.37399777886888	0.0143100980422549	XP_062527420.1	uncharacterized LOC134199843
LOC101744927	1128.90696976875	-1.88217883047389	0.0143439665817393	XP_062528211.1, XP_037871196.1, XP_037871195.1, XP_037871193.1	kinesin-like protein Klp68D isoform X4, kinesin-like protein Klp68D isoform X3, kinesin-like protein Klp68D isoform X2, kinesin-like protein Klp68D isoform X1
LOC134199774	344059.411837752	-1.98424333440937	0.0143546963306314		
LOC693049	1340.77275091485	-2.52609144868835	0.0145668330783442	XP_062528330.1, NP_001037492.1	tubulin beta chain isoform X1
LOC134201539	13063.0529692627	-2.3729215567481	0.0145850806706869		
LOC134199223	13127.1140420855	-2.36923455733428	0.0146210406554562		
LOC101745037	243.790988700147	-2.53412733452889	0.0146210406554562	XP_062531838.1, XP_037876023.1	uncharacterized protein LOC101745037 isoform X1, cytoplasmic dynein 2 intermediate chain 1 isoform X2
LOC101741626	431.559452508094	-1.90076138112488	0.0146343240059786	XP_004926256.1	carboxypeptidase N subunit 2
LOC101743372	23.0665407631047	-1.62361583794541	0.0147148505518637	XP_004930864.4	43 kDa receptor-associated protein of the synapse homolog
RnrS	525.407279659907	1.97178213831338	0.0148900135031764	NP_001244266.1	ribonucleoside-diphosphate reductase subunit M2
LOC119631096	439039.322250691	-1.93497469435912	0.0149554664641333		
LOC119631030	439103.802094644	-1.93481665242663	0.0149554664641333		
LOC134199775	438416.934790958	-1.935567572365	0.0149554664641333		

LOC105842387	13417.4385002172	-2.33973378260426	0.0149554664641333	XP_012550529.2, XP_037869725.1	uncharacterized protein LOC105842387 isoform X2, uncharacterized protein LOC105842387 isoform X1
LOC101741117	92.8919314651057	-1.48087236003395	0.014957602514481	XP_062530694.1	small conductance calcium-activated potassium channel protein
LOC101745400	676.295840600917	-1.25109504389982	0.0149686117098467	XP_062527193.1, XP_062527196.1, XP_062527197.1, XP_037869634.1, XP_062527194.1, XP_062527195.1, XP_062527198.1, XP_062527199.1	F-actin-uncapping protein LRRC16A isoform X1, F-actin-uncapping protein LRRC16A isoform X4, F-actin-uncapping protein LRRC16A isoform X5, F-actin-uncapping protein LRRC16A isoform X3, F-actin-uncapping protein LRRC16A isoform X2, F-actin-uncapping protein LRRC16A isoform X2, F-actin-uncapping protein LRRC16A isoform X6, F-actin-uncapping protein LRRC16A isoform X6
LOC134201258	228.049528434975	-3.85982352122947	0.015109525918738		
LOC101737173	1021.65363284677	-1.28521697464737	0.015109525918738	XP_012551705.1	attractin-like protein 1
LOC101747185	109.747465496894	-2.54155806193125	0.015111602497558	XP_062524799.1, XP_037866931.1, XP_062524800.1, XP_062524801.1	uncharacterized protein LOC101747185
LOC101737705	555.462858814159	-1.38026436599683	0.015111602497558	NP_001296558.1	dnaJ homolog subfamily C member 16 l(3)80Fg precursor
LOC134201428	13461.4395024492	-2.33641125273748	0.0152239500230015		
LOC101745340	3329.85323407668	-1.89259390860257	0.0153348495112681	XP_004928244.1, XP_062529637.1	uncharacterized protein LOC101745340 isoform X1, uncharacterized protein LOC101745340 isoform X2
LOC134198816	13136.9828369108	-2.3596966578623	0.01534547418862	XP_062524332.1	uncharacterized protein LOC134198816
LOC101745219	224.907392594558	-1.04182371747703	0.0154967256566765	XP_062524819.1, XP_037867031.2	oxysterol-binding protein-related protein 1 isoform X2, oxysterol-binding protein-related protein 1 isoform X1
LOC101737632	245.753339272569	-1.63850143559506	0.0154967256566765	XP_021204138.1	out at first protein
LOC134199488	47.6012665254989	-2.40244505636935	0.0155952968950252		
LOC134200159	18.161193256935	-2.73701172382495	0.01564453735076		n.a
LOC119628735	13470.6612386759	-2.33529012928609	0.0157022884629025		
LOC101743980	52.9485102385442	-2.5202835642981	0.0158203147485689	XP_004927710.2	facilitated trehalose transporter Tret1-like
LOC778525	145.153032172428	-1.44768369120795	0.0158508314315455	XP_012546644.1, NP_001091819.1	putative defense protein isoform X1, putative defense protein precursor
LOC101744698	26.3291116138891	-1.83482360926889	0.0158508314315455	XP_037874312.1	ankyrin repeat domain-containing protein 29

LOC692993	190.65016049039	-2.04465746346197	0.0160366861595346	XP_062527299.1, NP_001040295.1	guanine nucleotide binding protein isoform X4, guanine nucleotide binding protein
LOC101738080	219.331660476594	-2.49651422120399	0.0162246092999725	XP_012545336.2, XP_004925605.2	insulin-like receptor
LOC101742925	21794.5639200822	-2.29490184493056	0.0162246092999725	XP_062531569.1, XP_062531570.1, XP_062531573.1, XP_062531571.1, XP_062531572.1, XP_062531568.1	uncharacterized protein LOC101742925 isoform X1, uncharacterized protein LOC101742925 isoform X1, rho guanine nucleotide exchange factor 11 isoform X4, uncharacterized protein LOC101742925 isoform X2, rho guanine nucleotide exchange factor 11 isoform X3, uncharacterized protein LOC101742925 isoform X1
LOC101743656	181.056336989026	-1.31167730120206	0.0162246092999725	XP_037876490.1	protein fem-1 homolog A
LOC119631145	294405.769285353	-1.79458906544044	0.0164544824946091		
LOC101737382	311.094352737051	-1.01333706058867	0.0164544824946091	XP_004921732.2	toll-like receptor 7
LOC119631080	389590.921256126	-1.79470119525456	0.0165110479875297		
LOC119630962	390233.110920392	-1.79336688789919	0.0165359084051122		
LOC110386771	178.13375839881	-2.52388099517173	0.016701709138121		
LOC119630076	54.7720877620317	-2.86247228371487	0.0170046701790395	XP_037874130.1	uncharacterized protein LOC119630076
LOC119630907	383211.890529393	-1.7937869322616	0.0171228402358855		
LOC119630906	383213.87131525	-1.79384816527814	0.0171228402358855		
LOC101746060	66.1223462680633	5.08484299294557	0.0171228402358855	XP_021205730.1, XP_021205731.1, XP_021205728.1	coiled-coil domain-containing protein 191 isoform X1, coiled-coil domain-containing protein 191 isoform X1, coiled-coil domain-containing protein 191 isoform X1
LOC134200390	397.781832060924	-2.15127045085315	0.0171228402358855		
LOC119630900	288698.066686816	-1.79241853105683	0.0172808531415666		
LOC101743101	618.339829893495	-1.92880535099144	0.0173631703514226	XP_004922519.1	glucose transporter GlcP
LOC134199776	388963.855263835	-1.78785350935138	0.0175050911587705		
LOC134199777	293031.516638808	-1.78764212314969	0.0175050911587705		
LOC101744293	1217.92161534137	-1.71052340815101	0.0175955751694338	XP_012551938.1	growth arrest-specific protein 1
LOC101739146	231.894088456375	-1.16492491183926	0.0176597560551345	XP_004931867.1	probable G-protein coupled receptor CG31760
LOC119630968	382500.117407216	-1.78762531478336	0.0176597560551345		
LOC119630875	383312.569791967	-1.78781371461127	0.0176597560551345		
LOC134199662	14.143247370491	-2.51379681206662	0.0177122697421053		

LOC101740711	1303.35524593272	1.90239336236457	0.0177122697421053	XP_062530058.1, XP_004922439.1	uncharacterized protein LOC101740711
LOC101744311	71.3340458520931	-2.49215128799129	0.0177122697421053	XP_012544193.2	NADH dehydrogenase [ubiquinone] 1 alpha subcomplex subunit 11
LOC134199143	70.1978419247271	-2.0985172371694	0.0177886667216662		
LOC119631029	288291.680482527	-1.78432180736455	0.0178685752239732		
LOC101744221	4143.94164548984	-2.07406107303881	0.0181012684349486	XP_062524493.1, XP_037867080.1, XP_037867079.1	bromodomain-containing protein 7 isoform X6, bromodomain-containing protein 7 isoform X3, bromodomain-containing protein 7 isoform X2
Cyp4l6	34.2931151976656	2.2271591206333	0.0181294077281561	NP_001108343.1	cytochrome P450 CYP4L6 precursor
LOC101737404	135859.380560061	-2.14933548646641	0.018149418572149	XP_004929343.2, XP_012548408.1, XP_021205630.1, XP_012548410.1, XP_062524062.1	pseudouridylate synthase RPUSD2 isoform X2 pseudouridylate synthase RPUSD2 isoform X2, pseudouridylate synthase RPUSD2 isoform X1, pseudouridylate synthase RPUSD2 isoform X3, pseudouridylate synthase RPUSD2 isoform X3
LOC105841430	16.9443868156487	2.94981439192406	0.018149418572149	XP_062526345.1	uncharacterized protein LOC105841430
LOC119630003	26.8050303077044	-1.62483980746447	0.018149418572149	XP_062530118.1	serine/arginine repetitive matrix protein 1-like
LOC134201117	2670.42662441633	-2.41871843382568	0.018149418572149		
LOC101742428	1765.54924062532	-1.92470711058622	0.018197356105578	XP_037870494.1	transportin-3
LOC101740975	356.064641260827	-2.53971379930932	0.0183901762797065	XP_062524089.1	protein eyes shut
LOC101741105	116.370201129707	-1.17887760404621	0.01840531497605	XP_062530427.1, XP_062530426.1, XP_021203655.1, XP_062530425.1	myrosinase 1 isoform X2, myrosinase 1 isoform X2, myrosinase 1 isoform X1, myrosinase 1 isoform X2
LOC101739475	320.379158773973	-1.84763136880334	0.01840531497605	XP_062530924.1, XP_062530923.1, XP_037874770.2, XP_062530917.1, XP_062530922.1, XP_062530921.1, XP_062530919.1, XP_062530918.1, XP_062530916.1,	protein still life isoform SIF type 1 isoform X7, protein still life isoform SIF type 1 isoform X6, protein still life isoforms C/SIF type 2 isoform X10, protein still life isoform SIF type 1 isoform X1, protein still life isoform SIF type 1 isoform X5, protein still life isoform SIF type 1 isoform X4, protein still life isoform SIF type 1 isoform X3, protein still life isoform SIF type 1 isoform X2, protein still life isoform SIF type 1 isoform X1,

				XP_062530925.1,<br>XP_062530926.1	protein still life isoforms C/SIF type 2 isoform X8,<br>protein still life isoforms C/SIF type 2 isoform X9
LOC134201491	9.81829267699277	-2.82978950503835	0.01840531497605		
cce-6	506.784200883945	1.25185564203049	0.0185736532476313	NP_001182392.1	carboxyl/cholinesterase 6 precursor
BmGEMININ	414.356258615787	-1.56814693492326	0.019225478625536	NP_001244268.1	geminin DNA replication inhibitor
LOC101741401	236.684295172834	-2.04940818793136	0.0195589726688284		
LOC101742063	421.587287144136	-1.53241467895626	0.0196754899698905	XP_012547176.1,<br>XP_012547177.1,<br>XP_021204766.1	uncharacterized protein LOC101742063 isoform X1,<br>uncharacterized protein LOC101742063 isoform X3,<br>uncharacterized protein LOC101742063 isoform X2
LOC134201373	1788.13741297759	-1.54956531903314	0.0199116557273924		
LOC100529205	49.2929436034695	1.40163796987229	0.0199188851441003	XP_012550619.1,<br>XP_012550618.1	putative inorganic phosphate cotransporter
LOC101746458	1662.23457309723	-2.15672457354224	0.0200609301653681	XP_004922281.1	regulator of G-protein signaling 7
gas2	487.64108186262	-1.14573610603707	0.0201318318313019	XP_012543811.1,<br>XP_012543814.1,<br>XP_012543810.1,<br>XP_012543813.1,<br>XP_012543815.1,<br>XP_012543812.1,<br>NP_001189461.1	growth arrest-specific protein 2 isoform X1,<br>growth arrest-specific protein 2 isoform X1,<br>growth arrest-specific protein 2 isoform X1,<br>growth arrest-specific protein 2 isoform X1,<br>growth arrest-specific protein 2 isoform X1,<br>growth arrest-specific protein 2 isoform X1,<br>growth arrest-specific protein 2
LOC101747166	725.796562231758	-2.16382809654095	0.0202982171979364	XP_037871331.1,<br>XP_037871329.1,<br>XP_037871325.1,<br>XP_037871323.1	Neogenin isoform X4,<br>Neogenin isoform X3,<br>Neogenin isoform X2,<br>Neogenin isoform X1
LOC101735930	1013.11403054782	-2.33487401232915	0.0203373705217467	XP_037877672.1,<br>XP_037877674.1	dedicator of cytokinesis protein 7 isoform X1,<br>dedicator of cytokinesis protein 7 isoform X2
LOC101744796	152.99409462819	-1.32572692302191	0.0203911526595248	XP_037870382.1,<br>XP_037870381.1,<br>XP_037870380.1,<br>XP_037870383.1	protein nubbin isoform X3,<br>protein nubbin isoform X2,<br>protein nubbin isoform X1,<br>protein nubbin isoform X4
CPR9	108.967426461452	-1.96491068413893	0.0203911526595248	NP_001166739.1	cuticular protein RR-1 motif 9 precursor
LOC101737495	124.48681155893	-2.82689139025368	0.0204861026239644	XP_062527815.1	uncharacterized protein LOC101737495
LOC110385628	395.941358867436	-2.23576313682458	0.0204861026239644		

LOC101736340	36.349756818102	-2.53437917840745	0.0204861026239644	XP_062529350.1	uncharacterized protein LOC101736340
LOC101745633	11.6597214823854	-2.57687733737317	0.0210199999131196	XP_012551291.1	heparan sulfate glucosamine 3-O-sulfotransferase 3B1
LOC101738867	199.43736758626	-1.54578649140597	0.0214882913528615	XP_037872708.1	uncharacterized protein LOC101738867
LOC105842004	608.881094639231	-1.99484464795431	0.0214882913528615	XP_037871901.1	uncharacterized protein LOC105842004
LOC101735646	82.4693777649057	-2.09494867083267	0.0214882913528615	XP_021203764.2	1-phosphatidylinositol 4,5-bisphosphate phosphodiesterase epsilon-1
LOC101743883	25565.5924122395	-1.3133517863169	0.0217662838905488	XP_021203587.1, XP_004926063.1	uncharacterized protein LOC101743883 isoform X1, uncharacterized protein LOC101743883 isoform X2
LOC101739615	6023.30045316271	-2.2486935104252	0.0218160602467193	XP_021207985.2, XP_021207984.2	adenylate cyclase type 6
LOC101737853	447.562180150015	-1.88495054839838	0.0218160602467193	XP_012543976.2, XP_012543977.2	oocyte zinc finger protein XlCOF6 isoform X1, zinc finger protein 567 isoform X2
LOC105841350	25.3090332204598	-2.80354041971549	0.0218160602467193	XP_012544221.3, XP_037875030.1	uncharacterized protein LOC105841350 isoform X1, uncharacterized protein LOC105841350 isoform X2
LOC100862826	26.5702318393975	-3.96038380637302	0.0219842225653807	NP_001243976.1	atonal
LOC101740498	29.1487781253102	-2.51802838758703	0.022233576349769	XP_004926110.1	uncharacterized protein LOC101740498
LOC134198918	209.246864326287	-2.87187145732399	0.0226615167082298		
LOC134199111	14.8024898330678	-2.84883762507941	0.0231463337557087	XP_062525269.1	uncharacterized protein LOC134199111
LOC101741875	2996.25427419152	-2.8418124393539	0.0234636891373867	XP_004928635.1	ras-related protein Rab-9B
LOC105842429	20.7265138992418	-2.46039378059229	0.0238386322007038		
LOC119628780	15.9704325158542	3.03691533061267	0.0241060886540537	XP_062525690.1	ATP-dependent DNA helicase pif1
LOC119628535	56.5968707954433	-1.46597344450668	0.0241706732501752	XP_037867025.1	piggyBac transposable element-derived protein 4 isoform X1
LOC105842223	93.3998676487693	-2.69583887864749	0.0246595088596678		
LOC101745201	139.04797925258	-2.71346850203878	0.0247082529849587	XP_021207833.1	neprilysin-4 isoform X1
LOC119630755	23.372010701771	-2.15279800955705	0.0247866514595666	XP_062530458.1	uncharacterized protein LOC119630755
LOC101745524	104.333857334174	-1.01535128202125	0.0249799218689127	XP_037867121.1, XP_037867119.1, XP_037867123.1	uncharacterized protein LOC101745524 isoform X3, uncharacterized protein LOC101745524 isoform X1, uncharacterized protein LOC101745524 isoform X5
LOC732884	93.8412115172897	2.76486098038052	0.0251095229083505	XP_012549129.1	trypsin-like protease isoform X1
LOC101736126	2185.86106493912	-2.21902983370828	0.0251095229083505	XP_037869307.1, XP_021209163.1, XP_012553012.1	TBC1 domain family member 9 isoform X1, TBC1 domain family member 9 isoform X1, TBC1 domain family member 9 isoform X2

LOC101739624	40.2599962091489	-1.52987696530141	0.0251095229083505	XP_021208931.2, XP_021208926.2, XP_021208923.2	Bardet-Biedl syndrome 1 protein isoform X2, Bardet-Biedl syndrome 1 protein isoform X2, Bardet-Biedl syndrome 1 protein isoform X1
LOC101743406	1157.73301017103	-2.18883117597131	0.0251095229083505	XP_004928320.1	galactosylgalactosylxylosylprotein 3-beta-glucuronosyltransferase I
SGF3	528.854682271817	-1.79286992638613	0.0252383860928612	NP_001037456.2	silk gland factor 3
LOC101747115	359.893281631347	-1.09748363097296	0.0254415242314077	XP_037868288.1, XP_037868289.1	WAS/WASL-interacting protein family member 1 isoform X1, uncharacterized protein LOC101747115 isoform X2
LOC101745593	281.377486675464	-1.70792031197623	0.0255876013570111	XP_004933542.1	la-related protein 6
LOC101744799	2094.46234312607	-3.82586915817328	0.0257386454957562	XP_021204789.1, XP_062527713.1	iduronate 2-sulfatase
LOC101738933	1541.48338832713	-2.59410870448136	0.0258218203326439	XP_004925401.1	forkhead box protein J2
LOC101745829	14785.6464866854	-1.86016336799879	0.0258218203326439	XP_037871823.1	cell division cycle 7-related protein kinase
LOC119630248	18.4749062894482	-2.52387751216234	0.026043987572994	XP_062531086.1	serine/arginine repetitive matrix protein 1-like
LOC101740721	2486.99907786777	-1.54483434329164	0.0260536023224296	XP_037869877.1, XP_062527010.1	uncharacterized protein LOC101740721 isoform X1, uncharacterized protein LOC101740721 isoform X2
LOC101740681	623.696406209851	3.36192482504278	0.0262455468871573	XP_062527308.1	probable peptidoglycan muropeptide transporter SLC46 isoform X1
Sxl	2220.06880996928	-2.32305884251538	0.0265678212615689	XP_012548066.2, NP_001036780.1, NP_001166854.1	sex-lethal isoform X1, sex-lethal isoform L, sex-lethal isoform S
LOC110384752	44.3729258334501	-2.51140270483677	0.0267651211490434	XP_037868952.2	uncharacterized protein LOC110384752
LOC101737266	2034.42290470104	-1.12541420292348	0.0268150199794176	XP_012550319.2, XP_062525344.1	activating signal cointegrator 1 complex subunit 3 isoform X2, activating signal cointegrator 1 complex subunit 3 isoform X1
LOC105842044	9.05083100740159	-2.91713219989586	0.0268150199794176	XP_037872521.1, XP_062528853.1, XP_062528854.1, XP_062528855.1	O-acyltransferase like protein isoform X2, O-acyltransferase like protein isoform X1, O-acyltransferase like protein isoform X3, O-acyltransferase like protein isoform X4
LOC101735551	175.721494239089	-1.85585694994933	0.0268150199794176	XP_012545644.1, XP_012545645.1, XP_037873106.1,	regulator of G-protein signaling 19 isoform X1, regulator of G-protein signaling 20 isoform X2, regulator of G-protein signaling 20 isoform X2,

				XP_004925941.1,<br>XP_062529538.1,<br>XP_012545647.1	regulator of G-protein signaling 19 isoform X1,<br>regulator of G-protein signaling 20 isoform X2,<br>regulator of G-protein signaling 20 isoform X3
LOC105842190	76.7415270355378	3.30087045345691	0.0270043354813913	XP_012549169.3	trypsin, alkaline C-like
LOC101739713	2075.02182058597	2.00396517600093	0.0270043354813913	XP_004922431.1	uncharacterized protein LOC101739713
LOC101738588	708.546803161102	4.23200477577168	0.0272597305184532	XP_012550567.3	arylphorin subunit beta
LOC119629125	161.315997203877	-2.85072686612403	0.0276367167826919	XP_037869891.1	uncharacterized protein LOC119629125
LOC134201127	25.4837325985787	-2.35217160091083	0.0276367167826919		
LOC100301511	12428.2184148275	-2.74632351202054	0.0276367167826919	XP_062530133.1,<br>XP_012545120.1,<br>NP_001153678.1	uncharacterized protein LOC100301511 isoform X2,<br>uncharacterized protein LOC100301511 isoform X1,
LOC101742582	1204.0141296885	-1.40153502338984	0.0276367167826919	XP_037876776.1	rho GTPase-activating protein 190 isoform X1
LOC101743348	631.128888853654	-1.46738990222585	0.0277697748348014	XP_062528192.1,<br>XP_062528193.1,<br>XP_062528195.1,<br>XP_062528196.1,<br>XP_062528191.1,<br>XP_062528194.1	protein Fe65 homolog isoform X2,<br>protein Fe65 homolog isoform X2,<br>protein Fe65 homolog isoform X2,<br>protein Fe65 homolog isoform X2,<br>protein Fe65 homolog isoform X2,<br>protein Fe65 homolog isoform X2,
LOC101739725	268.093508779757	-2.27745379270206	0.0277979101260666	XP_062528803.1	protocadherin-16
LOC105841270	66.5531862140017	-1.95481689225748	0.0280398879030866	XP_037875063.1,<br>XP_037875062.1,<br>XP_037875067.1	uncharacterized protein LOC105841270 isoform X2,<br>uncharacterized protein LOC105841270 isoform X1,<br>uncharacterized protein LOC105841270 isoform X3
LOC119630943	444051.019924011	-1.88052371048812	0.0282756324534307		
LOC101742593	260.968803902592	-1.30096620776452	0.0284291131482425	XP_012545937.1	frizzled-4
LOC101741109	245.219935293514	-1.7973033821838	0.0285231699653944	XP_004927779.1	histone deacetylase complex subunit SAP30 homolog
LOC134199773	446279.303248734	-1.87700558642049	0.0285540472591094		
LOC119630880	446447.688094151	-1.87675564310045	0.0285540472591094		
LOC101739495	172.275240710071	-2.20325901467233	0.029018777406433	XP_004924216.1	uncharacterized protein LOC101739495 isoform X1
LOC101735593	69.973855369878	-2.15438988555886	0.0290886498255494	XP_004924057.3	uncharacterized protein LOC101735593
LOC101738114	6460.03591465236	-1.47901346475055	0.0291358125737293	XP_021201900.1,<br>XP_012553326.1,<br>XP_004923606.1	mucin-2 isoform X1,<br>mucin-2 isoform X2,<br>mucin-2 isoform X1
LOC119630467	85.7788019711351	-2.77147653163646	0.0291829264878015		

LOC101738225	915.371877922296	-1.01379146700655	0.0292551698902907	XP_037866684.1,<br>XP_004930877.2	uncharacterized protein LOC101738225 isoform X1,<br>uncharacterized protein LOC101738225 isoform X2
LOC101745459	205.336034920401	-2.10451764974251	0.0292746591119699	XP_004923379.1	apolipoprotein D
LOC101743453	244.553544648618	-1.26771753391309	0.0294695635805693	XP_012545879.1,<br><br>XP_021203730.1	solute carrier organic anion transporter family member 4A1 isoform X1,<br>solute carrier organic anion transporter family member 4A1 isoform X2
LOC101743412	163.534633152037	1.09600086513578	0.029715196570708	XP_004929302.1	phosphatidylserine synthase
LOC101745630	2151.26744821229	-1.7104163204931	0.029837476757858	XP_021206998.2,<br>XP_021206999.2	PH and SEC7 domain-containing protein isoform X3,<br>PH and SEC7 domain-containing protein isoform X6
LOC101743480	226.02498963597	-2.43111058205321	0.0298838234967382	XP_012553314.1,<br>XP_062531154.1	circadian clock-controlled protein daywake,
LOC101744365	3589.29163296078	-2.33037325731008	0.0300411436477434	XP_037867745.1	heterogeneous nuclear ribonucleoprotein H
lasp	1591.57662145771	-1.72606632899797	0.0301145681414764	NP_001239601.1	LIM and SH3 domain protein Lasp
LOC110385540	13.1404708821559	-2.29957665241601	0.0303242812938797		
LOC119629360	20.3936379651612	-3.01132751822372	0.0304571576840736	XP_037870940.1	trypsin-2
LOC105841600	16095.1411126315	-2.35077728716469	0.030560956545256		
Mir79	249.281282582299	-2.97765460012784	0.0306263404563101		
Mir3406	19.6933729120135	-2.96212155326491	0.0306400756826396		
Or	314.88936250576	-1.0796228105063	0.0306400756826396	XP_037876685.1,<br>XP_012546201.2,<br>XP_012546200.2,<br>NP_001037020.1	nuclear orphan receptor isoform X3,<br>nuclear orphan receptor isoform X2,<br>nuclear orphan receptor isoform X1,<br>nuclear orphan receptor
LOC101745401	63.6431868520554	-2.8505534190896	0.0307391290804612	XP_004932101.1,<br>XP_037868428.1	cationic amino acid transporter 4,<br>cationic amino acid transporter 4
LOC101744550	836.50469148224	-1.07256987736801	0.0309678985199999	XP_037871846.1,<br>XP_037871847.1,<br>XP_021202636.2,<br>XP_062528599.1	transcription initiation factor TFIID subunit 6 isoform X3,<br>transcription initiation factor TFIID subunit 6 isoform X4,<br>transcription initiation factor TFIID subunit 6 isoform X2,<br>transcription initiation factor TFIID subunit 6 isoform X1
LOC101743161	157.819696945992	-1.55309144773912	0.0310761938609683	XP_062532438.1	ankyrin-2
LOC101736976	39.0189603520569	-2.05320245772203	0.0311084696482888	XP_004926292.2	cyclin-dependent kinase 5 activator 1

LOC101744396	62.8617748278497	-1.61604210734203	0.0314068924006504	XP_021209077.2, XP_062531659.1, XP_021209076.2	serologically defined colon cancer antigen 8 homolog isoform X2, serologically defined colon cancer antigen 8 homolog isoform X3, serologically defined colon cancer antigen 8 homolog isoform X1
LOC134200023	45.7716030834991	-2.25581375857329	0.0323568358814641		
LOC101737558	394.160767545295	-1.24663666737482	0.0325448040851163	XP_037873099.1	DNA repair protein XRCC1 X2
LOC101742530	85.6883031180019	-1.38543413048241	0.0326447545612044	XP_037867513.1, XP_037867515.1, XP_037867514.1, XP_037867516.1, XP_062525138.1	cubilin isoform X1, cubilin isoform X3, cubilin isoform X2, cubilin isoform X4, cubilin isoform X5
LOC119630964	354711.952717359	-1.9217657943966	0.0328848140392024		
LOC119630748	125.009338441627	-1.35809765999672	0.0333107698410506		
LOC101738207	28.7912247322469	-1.91454870164777	0.0334353846891681	XP_004925606.1	uncharacterized protein LOC101738207
LOC101740247	90.8326764125133	-2.68375004627732	0.0334353846891681	XP_062531334.1	ovochymase-1 X1
LOC134201679	50.6565805581461	-2.01039011373135	0.0334353846891681		
LOC101739307	101.002260361471	-1.74574732895299	0.0338342385919718	XP_004927001.2	lipase 1
LOC101740046	195.439975247097	-1.46306718849255	0.0338342385919718	XP_037869865.1, XP_037869867.1, XP_037869866.1	zinc finger protein 773 isoform X1, zinc finger protein 846 isoform X4, zinc finger protein 696 isoform X2
LOC119630093	37.7411748182345	-1.61091743009042	0.0338342385919718	XP_062530778.1	uncharacterized protein LOC119630093
LOC101737500	34.8888873186627	-1.68616289904814	0.0339461447194559	XP_004928434.1	G-protein coupled receptor moody
LOC134198781	202.5095831504	-2.16217547973099	0.0345356175063379		
LOC101741491	204.741283603134	-2.19123949251052	0.0346039142575255		
LOC101735390	277.192801445415	-1.90725101714661	0.035000511334921	XP_037872542.1, XP_037872539.1, XP_062529014.1	optomotor-blind protein isoform X10, optomotor-blind protein isoform X6, optomotor-blind protein isoform X9
LOC134199184	13.5358538988313	-2.05566928128423	0.0352413605067307		
LOC101739907	841.675503411005	-1.77125428233773	0.0353153320818989	XP_037871470.1	hamartin
LOC101742208	189.703726644901	-2.91855699492969	0.0355410721892641	XP_004926756.2	furin-like protease 2
Alg-2	4349.11693391066	-1.93311503493338	0.0358462336336576	XP_062527605.1, XP_012547119.1	apoptosis-linked protein 2 isoform X1, apoptosis-linked protein 2 isoform X2
LOC134199685	42.653897154927	-2.68353700067877	0.0359613610380083		

Or-59	64.9176650939721	-1.95895894694017	0.0361401186715207	NP_001166611.1	olfactory receptor 59
LOC105842471	154.575789942042	-2.22350562944296	0.0366368933865905	XP_021207812.1, XP_062529424.1, XP_021207813.1	uncharacterized protein LOC105842471 isoform X1, uncharacterized protein LOC105842471 isoform X2, uncharacterized protein LOC105842471 isoform X2
LOC101746432	408.47290570123	-1.4544047843886	0.0368124390358451	XP_062527804.1	teneurin-a isoform X2
LOC101740270	1915.53041452654	-1.87169128069407	0.0374377836433036	XP_004924483.2, XP_037871996.1, XP_062528782.1, XP_062528781.1	uncharacterized protein LOC101740270 isoform X1, uncharacterized protein LOC101740270 isoform X1, uncharacterized protein LOC101740270 isoform X2, uncharacterized protein LOC101740270 isoform X1
LOC101739274	182.556845286842	-2.14197230877278	0.037692916517087	XP_062528950.1, XP_062528954.1, XP_037872361.1	LIM/homeobox protein Lhx1 isoform X1, LIM/homeobox protein Lhx1 isoform X7, LIM/homeobox protein Lhx1 isoform X4
LOC101742225	218.375276415831	-2.94359996889629	0.037725522934355	XP_004930247.2	atlastin
LOC110386379	23.7847364654645	-2.15930444619501	0.037725522934355		
LOC101744134	399.24280286463	-1.42896615990691	0.0378058440921297	XP_037866543.1	ATP-dependent RNA helicase DHX33
LOC101742627	16.8323075973378	2.06819426939254	0.0378058440921297	XP_004923290.1	trypsin, alkaline A
LOC101744519	90.4808933420381	-1.02447906840891	0.038313718532352	XP_004929418.2	neutral ceramidase isoform X2
LOC119630321	31.6434102811371	-2.43608878970912	0.038313718532352		
LOC110384937	18.7341062608237	-2.43038115238968	0.0386106885942175		
LOC101741905	48144.3827600852	-2.73504667452708	0.0389834670407012	XP_004924018.1, XP_004924019.1	uncharacterized protein LOC101741905 isoform X1 uncharacterized protein LOC101741905 isoform X2
LOC101746290	871.043984757659	-1.15171438731803	0.0392076521376344	XP_004926768.1	vacuolar protein sorting-associated protein 53 homolog
LOC101737507	108.911787934065	-1.33375185277192	0.0392250602264311	XP_012549231.2, XP_037866760.1, XP_062524708.1, XP_062524707.1	uncharacterized protein LOC101737507 isoform X, uncharacterized protein LOC101737507 isoform X3, uncharacterized protein LOC101737507 isoform X4, uncharacterized protein LOC101737507 isoform X2
LOC101742972	67.7885392128836	-2.95718714606558	0.0392250602264311	XP_037868949.2	protein artichoke
LOC119629516	9.96631063916185	-3.35396452126626	0.0392250602264311	XP_037871467.1	neuropeptides capa receptor
LOC101745243	1179.20058643402	-1.15371577548541	0.0392250602264311	XP_037871747.1	uncharacterized protein LOC101745243 isoform X1
LOC101741498	52.6092060649291	1.78564899632407	0.0392513913957845	XP_004930242.1	monocarboxylate transporter 5
LOC134200822	62.0352701303313	-1.9875712689266	0.0400477323891472		
LOC101738472	927.045875068543	-1.40756757708898	0.0404902960350448	XP_004924705.1	gamma-tubulin complex component 3 homolog
LOC101747066	119.29203450175	-1.49658595944036	0.0410266245739515	XP_062524478.1	cell adhesion molecule Dscam2

LOC101744909	582.73508151707	-1.47079184335699	0.0417956902735471	XP_012551028.1, XP_012551028.1	zinc finger protein 628
LOC101744528	1322.15040891541	-1.74595816472284	0.0417956902735471	XP_021207453.1, XP_062530869.1	GATA-binding factor C isoform X3, GATA-binding factor C isoform X5
LOC101741257	50.1147487245084	1.57372899242288	0.0418868237895555	XP_004929080.3	zinc finger HIT domain-containing protein 2
LOC134201356	62.1063932512347	-2.26098981818956	0.0418868237895555		
LOC101738623	233.601326112764	-1.79869922553737	0.0424340807633816	XP_012548905.2	transcriptional repressor scratch 2
LOC110386066	61.3474240467952	-2.27172441337539	0.0425810896410466		
LOC101744662	76.8188966375862	-2.47972742353845	0.042679003956989	XP_021205301.1, XP_012547775.1, XP_062528359.1	homeobox protein HMX3 isoform X2, homeobox protein HMX3 isoform X2, homeobox even-skipped homolog protein 1 isoform X1
LOC692748	385.817099870607	-1.55258033562803	0.0427316425003958	XP_037868006.1	kinesin-like protein KLP2
LOC101744658	274.337998138125	-1.19392224454036	0.043964066801309	XP_004928152.1	uncharacterized protein LOC101744658
LOC134199254	45.7380176156556	-2.22837987651562	0.0444460545068634	XP_062525701.1	uncharacterized protein LOC134199254
LOC101739318	997.469649366057	-1.93433921243278	0.0448331916121982	XP_012548141.3	SANT and BTB domain regulator of class switch recombination
LOC119630274	51.0877246903764	-2.27060524500761	0.0449644494011023	XP_062530823.1	uncharacterized protein LOC119630274
LOC101738342	465.66811364682	-1.3807344631618	0.0450729987711707	XP_037873953.1	uncharacterized protein LOC101738342
LOC134199822	47.0289418546657	-2.14822002431015	0.0460308536717921	XP_062527378.1	uncharacterized protein LOC134199822
rdl1	39.2494668190002	-1.90451284251932	0.0462614074051538	NP_001093294.1, XP_037868154.1, XP_037867822.1, XP_021205972.2	GABA-gated chlorine channel alpha subunit precursor, GABA-gated chlorine channel alpha subunit isoform X3, GABA-gated chlorine channel alpha subunit isoform X2, GABA-gated chlorine channel alpha subunit isoform X1
LOC101745815	375.885973855927	-2.09090789089995	0.0462614074051538	XP_004926208.1, XP_062529830.1	general transcription factor IIH subunit 4 isoform X3, general transcription factor IIH subunit 4 isoform X1
LOC105841361	44.2441513274047	-4.15707885099706	0.0470304553509712	XP_037877168.1, XP_021202450.2	uncharacterized protein LOC105841361 isoform X1, uncharacterized protein LOC105841361 isoform X2
LOC101736349	1724.79604033692	-1.93024754751904	0.047514062879711	XP_012552738.2	BTB/POZ domain-containing protein 9 isoform X2
LOC101739255	101.31227161983	-1.52566482354562	0.0479313341221905	XP_004924079.1	uncharacterized protein LOC101739255
LOC101740863	475.561840888759	-1.62305839162392	0.0488204293418131	XP_062524382.1, XP_037877838.1, XP_062524383.1	protein abrupt isoform X2, protein abrupt isoform X1, protein abrupt isoform X3
CPR13	39.1425050373321	-2.75794577600031	0.0488333283962243	NP_001166735.1	cuticular protein RR-1 motif 13 precursor

LOC101740267	2533.73378831015	-2.71367415245452	0.0488806880260475	XP_004923823.1	septin-2
LOC101747198	185.492763028979	-1.41941262156398	0.0495073743975372	XP_062526995.1	cytotoxic granule associated RNA binding protein TIA1
LOC134200674	1320.40739315166	-2.01971373637522	0.0495073743975372		
LOC101741145	2828.35379274131	-1.48418030578191	0.0495210199996935	XP_037869748.1,<br>XP_037869749.1	catenin alpha isoform X1,<br>catenin alpha isoform X2
LOC101738713	538.12743004627	-1.40363841872875	0.0496943739646741	XP_037870766.1	synembryn
LOC134200371	34.1787339217563	-1.20288850650774	0.0502915243730288	XP_037871580.1	uncharacterized protein LOC134200371
LOC101736081	729.018825670346	-3.69483915347472	0.0503155447289885	XP_062532237.1	protein glass
LOC101744081	654.542796301888	-1.09231188735522	0.0520010849921755	XP_037867757.2,<br>XP_037867756.2,<br>XP_037867758.2	protogenin B isoform X2,<br>protogenin B isoform X1,<br>protogenin B isoform X3
LOC134201164	139.151084613077	-1.48202573711349	0.0527250176885552	XP_062531469.1	uncharacterized protein LOC134201164
LOC110384794	196.336485267171	-1.74251168417128	0.0529719898093606	XP_021202040.1	ommochrome-binding protein
LOC134200535	14.5845901127817	-2.09868636442892	0.0530276331005292		
BAEE	368838.448835563	-1.80910643964506	0.0534189033807674	NP_001036844.1	CLIP domain-containing serine protease HP8 precursor
LOC110385289	35.2843765101255	-2.35398507839876	0.0534446170182043		
LOC134199432	10.4543588176573	-2.11790438589041	0.0540622589862484	XP_062526259.1	uncharacterized LOC134199432
Hsp90	4169.69470450551	2.13432733009186	0.054414248069246	NP_001036876.1	heat shock protein 83
LOC101744020	209.043304076106	-1.52526284997798	0.0545887832934741	XP_004923237.1	protein Tob1
LOC110385184	143.359996338129	-2.22938750132878	0.0556320640519392	XP_021203638.1	zinc finger protein 354C
LOC101745464	41.0946182033904	1.56271959017939	0.0557118673797419	XP_004924745.1,<br><br>XP_004924744.1	lysosomal proton-coupled steroid conjugate and bile acid symporter SLC46A3 isoform X2,<br>lysosomal proton-coupled steroid conjugate and bile acid symporter SLC46A3 isoform X1
LOC101742459	12598.2191348794	-1.88657022244246	0.0559099498688752	XP_004929485.1,<br>XP_062527386.1,<br>XP_004929486.1,<br>XP_062527385.1	striatin-3 isoform X1,<br>striatin-3 isoform X4,<br>striatin-3 isoform X3,<br>striatin-3 isoform X2
LOC105842461	25.1501004796038	-2.57140201742526	0.055932235392471	XP_012551117.2	uncharacterized protein LOC105842461
LOC101744741	653.46928190494	-1.4091559351652	0.0563163736874332	XP_037868390.1	GATOR2 complex protein MIOS
LOC101743223	6343.26898205912	-2.21444251288841	0.0567761735368248	XP_037867494.1,<br>XP_062524904.1,	mitogen-activated protein kinase kinase kinase 4 isoform X4, mitogen-activated protein kinase kinase kinase 4 isoform X7, mitogen-activated protein kinase kinase kinase

				XP_037867493.1, XP_037867492.1	4 isoform X3, mitogen-activated protein kinase kinase kinase 4 isoform X2
LOC101741664	7608.49650947321	-2.77760655629102	0.0567761735368248	XP_037874723.1, XP_062530980.1	calcium uptake protein 1 homolog, mitochondrial isoform X2, calcium uptake protein 1 homolog, mitochondrial isoform X3
LOC101738746	28.9779058128684	-2.46221037949434	0.0571468674654475	XP_062529553.1	probable aldehyde oxidase gad-3
LOC101742074	134.855143780309	1.00153810373578	0.0571569718736276	XP_004930989.1	neuroguidin
LOC101736817	111.435824642662	-1.09520416504955	0.057592247525303	XP_012549850.1	tetraspanin-11
LOC101742827	48.101797887144	-2.09362455340427	0.0576383267154903	XP_004925223.3	protein artichoke
LOC101738532	66.0007118624112	-1.37205654407954	0.0576383267154903	XP_062530410.1	uncharacterized protein LOC101738532
LOC101738302	92.8295917219212	-1.92450240960791	0.0577257321846547	XP_021203989.2, XP_062533051.1, XP_062533052.1, XP_062533053.1	uncharacterized protein LOC101738302 isoform X1, uncharacterized protein LOC101738302 isoform X1, uncharacterized protein LOC101738302 isoform X1, uncharacterized protein LOC101738302 isoform X2
LOC101745569	450.359575520234	-1.22256722892881	0.0577257321846547	XP_037870694.1, XP_037870693.1	tyrosine-protein kinase Btk isoform X2, tyrosine-protein kinase Btk isoform X1
LOC134200374	21.9252398042058	-1.82805470560693	0.0577257321846547		
LOC101744987	254.615897713101	-2.13743524455928	0.0577257321846547	XP_021204363.2	Bardet-Biedl syndrome 2 protein homolog
LOC101743588	266.370213515441	-1.00412610495965	0.0583532771017277	XP_062530781.1	ras-specific guanine nucleotide-releasing factor 1
LOC101740162	21.7596725206167	-2.30180408961877	0.0583532771017277	XP_004933590.3	facilitated trehalose transporter Tret1
LOC119629607	149.039641274184	-2.19547054375311	0.0583631608634493		
LOC101737300	344.311017435012	-2.05804189000061	0.0588177243785561	XP_004926790.1	uncharacterized protein LOC101737300
LOC101742475	125.079591527972	-1.21783167661601	0.0588177243785561	XP_004921819.1	actin maturation protease
LOC101735855	559.002057119471	-1.18065907548147	0.0589148848585879	XP_062526841.1	BTB/POZ domain-containing protein 7
Mppd	59.4172040907801	1.26493886460396	0.0595197663100613	NP_001093300.1	mevalonate diphosphate decarboxylase
LOC101746186	40.3546907076226	2.53722075229055	0.0610825833865819	XP_004925373.1	putative inorganic phosphate cotransporter
LOC101745030	1749.89664575111	-1.55123108206302	0.0615240184096395	XP_037872601.1, XP_037872596.1	UBX domain-containing protein 4 isoform X2, UBX domain-containing protein 4 isoform X1
OARpseudo	14.3678152518427	-2.45528766201747	0.061571439019503		
LOC119629351	16.7733323479949	-3.92145768732477	0.0619745114514698		
LOC101740835	40.5634233056218	-2.2949807941794	0.0621902647964991	XP_004931116.2	uncharacterized protein LOC101740835
LOC101743445	215.530235519515	1.10456407716058	0.0621902647964991	XP_004924159.1	serine palmitoyltransferase 1
LOC101737899	219.895565482973	1.71833724435635	0.062433864163937	XP_004922846.1	collagenase

LOC101736321	742.683439057553	-1.25332280802847	0.0626124869806166	XP_037875284.1	codanin-1
Or-53	15.236413439814	-2.02755170859547	0.062824276957192	XP_012550237.2, NP_001166615.1	olfactory receptor 53 isoform X1, olfactory receptor 53
LOC101739570	6583.60218791558	1.88967999870015	0.0631210405836393	XP_004922430.2	calphotin
LOC101746082	168.72336170691	-2.23100678909611	0.0638092062225084	XP_037876275.1	aldo-keto reductase family 1 member B1 isoform X2
LOC101740550	148.116527488132	-1.19005098032016	0.0641464023325766	XP_012548261.1	tetratricopeptide repeat protein 28 isoform X1
LOC101737618	58.1070493467375	1.29065893884405	0.0641464023325766	XP_012551985.4	LOW QUALITY PROTEIN: 3-oxoacyl-[acyl-carrier-protein] reductase FabG-like
LOC101735517	54.3758691294006	-1.56867681376956	0.0641464023325766	XP_021205044.2, XP_021205042.2, XP_012547671.2, XP_062529733.1	potassium voltage-gated channel protein eag isoform X2, potassium voltage-gated channel protein eag isoform X1, potassium voltage-gated channel protein eag isoform X3, potassium voltage-gated channel protein eag isoform X4
LOC101746597	1693.84865655497	-1.88319107054742	0.0644637203074366	XP_012551872.2, XP_062528690.1	serine proteinase stubble isoform X1, serine proteinase stubble isoform X2
LOC134198805	130.932525536911	-2.24155795817882	0.0647135890976052		
LOC134199652	154.368830319602	-2.66429195657881	0.0650103255335036		
LOC119631180	1203637.37800543	-1.22538088512046	0.0666644123534129		
LOC101745502	222.931599738041	-1.30955139580354	0.0677440238136276	XP_004921705.1	uncharacterized proteinLOC101745502
LOC134199764	1221951.25198353	-1.19788079166176	0.0697837789555246		
LOC119630254	13.9794079900159	-2.52024796371527	0.0698728411559897		
LOC101744825	237.332443864901	-1.69174915625825	0.0699535939478136	XP_037868808.1	TBC1 domain family member 5
LOC101740613	2319.32192158287	1.52295234035358	0.0699535939478136	XP_004932707.1	uncharacterized protein LOC101740613
LOC110385352	125.643749296622	-2.37627861161091	0.0701099596912274	XP_021204205.2	probable RNA-directed DNA polymerase from transposon X-element
LOC110385267	537.201754283235	-1.44503460001665	0.0708435887552599	XP_062525212.1	unconventional myosin-Ia
LOC101739649	228.539972537981	-2.55197699161855	0.0708435887552599		
LOC101737363	117.017762065185	1.09475269605367	0.0711972150548098	XP_004929849.1	proton-coupled folate transporter isoform X1
LOC110385843	693.426968023309	6.84413973049401	0.0713349657872425		
LOC101742233	36.2216608067428	-2.51131269489763	0.0719874560388495	XP_004931596.1	15-hydroxyprostaglandin dehydrogenase [NAD(+)]
LOC101742344	393.931335878242	-1.11186458255985	0.07208452610492	XP_037867467.1	splicing factor 1
LOC119630877	1266603.01498969	-1.21413167528386	0.0723100899522321		
LOC101743598	505.366859225384	1.92500340082615	0.0727671074382043	XP_004927569.1	uncharacterized protein LOC101743598
LOC101737151	37.6349556994391	-1.94757046117333	0.0729832955032998	XP_037868564.1	metabotropic glutamate receptor 2

LOC100500925	298.52799353287	-1.49443375967521	0.0732726449362537	XP_012546833.1	ionotropic GABA-aminobutyric acid receptor RDL3 isoform X2
LOC101745406	902.888053686767	-1.00805808298799	0.0735775252754547	XP_012551679.1, XP_004933086.1	histone H3.v1 isoform X4, myb-like protein X isoform X2
LOC101740363	50.4971596904764	-3.60389618503733	0.0739019993371472	XP_037872906.1, XP_012545571.1	uncharacterized protein LOC101740363 isoform X2, uncharacterized protein LOC101740363 isoform X1
RDL2	38.0681751959329	-2.15858634166918	0.0739652703147669	NP_001182629.1	ionotropic GABA-aminobutyric acid receptor RDL2 precursor
Mef2	2562.71784519152	-1.2083250955921	0.0742595697412974	NP_001036905.1	myocyte enhancer factor 2 isoform A
LOC101746463	90.929568211525	-1.45339074602672	0.0743347727155391	XP_021202405.2	radial spoke head protein 3 homolog isoform X2
LOC134199478	48.2953354454947	-2.13124720688529	0.0746294204076887	XP_062526431.1	uncharacterized protein LOC134199478
LOC105842676	45.241004773027	-1.43781603428272	0.0755540071895296		
LOC101742190	196.093787084175	-1.63673418749563	0.0760915502764935	XP_004922600.1	hypodermin-A
LOC101744478	1656.15782284246	-1.36093726156262	0.0762201140680217	XP_062527056.1	zinc finger CCCH domain-containing protein 15 homolog
LOC101742903	1650.22103692859	1.06253301580391	0.0767651358098335	XP_004931132.1, XP_062526145.1	synaptic vesicle glycoprotein 2B isoform X3, synaptic vesicle glycoprotein 2B isoform X1
LOC119631097	1318341.41126808	-1.19741437609847	0.0769163310369433		
LOC134199765	1318151.77338215	-1.19741161336458	0.0769163310369433		
LOC101737846	24.9542960963166	-2.28724261677991	0.0769163310369433	XP_062529221.1	DNA polymerase nu
LOC101741888	28.6546588136262	-1.30236707788953	0.0769163310369433	XP_062529888.1	Bardet-Biedl syndrome 4 protein homolog
LOC101744865	952.802049930682	-1.78795597780072	0.0774430704848375	XP_004931824.1	DNA-directed RNA polymerase, mitochondrial
LOC134199766	1313220.10518767	-1.19793296371729	0.0774430704848375		
LOC101741388	1880.02809108005	-2.74487635890465	0.0774467766768689	XP_037875162.1	tetratricopeptide repeat protein 7B
LOC101736798	74.6399080663701	-1.64378838238673	0.077474904951947	XP_037875830.1, XP_037875831.1	uncharacterized protein LOC101736798 isoform X1, pancreatic lipase-related protein 2 isoform X2
LOC101747088	15.4359320177234	-1.91518784476536	0.07861770890752	XP_004931356.1, XP_004931357.1	ataxin-2 homolog isoform X1, ataxin-2 homolog isoform X2
LOC134199191	174.304396291538	-2.21420343237461	0.07861770890752		
LOC105841746	651.754538946752	-1.22016526153749	0.0789145336820837	XP_037867926.2, XP_037867931.2, XP_062525474.1, XP_062525473.1,	uncharacterized protein LOC105841746 isoform X4, uncharacterized protein LOC105841746 isoform X1, uncharacterized protein LOC105841746 isoform X5, uncharacterized protein LOC105841746 isoform X3,

				XP_062525472.1, XP_062525475.1	uncharacterized protein LOC105841746 isoform X2, uncharacterized protein LOC105841746 isoform X6
LOC101746702	161.952778014549	1.49372856498983	0.0799108025215269	XP_012545371.2	mucin-2
LOC105841601	502.653604378254	-1.83784591766029	0.0802209502839406	XP_062529529.1, XP_062529530.1, XP_037873095.1, XP_037873096.1, XP_062529528.1	protein charlatan isoform X1, protein charlatan isoform X2, protein charlatan isoform X1, protein charlatan isoform X1, protein charlatan isoform X1
LOC134201141	76.5351319563379	-1.71574514277985	0.080311986922763		
LOC101742658	1007.68124354466	-2.27372141741171	0.0811841005394604	XP_037867972.1	E3 ubiquitin-protein ligase KCMF1
LOC101738340	175.901566256564	-1.6878765351225	0.0812705307744804		
LOC101740404	221.394260000611	-1.79739409914833	0.0816294611849418	XP_004925205.2, XP_021203016.2	methionyl-tRNA formyltransferase, mitochondrial isoform X1, methionyl-tRNA formyltransferase, mitochondrial isoform X2
LOC101738897	50.6827963069259	-1.52312956182296	0.0820519382157742	XP_012548605.1	uncharacterized protein LOC101738897
LOC110384829	1167.60089595095	-1.41106445012035	0.0828913984557531	XP_037874540.1, XP_037874532.1, XP_037874544.1	histone acetyltransferase KAT6A isoform X6, histone acetyltransferase KAT6A isoform X3, histone acetyltransferase KAT6A isoform X7
LOC101741073	80.3501882876348	1.25951099763895	0.083052961097546	XP_004929200.2, XP_037866476.1	homeobox protein aristaless isoform X1, homeobox protein aristaless isoform X2
LOC100862822	33.9429882253159	-1.47217704689529	0.083052961097546	XP_037870428.1	LOW QUALITY PROTEIN: UDP-glucosyltransferase 2
LOC101739585	248.554868582485	-1.90532836886024	0.0832984435566958	XP_037875840.1, XP_062531940.1	zinc finger protein 761 isoform X1, zinc finger protein 8 isoform X3
LOC134201756	38.1202231466325	-1.30361207410689	0.0833611186012106	XP_062532991.1	uncharacterized protein LOC134201756
PLCb4	1846.04611086421	-1.69131271845399	0.0839295530610072	XP_037873012.1, XP_037873013.1, NP_001165393.1	1-phosphatidylinositol-4,5-bisphosphate phosphodiesterase beta-4 isoform X2, 1-phosphatidylinositol-4,5-bisphosphate phosphodiesterase beta-4 isoform X1, 1-phosphatidylinositol-4,5-bisphosphate phosphodiesterase beta-4
LOC101746745	154.107446915534	3.35761086093975	0.0839295530610072	XP_004922544.1, NP_001296488.1	low molecular 30 kDa lipoprotein PBMHP-12-like isoform X1, low molecular 30 kDa lipoprotein PBMHP-12-like precursor

LOC101742275	18.0938863076714	-2.96407121228666	0.0839295530610072	NP_001296543.1	histidine-rich glycoprotein-like precursor
LOC119630902	1167042.38386514	-1.20177329001653	0.0842530797497748		
LOC101735662	796.431173204307	-1.37990618653337	0.0850718243799973	XP_037869376.1	peroxisomal acyl-coenzyme A oxidase 3 isoform X1
LOC101735324	2158.72665517425	-1.67505816583407	0.0850718243799973	XP_021203907.1, XP_004926568.1	leucine-rich repeat-containing protein 40 isoform X1, leucine-rich repeat-containing protein 40 isoform X2
LOC134200581	1582.73395046744	-1.15562725258488	0.0854679725779847	XP_062529713.1	uncharacterized protein LOC134200581
LOC101738629	63.0543276291657	-1.64063607687019	0.0857563747033429	XP_004930966.1	T-box transcription factor TBX20 isoform X1
LOC134199762	1313047.71794198	-1.16688896945983	0.0857563747033429		
LOC119630876	1313019.15104442	-1.16688278848897	0.0857563747033429		
LOC100302603	2634.42894387141	-2.8404688806663	0.0857899742360498		
LOC134199778	1307656.29294653	-1.16929655157126	0.0857899742360498		
LOC101744781	10.3658369733187	2.70257200984431	0.0857899742360498		
LOC119628413	95.4665196106677	-2.08797688762496	0.086008908521436	XP_037875338.2	metabotropic glutamate receptor 5
LOC134199161	9.98300995449269	6.0618526221948	0.0861944887731217		
LOC101739406	226.97176095799	-1.1051813960318	0.0865509411256094	XP_012546905.2	toll-like receptor 4
LOC101744814	400.15766048057	-1.07881968224332	0.0866536422089535	XP_012550501.2, XP_012550502.2, XP_037869840.1	putative 1-phosphatidylinositol 3-phosphate 5-kinase isoform X2, putative 1-phosphatidylinositol 3-phosphate 5-kinase isoform X3. putative 1-phosphatidylinositol 3-phosphate 5-kinase isoform X1
LOC101737910	435.616164808562	-1.00198217428598	0.0866536422089535	XP_062529438.1	uncharacterized protein LOC101737910
LOC101738479	9.50815311940702	-2.75545430520263	0.086665356876127	XP_037873186.1	Uncharacterized protein LOC101738479
LOC119630562	208.471761805055	1.60460455342421	0.0867700233512334	XP_062532032.1	zinc carboxypeptidase A 1
LOC119630957	1262011.26353434	-1.19051459423641	0.0870372401701036		
LOC100862778	2936.41617638247	1.73171545159717	0.0870372401701036	NP_001243934.1	putative cuticle protein CPH45 precursor
LOC119630958	1265122.87905661	-1.18849634949898	0.08715386051364		
LOC101740062	43.5631728081523	-1.6675021243762	0.0875228799128446	XP_062528423.1, XP_004928623.1	protein obstructor-E
LOC134199225	177.217445101439	2.01512695328164	0.0875316788152052		
LOC134199767	1269061.76657041	-1.1841777918107	0.0876601988628302		
LOC101743168	895.348673981573	-1.82874467779017	0.087721546366088	XP_037874306.1	AP-3 complex subunit beta-2
LOC119631189	952998.01159002	-1.18660507843696	0.0879428567945166		
LOC134199604	849.587118253244	-1.96862705406428	0.0879933542324972		

Noki	73.64402911353	-2.40380158478809	0.0879933542324972	NP_001037283.1	Noki protein
LOC101744642	163.006096374288	1.01170819400619	0.0883408796020235	XP_004924037.1	ELMO domain-containing protein 2
LOC101745889	953.112796473715	-1.75727939078825	0.0891692727029816	XP_037873641.1	dynein axonemal heavy chain 12
LOC100037444	268.682912144114	1.06842520022264	0.0896476642322316	NP_001091841.1	mitochondrial import inner membrane translocase
LOC101739550	27.6077250537188	-2.15996539509707	0.0896476642322316	XP_004929547.5	ecdysone oxidase
LOC101743204	496.385036263662	-1.08314210355897	0.0896476642322316	XP_037872726.1, XP_037872723.1	uncharacterized protein CG5098 isoform X2, uncharacterized protein CG5098 isoform X1
LOC101737307	1069.49783506975	-1.20804962736048	0.0897600073639003	XP_037872029.1	nuclear pore complex protein Nup205
LOC101741236	15.9340934920322	-2.14156187974948	0.0897600073639003	XP_037871768.1	ATP-binding cassette sub-family C member 4
CPR57	37.2657164580689	-1.14595816451096	0.0899239589882887	NP_001166700.1, XP_012548297.1	cuticular protein RR-1 motif 57 precursor, cuticular protein RR-1 motif 57 isoform X1
NGR-A5	31.8884185893933	2.02208755171559	0.0902708989333006	NP_001127740.1	neuropeptide receptor A5 precursor
LOC101743186	105.860894608465	-2.34182716610418	0.0907528785776276	XP_012549048.2	uncharacterized protein LOC101743186
LOC101736180	51.6238680923279	-2.4128071880747	0.0909475941315851	XP_012545533.1	NGFI-A-binding protein homolog
LOC119629018	305.552452625074	-1.22432600879328	0.0909475941315851	XP_037869328.1	uncharacterized protein LOC119629018
LOC101743722	89.6922973315901	-1.0764225496259	0.0909475941315851	XP_004924161.2	protein trapped in endoderm-1
LOC101740229	41.0712961563565	1.20312879026629	0.0909475941315851	XP_004926385.1	uncharacterized protein LOC101740229 isoform X1
LOC101747053	818.867078421774	-1.61238383209379	0.0909475941315851	XP_062529437.1	probable RNA-directed DNA polymerase from transposon BS isoform X1
LOC134200380	63.578841881694	-1.74253668372974	0.0909475941315851		
CPR95	64.3349411847582	-1.16449733628365	0.0909475941315851	NP_001036863.1	cuticular protein RR-2 motif 95 precursor
LOC101737992	295.878513254185	-1.44310795579665	0.0909475941315851	XP_062531988.1, XP_037876227.1	zinc finger protein 771 isoform X1, zinc finger protein 260 isoform X3
LOC101739627	1254.53429817297	-1.53059653553348	0.0912317504874171	XP_037871242.1, XP_037871243.1	calpain-15 isoform, calpain-D isoform X2
LOC101735914	1145.63391824267	-1.76020574314532	0.0915294365903378	XP_062529327.1	intraflagellar transport protein 140 homolog
LOC101741598	34.6287204858682	-1.3864632913847	0.0915294365903378	XP_012551206.2	uncharacterized protein LOC101741598
LOC101742150	92.1644057980119	-1.02698480736642	0.0921354729706972	XP_037874268.1	zinc finger protein ZFP2
LOC101739209	53.0220873000973	-1.84776730420283	0.0923812977036029	XP_021206683.1	receptor-type guanylate cyclase gcy-28
LOC101742317	64.8255898040091	-1.8386757625224	0.0926055556204704	XP_021207921.1	probable E3 SUMO-protein ligase RNF212
LOC101745727	501.903385986218	-1.55348042937189	0.0927900254710895	XP_004929718.1, XP_004929719.1	mucin-5AC isoform X1, mucin-5AC isoform X2
LOC119628553	35.4963649310886	-1.363193761885	0.092929363332695		

LOC134200790	2330.60988348687	-1.1828765437336	0.092929363332695	XP_062530406.1	uncharacterized protein LOC134200790
LOC101743929	269.415905468492	-2.10272854917372	0.0930912340090378	XP_004925782.1	DNA-directed RNA polymerase III subunit RPC4
LOC101744250	1965.60143581399	1.01558213425154	0.0937259044312263	XP_037868955.1	uncharacterized LOC101744250
LOC119629049	106.749225814585	-1.83630164556549	0.0937993395582687	XP_062527225.1	uncharacterized LOC119629049
LOC101741699	12.320402568443	-2.39453476612004	0.0937993395582687		
LOC134199757	112.187028941774	-1.97412948453803	0.0937993395582687	XP_062527277.1	uncharacterized LOC134199757
LOC101742185	56.6280645439068	1.31463102652994	0.0937993395582687	XP_004921817.1	uncharacterized protein LOC101742185
LOC101735847	73.8336737088941	-1.06213590761655	0.0940891544595706	XP_062532284.1	facilitated trehalose transporter Tret1-like
LOC101739443	136.807478891607	-1.286754255992	0.0941188124894638	XP_004924626.2,<br>XP_037871698.1	coiled-coil domain-containing protein 39 isoform X2,<br>coiled-coil domain-containing protein 39 isoform X1
LOC101741714	388.11713886607	1.46415677940264	0.0951020160517601	XP_004925552.1	ribonucleoside-diphosphate reductase large subunit
LOC101740574	1532.90550087746	1.6987385884367	0.0951020160517601	XP_037873699.1	uncharacterized protein LOC101740574
LOC134198741	40.0949524408129	-2.02005449395855	0.0958814447381929		
BmorLPTQ	8.57057932992045	-2.82639442674077	0.0958814447381929	NP_001274330.1	acyl-CoA Delta(11) desaturase-like
LOC105842243	25.1167073858492	-2.62873899017082	0.0959202444090169	XP_037869414.2	uncharacterized protein LOC105842243
LOC101737834	192.62357026458	1.31611491047738	0.0962838899263123	XP_004931376.2	trypsin, alkaline A
CPR52	14.1197022725975	-2.73533387156708	0.0962838899263123	NP_001166705.1	cuticular protein RR-1 motif 52
LOC119628453	4731.11604045975	-3.23799716250008	0.0964467431498537		
LOC110385119	109.345596623731	-2.15307867833716	0.0967234991201591		
LOC733023	59.3888967545686	1.27760246072475	0.0972849151111775	NP_001040483.1	replication factor C4
LOC101742557	26148.3542535288	-2.64036931820423	0.0979157804080046	XP_062526542.1	MICAL-like protein 1 isoform X2
LOC101743950	16.8601286350419	-2.26361229388942	0.0980403769550324	XP_004931415.1,<br>XP_062525393.1,<br>XP_062525394.1	atrial natriuretic peptide receptor 2 isoform X1,<br>atrial natriuretic peptide receptor 2 isoform X2,<br>atrial natriuretic peptide receptor 2 isoform X1
LOC101742434	2896.15367779453	-1.02136203875687	0.0980403769550324	XP_012552471.1,<br>XP_012552466.1	proline dehydrogenase 1 mitochondrial isoform X2,<br>proline dehydrogenase 1 mitochondrial isoform X1
LOC134201091	60.2236032205904	-1.73773327954312	0.0980403769550324		
LOC134200639	53.8226819879453	-1.44946028952953	0.0983893181731195		
LOC110386930	77.027994953162	-3.30164478681909	0.099092477902518		
LOC101738764	73.1434711416494	-1.21558946614272	0.0995122202445629	XP_004932015.1	T-related protein
Chbp	23257.8543946248	1.52678827300362	0.0997250121337508	NP_001037071.1	chlorophyllide A binding protein precursor
