# Supplementary material for: Transcriptomic Profiling Provides Insight into the Molecular Basis of Heterosis in Philippine-Reared Bombyx mori Hybrids
Source: Insects. 2025 Feb 26;16(3):243. doi: 10.3390/insects16030243 (PMC11942671; doi:10.3390/insects16030243)
Supplement: Supplementary file 1 [file insects-16-00243-s001.zip › Table S7 - DEGs associated with the Top 10 gene ontology (GO) terms in the comparison between parents and hybrids.pdf]

Table S7. DEGs associated with the Top 10 gene ontology (GO) terms enriched in upregulated and downregulated genes in the Philippine-reared *Bombyx mori* hybrids (NC144 and CN144) when parental strains (Lat21 and B221) are used as reference. GO terms were generated using g:Profiler for biological process, cellular component with a padj <0.05 filter, meanwhile ,the cut-off values for the DEGs were padj<0.1 and and |log2FoldChange|>1.

GO Term ID	Source	GO Description	Gene IDs of Associated DEGs
Upregulated genes in the hybrids (reference: Parental strains Lat21 and B221)			
GO:0042742	Biological Process	defense response to bacterium	ENB2, LOC101739536, CECB1, LOC101739958, LOC101739681, LZM, CECA, LEB3, LOC101743224, ATTACIN1, LOC101743336
GO:0009617	Biological Process	response to bacterium	
GO:0044183	Molecular Function	protein folding chaperone	HSP70, LOC100862772, HSP90, LOC101745500, LOC101746297, LOC101735832, LOC733093, LOC101745352, LOC101743498, LOC101741707, LOC791084
GO:0051604	Biological Process	protein maturation	HSP70, LOC100862772, HSP90, DNAJ-3, LOC101746297, LOC101735832, LOC733093, LOC778464, LOC101745352, LOC101743498, PPIB, LOC692906, LOC101741707, CRT, LOC791084, LOC101740246
GO:0006457	Biological Process	protein folding	HSP70, LOC100862772, HSP90, DNAJ-3, LOC101746297, LOC101735832, LOC733093, LOC101745352, LOC101743498, PPIB, LOC101741707, CRT, LOC791084, LOC101740246
GO:0140662	Molecular Function	ATP-dependent protein folding chaperone	HSP70, LOC100862772, HSP90, LOC101745500, LOC101746297, LOC101735832, LOC733093, LOC101743498, LOC101741707, LOC791084
GO:0098542	Biological Process	defense response to other organism	ENB2, LOC101739536, CECB1, LOC101739958, LOC101739681, PGRP, LZM, CECA, LEB3, LOC101743224, ATTACIN1, LOC101743336
GO:0043207	Biological Process	response to external biotic stimulus	
GO:0051707	Biological Process	response to other organism	
GO:0044419	Biological Process	biological process involved in interspecies interaction between organisms	
GO:0009607	Biological Process	response to biotic stimulus	

Downregulated genes in the hybrids (reference: Parental strains Lat21 and B221)			
GO:1901565	Biological Process	organonitrogen compound catabolic process	LOC101739798, LOC101742749, PAH, LOC101746583, LOC101747176, PGRP-S5, LOC101741659, LOC692784, LOC101738819, LOC101746090, LOC101741137, LOC101743217, LOC101744519
GO:0044282	Biological Process	small molecule catabolic process	LOC101742749, PAH, LOC101747176, LOC101741659, LOC692784, LOC101745938, LOC101741137, LOC101743217,
GO:1901606	Biological Process	alpha-amino acid catabolic process	LOC101742749, PAH, LOC101747176, LOC101741659, LOC101741137, LOC101743217
GO:0016491	Molecular Function	oxidoreductase activity	LOC101745562, LOC101740852, LOC692831, LOC101747037, XDH1, LOC101743494, LOC101736314, LOC101739179, LOC101746802, LOC101744406, LOC101746928, PAH, SDH2B, LOC101747176, LOC692784, LOC101739481, P450, CYP9A20, LOC101741137, CYP9A19, LOC101744260, LOC101744777, LOC101739276, LOC100302629, LOC101739328
GO:0009063	Biological Process	amino acid catabolic process	LOC101742749, PAH, LOC101747176, LOC101741659, LOC101741137, LOC101743217
GO:0170040	Biological Process	proteinogenic amino acid catabolic process	LOC101742749, PAH, LOC101747176, LOC101741659, LOC101743217
GO:0044248	Biological Process	cellular catabolic process	ATG8, LOC101742749, PAH, LOC101746583, LOC101747176, LOC101741659, LOC692784, LOC101738819, LOC101746090, LOC101741137, LOC101743217, LOC101744519
GO:0170035	Biological Process	L-amino acid catabolic process	LOC101742749, PAH, LOC101747176, LOC101741659, LOC101743217
GO:0009056	Biological Process	catabolic process	LOC101739798, ATG8, LOC101742749, PAH, LOC101746583 LOC101747176, PGRP-S5, LOC101741659, LOC692784, LOC101738819, LOC101745938, LOC101746090, LOC101741137, LOC101743217, LOC101744519
GO:1901575	Biological Process	organic substance catabolic process	LOC101739798, LOC101742749, PAH, LOC101746583, LOC101747176, PGRP-S5, LOC101741659, LOC692784, LOC101738819, LOC101745938, LOC101746090, LOC101741137, LOC101743217, LOC101744519
