# Supplementary material for: Transcriptomic Profiling Provides Insight into the Molecular Basis of Heterosis in Philippine-Reared Bombyx mori Hybrids
Source: Insects. 2025 Feb 26;16(3):243. doi: 10.3390/insects16030243 (PMC11942671; doi:10.3390/insects16030243)
Supplement: Supplementary file 1 [file insects-16-00243-s001.zip › Table S8 - DEGs associated with the Top 10 gene ontology (GO) terms in the comparison between the hybrids.pdf]

Table S8. DEGs associated with the Top 10 gene ontology (GO) terms enriched in upregulated and downregulated genes in the comparison between Philippine-reared *Bombyx mori* hybrids NC144 and CN144, with CN144 as reference. GO terms were generated using g:Profiler for biological process, cellular component with a padj <0.05 filter, meanwhile, the cut-off values for the DEGs were padj<0.1 and and |log2FoldChange|>1.

GO Term ID	Source	GO Description	Gene IDs of Associated DEGs
Upregulated Genes in NC144 (reference: CN144)			
GO:0000723	Biological Process	telomere maintenance	LOC119628747, LOC101738826, LOC119628780
GO:0032200	Biological Process	telomere organization	LOC119628747, LOC101738826, LOC119628780
GO:0003678	Molecular Function	DNA helicase activity	LOC119628747, LOC101738826, LOC119628780
Downregulated Genes in NC144 (reference: CN144)			
GO:0050794	Biological Process	regulation of cellular process	LOC101737089, LOC101747141, LOC101739770, INR, LOC101746180, LOC119629516, LOC101735723, LOC101742254, RPTOR, LOC105841689, LOC100302603, ABD-B, LOC101739974, LOC101741396, NANOSO, LOC101741238, LOC110386536, LOC101738201, LOC101738933, LOC101745749, LOC101743771, LOC101744662, LOC101736180, LOC101738468, LOC101740990, LOC101735602, LOC101746240, LOC101735930, NGR-B1, LOC101736789, LOC101743950, LOC101738029, LOC101739615, GATA-BETA, R2D2, LOC101743223, LOC778495, LOC101746458, LOC105841761, LOC101739274, LOC101735646, LOC119628413, LOC101742164, LOC101744757, SER-4, OR-53, LOC101745704, OR-59, LOC101737151, LOC101738302, LOC101735390, LOC101743730, LOC101740270, LOC101735982, LOC101739209, LOC101739475, SGF3, LOC101744528, LOC101744293, LOC101745630, PLCB4, LOC101737500, LOC101735441, LOC101738629, BMGEMININ, LOC101737509, LOC101743161, LOC110386833, LOC110384829, LOC101742582, NOTCH, LOC101744796, LOC101742593, LOC101745569, LOC101738764, MEF2, LOC101747187, LOC101739146, LOC101739406, OR, LOC101744814, LOC101743722, LOC101737382, LOC101743588
GO:0050789	Biological Process	regulation of biological process	LOC101737089, LOC101747141, LOC101739770, INR, LOC101746180, LOC119629516, LOC101740156, LOC101735723, LOC101742254, RPTOR, LOC105841689, LOC100302603, ABD-B, LOC101739974, LOC101741396, NANOSO, LOC101741238,
GO:0065007	Biological Process	biological regulation	

			LOC110386536, LOC101738201, LOC101738933, LOC101745749, LOC101743771, LOC101744662, LOC101736180, LOC101738468, LOC101740990, LOC101735602, LOC101746240, LOC101735930, NGR-B1, LOC101736789, LOC101743950, LOC101738029, LOC101739615, GATA-BETA, R2D2, LOC101743223, LOC778495, LOC101746458, LOC105841761, LOC101739274, LOC101735646, LOC119628413, LOC101742164, LOC101744757, SER-4, OR-53, LOC101745704, OR-59, LOC101737151, LOC101738302, LOC101735390, LOC101743730, LOC101740270, LOC101735982, LOC101739209, LOC101739475, SGF3, LOC101744528, LOC101744293, LOC101745630, SERPIN-13, PLCB4, LOC101737500, LOC101735441, LOC101738629, LOC101743372, BMGEMININ, LOC101737509, LOC101743161, LOC110386833, LOC110384829, LOC101742582, NOTCH, LOC101744796, LOC101742593, LOC101745569, LOC101738764, MEF2, LOC101747187, LOC101739146, LOC101739406, OR, LOC101744814, LOC101743722, LOC101737382, LOC101743588
GO:0035556	Biological Process	intracellular signal transduction	LOC101746180, RPTOR, LOC101741396, LOC101741238, LOC101738201, LOC101740990, LOC101735930, LOC101743950, LOC101739615, LOC101743223, LOC101746458, LOC101735646, LOC101740270, LOC101739209, LOC101739475, LOC101745630, PLCB4, LOC101745569, LOC101744814, LOC101743588
GO:0005509	Molecular Function	calcium ion binding	LOC101742624, LOC101745387, LOC101743074, LOC101741664, LOC101740975, LOC101741294, LOC101743222, LOC101739725, LOC101736444, ALG-2, LOC101739226, LOC101736510, LOC101737181, PLCB4, LOC101738867, MLC-2, LOC101736902, NOTCH, LOC733030, LOC101739353, LOC101746167
GO:0005515	Molecular Function	protein binding	LOC105842604, LOC100862826, LOC101746375, INR, LOC101746770, LOC110385547, LOC101742624, LOC101742972, RPTOR, LOC101736634, LOC105841612, LOC100302603, LOC110385321, LOC101738715, LOC101741388, LOC101745689, LOC101743627, LOC101736187, LOC101741950, LOC101741238, LOC101744691, LOC101742557, LOC101744789, LOC105842243, LOC101745037, LOC101744900, LOC101738080, LOC101739693,

			LOC101743174, LOC101740990, LOC101736718, LOC101744340, LOC101742925, LOC101738046, LOC101739495, LOC692743, LOC101747166, LOC101744987, LOC101739771, LOC101745654, LOC101742827, LOC101744221, LOC101735329, LOC692993, LOC101744990, LOC101744757, LOC101736444, LOC101739972, LOC101742761, LOC101745704, LOC101736349, LOC101738302, LOC101736446, LOC101741626, LOC101742459, LOC101744927, LOC105842097, LOC101745111, LOC101745226, LOC101739475, LOC101743536, LOC101739226, LOC101744698, LOC101735737, LOC101741109, LOC101744865, LOC101744498, LOC101737629, LOC101735914, LOC101747200, LOC101745889, LOC110384794, LASP, LOC105842623, LOC101745630, LOC101737181, LOC101735324, LOC101743372, LOC101740863, LOC101742874, LOC101737509, LOC101743161, LOC692748, LOC101745030, LOC101739624, LOC101747066, LOC101741145, LOC110386833, LOC101741117, LOC101743348, LOC101739367, LOC101744741, LOC101738472, LOC101736050, LOC101738342, NOTCH, LOC101737507, LOC101743656, LOC101741888, LOC101742593, LOC101737173, LOC101743453, LOC101745400, LOC101745569, MEF2, LOC101740550, LOC101736324, LOC101735855, LOC101739353, GAS2, LOC101739406, LOC101747115, LOC101744081, LOC101744550, LOC101745219, LOC101737382, LOC101743588
GO:0023052	Biological Process	signaling	INR,LOC101746180, LOC119629516, LOC101740156, LOC101742254, RPTOR, LOC101741396, LOC101741238, LOC110386536, LOC101738201, LOC101745749, LOC101743771, LOC101740990, LOC101746240, LOC101735930, NGR-B1, LOC101743950, LOC101739615, LOC101743223, LOC778495, LOC101746458, LOC105841761, LOC101735646, LOC119628413, SER-4, OR-53, LOC101745704, OR-59, LOC101737151, LOC101740270, LOC101739209, LOC101739475, LOC101745630, PLCB4, LOC101737500, LOC101743372, LOC101737509, LOC101743161, LOC101742582, NOTCH, LOC101742593,
GO:0007154	Biological Process	cell communication	

			LOC101745569, LOC101739146, LOC101739406, LOC101744814, LOC101743722, LOC101737382, LOC101743588
GO:0007165	Biological Process	signal transduction	INR, LOC101746180, LOC119629516, LOC101742254, RPTOR, LOC101741396, LOC101741238, LOC110386536, LOC101738201, LOC101745749, LOC101743771, LOC101740990, LOC101735930, NGR-B1, LOC101743950, LOC101739615, LOC101743223, LOC778495, LOC101746458, LOC105841761, LOC101735646, LOC119628413, SER-4, OR-53,LOC101745704, OR-59, LOC101737151, LOC101740270, LOC101739209, LOC101739475, LOC101745630, PLCB4, LOC101737500, LOC101737509, LOC101743161, LOC101742582, NOTCH, LOC101742593, LOC101745569, LOC101739146, LOC101739406, LOC101744814, LOC101743722, LOC101737382, LOC101743588
GO:0051171	Biological Process	regulation of nitrogen compound metabolic process	LOC101737089, LOC101747141, LOC101739770, LOC101735723, LOC105841689, LOC100302603, ABD-B, LOC101739974, NANOSO, LOC101741238, LOC101738933, LOC101744662, LOC101736180, LOC101738468, LOC101735602, LOC101736789, LOC101738029, GATA-BETA, LOC101739274, LOC101742164, LOC101744757, LOC101738302, LOC101735390, LOC101743730, LOC101735982, SGF3, LOC101744528, SERPIN-13, LOC101735441, LOC101738629, BMGEMININ, LOC110386833, LOC110384829, LOC101744796, LOC101738764, MEF2, LOC101747187, OR
GO:0080090	Biological Process	regulation of primary metabolic process	
