# Supplementary figures and images for: Transcriptomic Profiling Provides Insight into the Molecular Basis of Heterosis in Philippine-Reared Bombyx mori Hybrids
Source: Insects. 2025 Feb 26;16(3):243. doi: 10.3390/insects16030243 (PMC11942671; doi:10.3390/insects16030243)

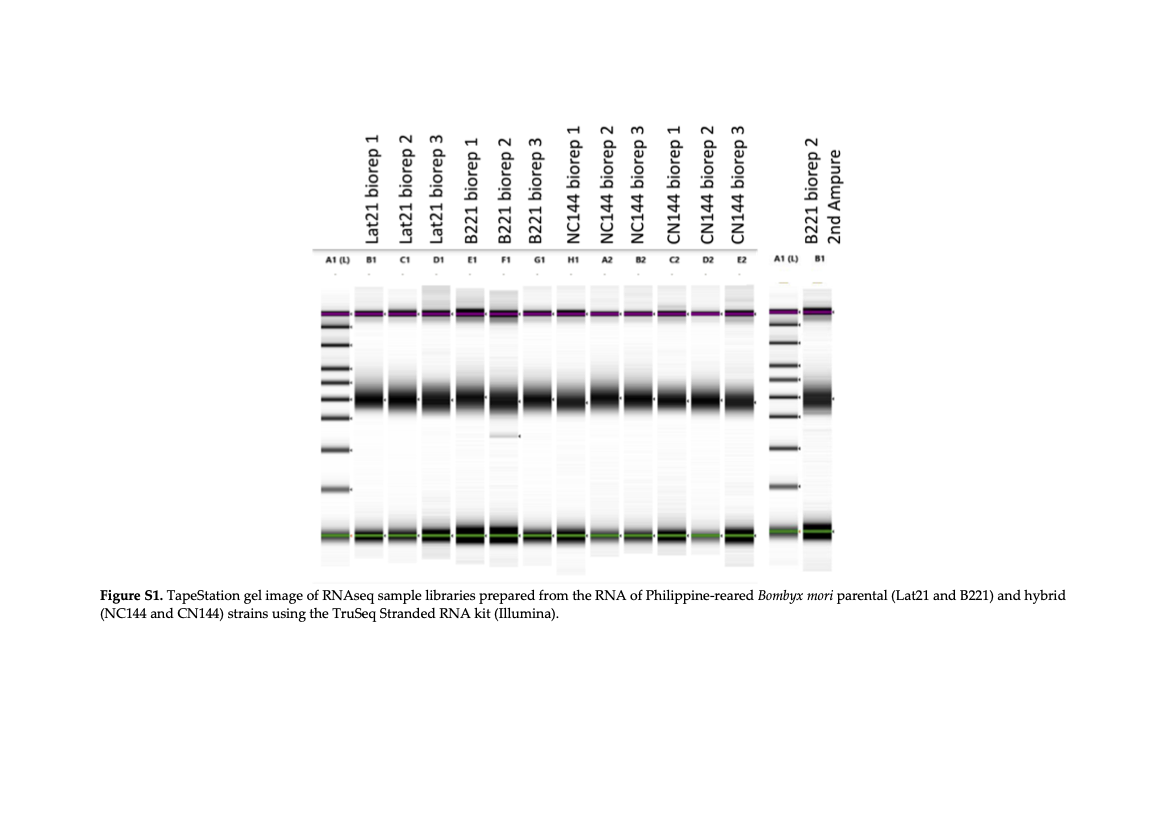

Supplement: Supplementary file 1 [file insects-16-00243-s001.zip › Figure S1.tiff]
